# Neonatal Maternal Deprivation Response and Developmental Changes in Gene Expression Revealed by Hypothalamic Gene Expression Profiling in Mice

**DOI:** 10.1371/journal.pone.0009402

**Published:** 2010-02-24

**Authors:** Feng Ding, Hong Hua Li, Jun Li, Richard M. Myers, Uta Francke

**Affiliations:** Department of Genetics, Stanford University, Stanford, California, United States of America; University of Nebraska Medical Center, United States of America

## Abstract

Neonatal feeding problems are observed in several genetic diseases including Prader-Willi syndrome (PWS). Later in life, individuals with PWS develop hyperphagia and obesity due to lack of appetite control. We hypothesized that failure to thrive in infancy and later-onset hyperphagia are related and could be due to a defect in the hypothalamus. In this study, we performed gene expression microarray analysis of the hypothalamic response to maternal deprivation in neonatal wild-type and *Snord116del* mice, a mouse model for PWS in which a cluster of imprinted C/D box snoRNAs is deleted. The neonatal starvation response in both strains was dramatically different from that reported in adult rodents. Genes that are affected by adult starvation showed no expression change in the hypothalamus of 5 day-old pups after 6 hours of maternal deprivation. Unlike in adult rodents, expression levels of *Nanos2* and *Pdk4* were increased, and those of *Pgpep1*, *Ndp*, *Brms1l*, *Mett10d*, and *Snx1* were decreased after neonatal deprivation. In addition, we compared hypothalamic gene expression profiles at postnatal days 5 and 13 and observed significant developmental changes. Notably, the gene expression profiles of *Snord116del* deletion mice and wild-type littermates were very similar at all time points and conditions, arguing against a role of *Snord116* in feeding regulation in the neonatal period.

## Introduction

Lifelong metabolic programming may depend on food availability during prenatal or early postnatal life. Environmental, epigenetic and genetic factors are likely to contribute [1]. Several inherited conditions are associated with infantile feeding difficulties and failure to thrive, such as Prader-Willi syndrome (PWS) [2], Kabuki (Niikawa-Kuroki) syndrome [3] and Börjeson-Forssman-Lehman syndrome [4]. Counter-intuitively, individuals with these disorders often develop obesity later in life. For example, after neonatal hypotonia and early feeding difficulties, children with PWS develop hyperphagia after two years of age that can lead to morbid obesity and associated ailments, including Type 2 diabetes mellitus, cardiovascular diseases and early death [5]. The underlying mechanism of the hyperphagic behavior is unclear. While stress-induced hyperphagia involves alterations of the hypothalamus-pituitary-adrenal (HPA) axis [6], no consistent abnormalities in the HPA axis have been reported in PWS individuals. It is intriguing to consider the possibility that the response to neonatal starvation could lead to abnormal hypothalamic gene regulation and later on to hyperphagia. Holland and colleagues [7] postulated that ineffective hypothalamic pathways may cause the body to interpret the absence of satiation as starvation.

Fasting-induced changes in hypothalamic gene expression have been studied extensively in adult rodents (reviewed in Morton 2006 [8]), and a systematic survey of gene expression profiles in adult rats after 48 hr fasting has been reported [9]. Hypothalamic expression levels of peptides known to be involved in appetite regulation, such as NPY, POMC, and AGRP, are changed upon food deprivation, and neurons expressing these molecules are essential components in the control of energy homeostasis [8]. Studies in neonatal rodents, however, are sparse. Of the genes with changed expression levels in fasted adults, only *Npy* was affected in food deprived mouse pups. Specifically, in the arcuate nucleus of the hypothalamus, NPY mRNA was increased after 8 hours of maternal deprivation in P8 mouse pups [10].

We hypothesized that the neonatal failure to thrive and the later onset hyperphagia in PWS could be explained by a defect in the hypothalamic response to food deprivation. To test this hypothesis, we generated a mouse model for PWS by deleting the *Snord116* cluster of imprinted snoRNAs, that are causally related to the PWS phenotype [11], on the paternally derived mouse chromosome 7 [12]. Adult *Snord116del* mice showed increased food intake, but did not develop obesity. In *Snord116del* mice fed *ad libitum*, levels of the orexigenic hormone ghrelin were greatly elevated in blood, to the same level seen in wild-type mice after 24 hr starvation. Ghrelin levels are also substantially raised in people with PWS [13], [14]. To assess the hypothalamic response to food deprivation, we performed gene expression microarray analysis of dissected hypothalami from 5-day old wild type and deletion mice, with and without prior starvation. To detect expression changes during early postnatal life, we compared hypothalamic gene expression profiles at postnatal days 5 and 13 in *Snord116del* mice and wildtype littermates. The results are of interest in three respects. First, we discovered that the hypothalamic response to starvation in neonatal mice is dramatically different from the starvation response reported for adult rats [9]. Second, we document significant developmental changes in hypothalamic gene expression between days 5 and 13. Third, the gene expression profiles of *Snord116* deletion mice and wild-type controls were very similar at all time points and conditions, arguing against a role of *Snord116* in feeding regulation in the neonatal period.

## Results

### Experimental Design

**Figure 1:**
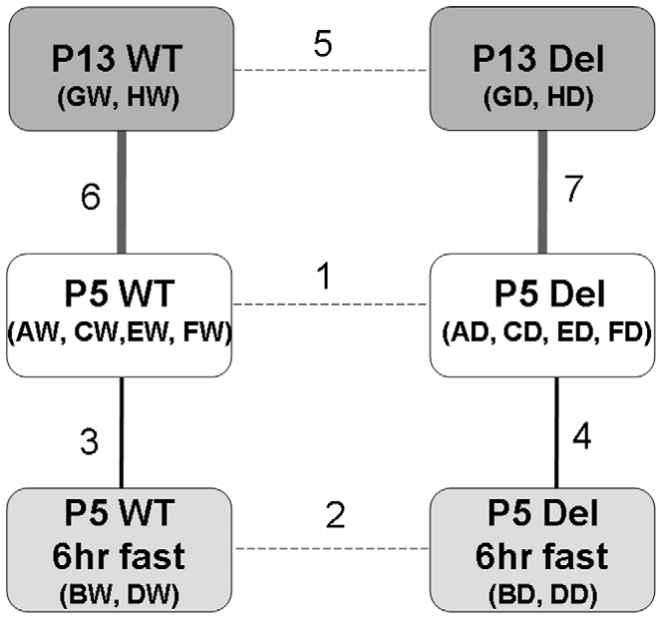
Experimental design and overall results of sample comparisons. Comparisons 1, 2 and 5 address the genotype difference; comparisons 3 and 4 address the effect of maternal deprivation; and comparisons 6 and 7 address developmental changes. The thickness of lines between sample boxes indicate the magnitude of expression differences between them. WT, wild-type controls; Del, *Snord116del* samples. P5, postnatal day 5; P13, postnatal day 13. Two-letter codes refer to the sample pools as detailed in Methods and Table 2.

The *Snord116del* mice have normal *in utero* growth and a normal birth weight, but they fail to grow normally after birth. At postnatal day 5 (P5), they begin to show a statistically significant weight difference with mice of both sexes weighing about 10% less than their wild type (WT) littermates. At P13, the deletion mice are about 40% lighter in weight, the largest weight difference in comparison to their WT littermates during development (Supp. Fig. S1). After weaning, the growth rate accelerates, but the mutant mice remain smaller for the rest of life [12]. Therefore, we chose P5 and P13 for gene expression microarray analysis. In previous studies of dissected brain regions we had noticed considerable random variation between individual samples. To reduce the false positives due to this random noise, we used sample pooling for our discovery array, and performed the follow-up validation by analyzing a larger number of well-matched individual samples. Fig. 1 illustrates the six datasets and the seven different types of pair-wise comparisons that we conducted.

### Fasting at Neonatal Stage Affects Hypothalamic Gene Expression

Changes in hypothalamic gene expression profiles in response to food deprivation had not been reported for neonatal mammals. Rat pups at 8–17 days postpartum showed compensational milk intake after 5 hr of maternal separation in an ambient temperature of 20°C [15]. Therefore, we chose a similar condition of 6 hr maternal deprivation for P5 mouse pups in an ambient temperature of 23–25°C. After 6 hr food deprivation, 106 probes showed increased expression and 112 probes decreased expression in WT samples (comparison 3, Table S1), and 109 probes showed increased and 121 probes decreased expression in *Snord116del* samples (comparison 4, Table S2), with fold change of >23% (2^0.3^) and t values >4. The Bonferroni-Hochberg False Discovery Rate (BH-FDR) is very high (0.171–0.389), indicating that many changes are likely due to random noise. Therefore, we focused on the genes that showed consistent changes in response to food deprivation in both sets of mice (comparisons 3 and 4 combined), since there are hardly any differences between the data for the two genotypes (see below).

**Figure 2:**
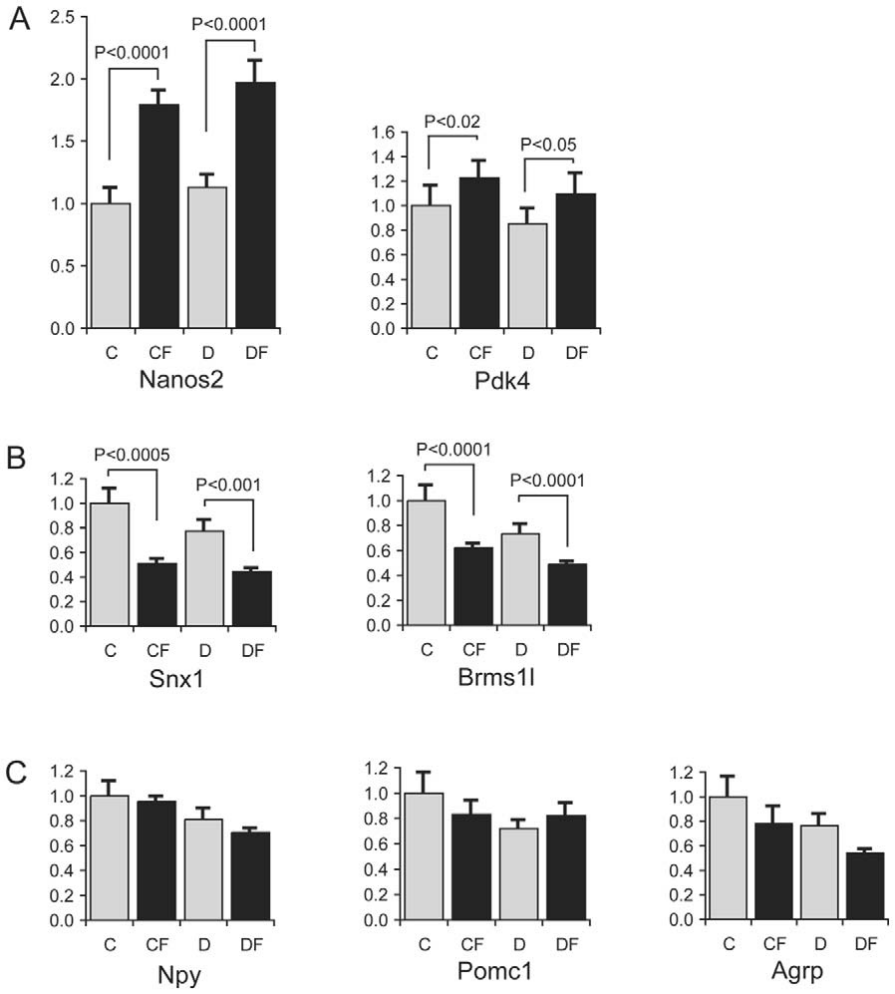
Real-time quantitative RT-PCR validation of gene expression changes detected by microarray studies after 6 hr fasting in P5 hypothalami. (C, control WT; CF, control WT after fasting; D, *Snord116del*; DF, *Snord116del* after fasting. N = 7–9 for each group). A. *Nanos2* and *Pdk4* transcript levels are increased after fasting for both genotypes. B. *Snx1* and *Brms1l* transcript levels are reduced after fasting for both genotypes. C. *Npy*, *Pomc* and *Agrp* transcript levels are not significantly changed after fasting.

Of the 8 genes with consistently increased expression after food deprivation in both genotypes, we chose five with high to modest expression levels for qRT-PCR validation and for only two genes, *Nanos2* and *Pdk4*, were we able to validate significantly increased expression after starvation in both WT and *Snord116del* hypothalami (Fig. 2A and Table 1). In the array data, we identified 14 genes with decreased expression in both genotypes after food deprivation. Five of the chosen six with high to modest level of expression (*Ing2*, *Pgpep1*, *Brms1l*, *Ndp*, *Snx1* and *Mett10d*) were validated by real-time RT-PCR analysis (all but *Ing2*) (Fig. 2B and Table 1).

**Table 1:** Genes with changed transcript levels after fasting in wild type (WT) and *Snord116del* (Del) hypothalami of postnatal day 5 mice.

Gene Symbol	Gene Name	Accession number Mouse	Change in WT		Change in Del		Li 2002*	
			Fold	P value	Fold	P value	Rat Gene ID	Result
***Increased***								
Nanos2	Nanos homolog 2 (Drosophila)	NM_194064.2	2.03±0.17	<0.0002	1.88±0.07	<0.0001	365213	NA
Pdk4	Pyruvate dehydrogenase kinase, isoenzyme 4	NM_013743.2	1.55±0.19	<0.02	1.53±0.12	<0.05	89813	N
***Decreased***								
Brms1l	Breast cancer metastasis-suppressor 1-like	NM_027867.2	0.74±0.04	<0.005	0.68±0.02	<0.01	299053	N
Mett10d	Methyltransferase 10 domain containing	NM_026197.1	0.62±0.04	<0.00001	0.66±0.04	<0.0005	360568	N
Ndp	Norrie disease homolog (Ndp)	NM_010883.2	0.62±0.08	<0.0005	0.62±0.11	<0.01	363443	NA
Pgpep1	Pyroglutamyl-peptidase I	NM_023217.2	0.61±0.11	<0.02	0.46±0.07	<0.0001	290648	N
Snx1	Sorting nexin 1	NM_019727.1	0.60±0.05	<0.0001	0.60±0.06	<0.001	84471	N

*Data reported for adult rats [9]. NA, gene not present on rat array, N, expression unchanged upon fasting.

### Effect of Food Deprivation in Young Pups Differs from That Reported in Adult Rodents

Surprisingly, the gene expression profile in P5 hypothalami after food deprivation is very different from that of the adult rodent [9]. In rat hypothalami after 48 hr fasting, analyzed with a 26,379 probe set (Affymetrix RG_U34A,B,C), Li and colleagues found 40 increased and 34 decreased transcripts with >20% change in expression levels and good consistency across replicate samples. To compare these data with our results, we searched for gene names associated with the GenBank accession numbers using DAVID (david.abcc.ncifcrf.gov). Of the 74 changed transcripts, 42 have known gene symbols and the rest are ESTs with unknown gene identity; and 37 of the 42 known rat genes have mouse orthologs that are present on the Illumina array of our study. None of these 37 genes, however, showed significant transcript changes after food deprivation in mice at P5. Conversely, of the seven genes we found to have validated expression changes after fasting in P5 mice, five have rat orthologs on Affymetrix RG_U34A,B,C chips (*Pdk4*, *Pgpep1*, *Brms1l*, *Mett10d* and *Snx1*) and the expression of these genes were unchanged in the adult rat brain after fasting (Table 1).

In adult mouse hypothalami, the expression of landmark genes such as *Npy*, *Pomc*1 and *Agrp* is changed upon food deprivation [16]. In the rat study, the *Npy* transcript level was significantly increased upon food deprivation, but *Pomc1* was unchanged and *Agrp* was absent from the array [9]. Our microarray experiments with neonatal hypothalami, however, revealed no consistent changes of any of these genes after food deprivation at P5. We, therefore, tested the expression of these candidate genes by real-time RT-PCR, and found no significant changes in expression levels in P5 hypothalami after fasting in either WT or *Snord116del* samples (Fig. 2C).

### Dramatic Changes in Neonatal Hypothalamic Gene Expression between P5 and P13

**Figure 3:**
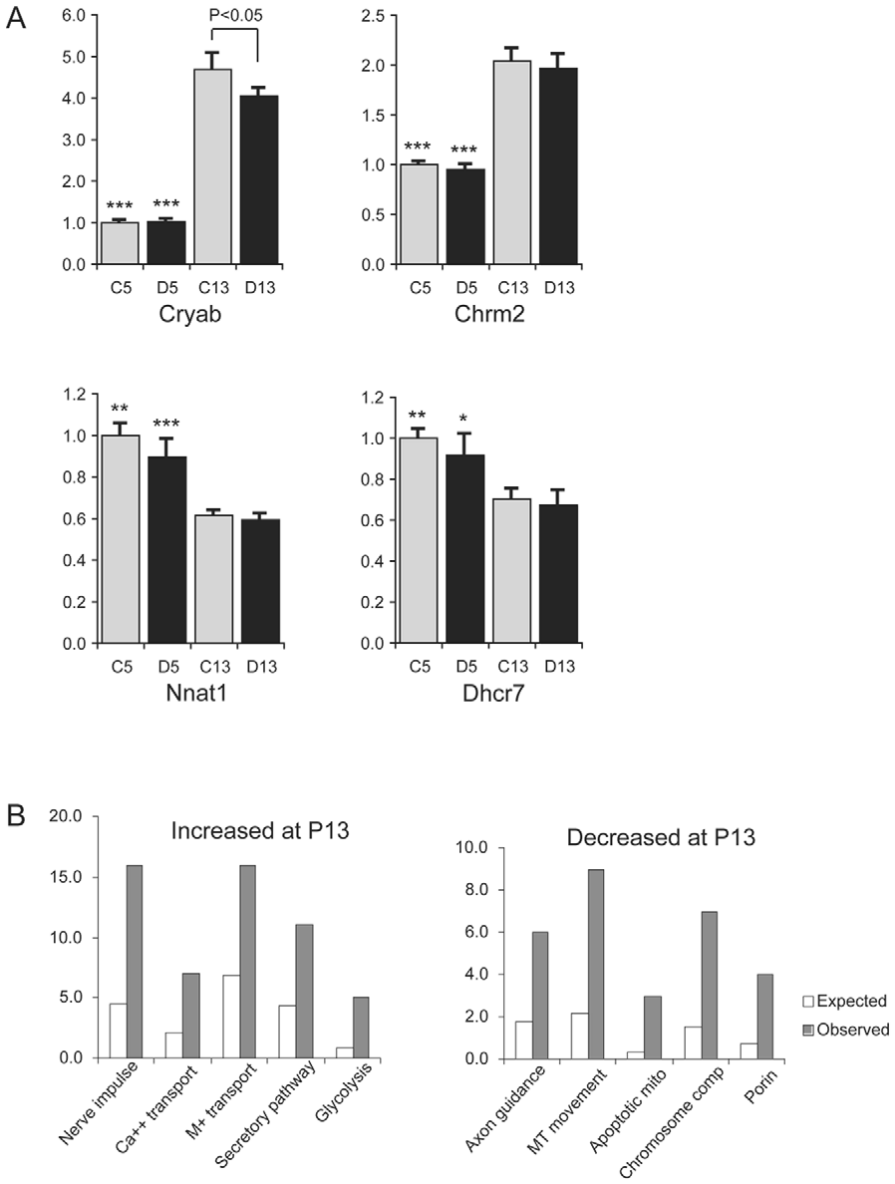
Comparison of hypothalamic gene expression patterns at P5 and P13. A. Quantitative RT-PCR analysis of genes with altered expression at P5 compared to P13. *Cryab* and *Chrm2* are increased at P13 vs. P5 for both genotypes, and *Nnat1* and *Dhcr7* are decreased at P13 vs. P5 for both genotypes. C13, control WT at postnatal day P13; D13, *Snord116del* at P13; C5, control WT at P5; D5, *Snord116del* at P5. (N = 7–9 for each group). Asterisks: significant differences between the two developmental stages for the same genotype (*, *p*<0.05; **, *p*<0.005; ***, *p*<0.0001). B. Gene ontology analysis. Left panel: Over-represented Gene Ontology categories of genes with increased expression at P13 vs. P5. Nerve impulse, *p*<1.08×10^−5^; Ca++ transport, *p*<0.0042; M+ transport: monovalent ion transport, *p*<0.0015; Secretory pathway, *p*<0.0044; Glycolysis, *p*<0.0018. Right panel: Over-represented Gene Ontology categories of genes with decreased expression at P13 vs. P5. Axon guidance, *p*<0.008; MT movement: microtubule-based movement, *p*<0.00032; Apoptotic mito: apoptotic mitochondria, *p*<0.0037; Chromosome comp: chromosome components, *p*<0.00071; Porin, *p*<0.0063.

To assess developmental changes in hypothalamic gene expression, we compared the profiles of WT pups at P5 and P13 (comparison 6, Fig. 1). Initially, we observed 796 probes with increased and 855 with decreased expression at P13 relative to P5 with fold of changes >23% (2^0.3^) and t values >4. Limiting the analysis to those with a false discovery rate (FDR) of <0.05 left us with 431 probes with increased and 447 probes with decreased expression, which correspond to 331 and 332 genes, respectively, in the 2008 release of LocusID (Table S3). We tested the expression of seven selected genes by real-time RT-PCR, and were able to validate the developmental changes between P5 and P13 in six of them (see examples in Figure 3A). *Cryab* and *Chrm2* showed a significant increase in expression levels at P13 vs. P5 (P<0.0001 for both WT controls and *Snord116del* samples). *Nnat1* (P<0.001), *Dhcr7* (P<0.05), *Dlk1* (P<0.001) and *Gfap* (P<0.05) showed significant decreases in transcript levels at P13 (Table S4).

The Gene Ontology categories enriched among the genes with increased expression in Comparison 6 (WT P13 vs. P5) are depicted in Fig. 3B, left panel. For genes with increased expression at P13, those that are involved in nerve impulse transmission, metal ion transport, secretory pathways and glycolysis are enriched. Genes with decreased expression at P13 vs. P5 are depicted in Fig. 3B, right panel. Genes that are involved in axon guidance, microtubule-based movement and alpha-actinin binding are over-represented. Genes involved in DNA replication, chromosome condensation and segregation are also overrepresented among genes with decreased expression at P13, as are those for of cyclin-dependent protein kinase inhibitors, apoptotic mitochondrial changes and nucleoporins.

### Expression Profiles of *Snord116del* Mice Are Not Different from Wild Type Littermates

In Comparison 1 (Fig. 1) on P5 non-fasted mice of the two genotypes, the microarray data showed only seven genes with increased (*Ang3*, *V1re8*, *Wscd2*, *Ctnnbl1*, *Rnf167*, *Fmo4* and *Pot1b*), and eight genes with decreased expression (*Prpf3*, *Ccnb1*, *Actrt1*, *Gtl28d1*, *Oflr259*, *Lrrc39*, *Fryl* and *ORF9*) in *Snord116del* pups with fold changes greater than 23% (2^0.3^) and t values >4. The minimal false discovery rate was in the range of 0.457–0.5. For validation, we analyzed the expression of three genes in each group by reverse transcription-real time PCR (qRT-PCR) using matched individual hypothalamic RNA samples. The expression levels of *Wscd2*, *Fmo4*, *Actrt1*, *Gtl28d1*, and *ORF9* were either too low to generate reliable data, or failed to reveal significant differences due to large standard deviations. *Ctnnbl1* expression levels were robust but not different when 16 *Snord116del* and 16 WT hypothalamic RNA samples were compared (data not shown). Therefore, we conclude that the gene expression profile of *Snord116de*l hypothalami at P5 is very similar to that of WT littermates, and that the changes observed in the microarray data are likely due to random noise.

In Comparison 2 on fasted P5 samples of the two genotypes, we did not find any gene with validated change in expression, either. Microarray data indicated 51 genes with increased expression and 49 genes with decreased expression with a high false discovery rate (FDR = 0.221–0.5). Because the two genotypes showed very similar expression without starvation, we reasoned that the true difference in Comparison 2 most likely come from genes that are affected by starvation.

Of the 51 genes with increased expression in fasted *Snord116del* hypothalami, 8 were decreased in Comparison 3 (WT non-fasted vs. fasted) but unchanged in Comparison 4 (*Snord116del* non-fasted vs. fasted), and 4 genes were increased in Comparison 4 but not in Comparison 3. Of the 49 genes with decreased expression in fasted *Snord116del* samples, one was increased in Comparison 3 (WT non-fasted vs. fasted) but unchanged in Comparison 4 (*Snord116del* non-fasted vs. fasted), and two genes were decreased in Comparison 4 but unchanged in Comparison 3. We selected eight genes (one to three with good expression levels from each group) for qRT-PCR validation. None the eight genes, (*Art5*, *Kif21b*, *Mospd3*, *Mrgprb5*, *Pkd1*, *Prkag3*, *S100a1*, *Snx1*), however, showed a significant difference in gene expression between the two genotypes after fasting. Although in the microarray data *Snx1* is significantly decreased in WT and insignificantly decreased in *Snord116del* samples after food deprivation, the real-time qRT-PCR assay revealed that it is significantly down-regulated in both genotypes.

In Comparison 5 at P13, gene expression profiles were also similar between *Snord116del* and WT hypothalami. There are 111 genes with increased expression and 129 genes with decreased expression in *Snord116del* hypothalami at this stage, with a relatively high false discovery rate (>0.59). Therefore, we chose seven representative genes (*Cryab*, *Chrm2*, *Nnat1*, *Dhcr7*, *GFAP*, *Dlk1*, *Pick1*) for real-time qRT-PCR validation (Fig. 3A, and data not shown). No differences between the two genotypes at P13 could be validated, except for a marginally significant increase in expression of *Cryab* in *Snord116del* hypothalami at P13 (P = 0.0495).

When the expression profiles of *Snord116del* hypothalami at P13 and P5 were compared (Fig. 1, Comparison 7), 365 probes showed increased expression at P13, and 310 probes showed decreased expression at P13, with fold-changes greater than 23% (2^0.3)^, t>4, and FDR<0.05). Gene ontology analysis revealed categories of changes similar to those seen for the WT samples, which is expected as the expression profiles were similar for the two genotypes at both developmental stages.

## Discussion

### Neonates Have a Dramatically Different Response to Fasting in Comparison to Adults

Neonatal rodents are able to regulate their milk intake by compensatory feeding after fasting [15], yet the underlying mechanism is not well understood, and as far as we know, our study is the first report of gene expression changes in fasting neonates. Interestingly, neonates have a dramatically different fasting response than do adult rodents. Although maternal separation in ambient temperature leads to compensational milk intake afterwards, this effect is not reflected in the change of hypothalamic gene expression profile of the neonates in the same way as was reported for adults. The hypothalamic genes known to change expression upon fasting in adults, namely *Npy*, *Pomc* and *Agrp* [9], [16], are unchanged after 6 hours of fasting in P5 pups. It is possible that the time of fasting is too short to affect expression of these genes, for a fasting of 24–48 hours is required to detect changes of expression in adults. But we detected transcription changes in unexpected genes, *Nanos2* and *Pdk4*, which are increased by roughly 2-fold and 1.5-fold respectively, and *Ndp*, *Pgpep1*, *Brms1l*, *Mett10d* and *Snx1*, which are reduced by 25–55% (Figure 2 and Table 1).

### Maternal Separation: Stress vs. Starvation

The effect of maternal deprivation inevitably leads to nutrient deprivation. Additionally, it also removed a heat source and other maternal stimuli. Therefore, the observed changes in hypothalamic gene expression profiles in starved pups may not solely be due to lack of caloric intake. In postnatal day 8 or 9 mice, prolonged (>4 hr) maternal separation leads to increased stress as indicated by elevated corticosterone and ACTH in the circulation, as well as reduced corticotropin releasing hormone (CRH) expression in the arcuate nucleus of the hypothalamus [10], [17]. Detailed analysis of the origin of stress revealed that metabolic effects are a major component. In P8 pups after 4hr or 8hr of maternal separation, glucose injection led to a 40% and 60% reduction in corticosterone level, respectively, and also reduced the changes in circulating ACTH and hypothalamic CRH levels by half [10]. Neuropeptide Y (*Npy*) mRNA in the arcuate nucleus was increased by about 40% after 8 hr of maternal deprivation. In further experiments, Schmidt and colleagues showed that in *Npy* deficient P8 mice deprived for 8hr, the decrease in hypothalamic CRH was attenuated, indicating that the *Npy*-mediated energy homeostasis pathway is crucial for the stress response in neonatal mice [18]. In rat pups at P8, maternal deprivation versus food deprivation with a non-nutritive dam still taking care of the pups, lead to identical changes in corticosterone levels [19]. Although these experiments were performed with older (P8) mice or rats, it is reasonable to assume that nutrient deprivation is the major contributor to the stress effects of maternal separation in P5 rodents as well.

Based on the published data cited above, we would have expected to see increased *Npy* and decreased *Crh* expression in the hypothalami of deprived pups. No such changes were detected in our microarray analysis. There are several possible explanations. Expression changes in these genes may not yet be obvious in the younger (P5) mice and/or not after only a 6 hr fast, as Schmidt and colleagues studied P8 mice after an 8 hr fast [10]. In addition, we studied gene expression in a much larger brain region, the entire hypothalamus instead of just the arcuate nucleus or paraventricular nucleus, and used a cut off of 23% (2^0.3^) change in the analysis of the microarray data. These factors could have obscured a small change in gene expression that may be specific to the arcuate nucleus for *Npy* and the paraventricular nucleus for *Crh*. Future studies on dissected nuclei from the hypothalamus will be required to evaluate this possible explanation.

Another potential effect of maternal separation on the P5 litters is the lack of maternal thermal contribution which could exaggerate the effect of energy deficiency. Singly housed rat pups were not able to maintain stable body temperature in a 24°C environment which is similar to the ambient temperature in our study [20]. The major source of heat generation during neonatal thermoregulation is from brown adipose tissue [21], and the pups showed increased oxygen consumption when the environmental temperature was below 32–36°C, presumably by burning brown adipose tissue [20]. Although in our study the pups were group-housed and huddled together, the six hours of maternal separation could lead to hypothermia, the response to which may have contributed to the observed hypothalamic changes in gene expression. Hypothermic effects on hypothalamic gene expression have not been systemically studied for either adult or neonatal rodents. Therefore, we can only speculate that the observed changes in gene expression indicate the compound effect of cold exposure and lack of food consumption, both of which should call for preservation and/or generation of energy, and that the hypothermia would exaggerate the effect of food deprivation alone. In addition, we cannot exclude an independent mechanism for temperature-dependent changes in the hypothalamic gene expression profile. Although in P12 rats after 24 hr maternal separation, the ultrasonic vocalization of the pups were independent of ambient temperature ranging from 23 to 35°C, no studies of temperature effects in younger rodents has been reported [22].

In summary, the changes in gene expression we detected and validated represent the most robust changes, which are neither in the feeding regulation nor the stress response categories. This is indeed a plus for microarray-based assay, as it is unbiased by previous knowledge, and can lead to new discoveries of gene function and regulation.

### Potential Functions of Genes with Changed Expression upon Neonatal Maternal Deprivation

*Nanos2* is one of three mammalian homologs for *Nanos* in Drosophila. During Drosophila egg development, *Nanos*, together with the RNA binding protein *Pumilio (Pum)*, represses the translation of maternal *hunchback* (*hb*) mRNA in the posterior region of the embryo, which allows abdominal development [23], [24]. In mice, *Nanos2* is critical for male germ cell development, as *Nanos2* knock-out males have smaller testes and a complete loss of spermatogonia [25]. *Nanos2* is a zinc finger protein residing in the cytoplasm, which was speculated to be involved in RNA binding and translational suppression similar to its Drosophila homolog, although direct experimental proof is still lacking. Interestingly, the 3′UTR of *Nanos2* mRNA contains regulatory elements that determine *Nanos2* protein levels, presumably by modulating its translational efficiency [26].

In hypothalamus, *Nanos2* is expressed modestly at P5 and P13. The function of *Nanos2* in brain development is unknown, as the *Nanos2* knock-out mice were apparently normal except for male infertility [25], and no phenotypic abnormality was reported for *Nanos2* transgenic mice, which over-express *Nanos2* under the control of Oct4DeltaPE promoter, but the expression was not examined in brain [27]. The genes that are regulated by *Nanos2* during germ cell development, such as *Stra8*, *Dnmt3L* and *Tdrd*, are unchanged in hypothalamus after neonatal starvation in our microarray data set. A different regulatory network may be present in the neonatal brain and it would be of interest to know whether *Nanos2* knock-out mice are intolerant to neonatal starvation.

The up-regulation of *Pdk4* may reflect an attempt to conserve energy during neonatal fasting. *Pdk4*, a member of the *PDK/BCKDK* protein kinase family, encodes a mitochondrial protein with a histidine kinase domain. This protein is located in the mitochondrial matrix and inhibits the pyruvate dehydrogenase complex by phosphorylating one of its subunits, thereby contributing to the regulation of glucose metabolism [28]. *PDK4* mRNA was increased approximately 3-fold after 15 hr and approximately 14-fold after 40 hr of fasting in adult human skeletal muscle [29]. In adult rat hypothalami after 48 hr fasting, however, elevation of *PDK4* was not observed (Table 1) [9]. This difference could be explained by adult brain being almost entirely dependent on glucose as a fuel source, thus giving it the highest priority for glucose supply. Starvation in adults leads to increased *PDK4* expression in heart, skeleton muscle, kidney and liver to reduce glucose consumption in these organs, in order to preserve glucose for the usage of brain. In neonates, however, a modest 6 hr fast is sufficient to induce an increase in *Pdk4* expression, indicating that the neonatal brain is not spared from glucose restriction during energy crisis. The physiological significance of this restriction may relate to the fact that the neonatal brain's major source of energy are ketones derived from fatty acid metabolism [30].

*Ndp* (previously *Ndph*) is the mouse homolog of the human gene that is mutated in Norrie disease, an inherited disorder with vascular defects of eyes that leads to blindness, progressive hearing loss and mental retardation. Norrin, the protein product of *Ndp*, is a high-affinity ligand for Wnt co-receptors FRIZZLED 4 and LRP 5 [31]. *Ndp* is expressed in many brain regions in the adult, but its expression in hypothalamus has not previously been reported [32]. We found a modest decrease of *Ndp* transcripts in the hypothalami of deprived pups, without changes in *Frizzled4* and *Lrp5* transcript levels. Ndp may be involved in the signaling of starvation response in the hypothalamus of young pups, or it may contribute to the disruption in brain development during starvation.

*Pgpep1*, Pyroglutamyl peptidase I (EC 3.4.19.3) is an enzyme that catalyzes the hydrolysis of N-terminal pyroglutamyl (pGlu) residues from oligopeptides and proteins, such as thyroid hormone releasing hormone (TRH) and neurotensin [33]. The N-terminal pGlu residue is known to protect the peptide from degradation by aminopeptidase, as well as to determine overall peptide function. For example, the pGlu residue of TRH interacts directly with the TRH receptor, and substitution of the pGlu reduces TRH's affinity for the receptor and decreases its potency [34]. In a fractionation study of human and rat brains, the highest enzymatic activity of Pegpep1 was found in the synaptosomal and myelinic fractions [35], and it was proposed that the enzyme may be involved in the inactivation of neuropeptides. In our neonatal starvation study, we observed that the *Pgpep1* transcript was decreased to about half normal levels, which could potentially prolong the function of TRH and neurotensin. Due to the wide substrate specificity, additional peptides may be affected. This could be one of the pathways by which the neonatal starvation leads to defects in neural development.

*Brms1l*, breast cancer metastasis-suppressor 1-like, is a gene with sequence similarity to *Brms1* but its function is unknown [36]. BRMS1 reduces the metastatic potential, but not the tumorigenicity, of human breast cancer and melanoma cell lines [37]. The protein encoded by this gene localizes primarily to the nucleus and is a component of the *Sin3a* family of histone deacetylase complexes (HDAC), although this interaction is not essential for its metastasis-suppressor function [38]. *In vitro*, over-expression of *BRMS1* decreased cancer cell survival under hypoxic stress, decreased the adhesion ability of cancer cells and increased apoptosis [39]. *Brms1l* is also a component of the *Sin3a* family, and if present in an HDAC complex the reduced expression we observed at P5 may contribute to regulating the expression of multiple genes in response to the starvation stress.

*Mett10d*, methyltransferase domain 10 containing protein, contains a domain belonging to the class I AdoMet-MTases: S-adenosylmethionine-dependent methyltransferases (SAM or AdoMet-MTase) superfamily, and the Predicted O-methyltransferase subfamily. *Mett10d* homologs have been identified in all vertebrates studied, including human, mouse and zebrafish. The mouse Mett10d protein sequence is 90% identical to the human protein, and 70% identical to that of the zebrafish. *Mett10d* is expressed in multiple mouse tissues, and its high degree of sequence conservation indicates that it may have essential functions in development or response to stress. Assessment of the involvement of Mett10d in neonatal starvation response awaits additional study of its function and natural substrates.

*Snx1*, sorting nexin 1, is a member of the sorting nexin family. Members of this family, including Snx1, Snx2, Snx5 and Snx6, form retromer complexes that mediate retrograde transport of transmembrane cargo from endosomes to the trans-Golgi network [40]. Retromer function has been implicated in many physiological processes, including setting up cell polarity, transcytosis, and regulating cell surface expression of cytokine receptors. After neonatal starvation, we observed decreased expression only of *Snx1*, but not of other sorting nexin genes such as *Snx2*, *Snx5* and *Snx6*. In HeLa cells, SNX1 is essential for the degradation of the G-protein coupled receptor PRP1 (protease-activated receptor-1) [41]. The function of *Snx1* in the nervous system has not been well studied, and we speculate that the decreased *Snx1* expression we discovered may affect the localization and/or abundance of membrane proteins involved in the development of neonatal brain.

In conclusion, the changes in neonatal hypothalamic gene expression after starvation reflect a very different response from that in adult rodents. As the neonatal brain is still developing and the wiring of the feeding regulation circuits in the hypothalamus are not complete until two weeks of age [42], it is necessary for the neonatal brain to devise special mechanisms to deal with the energy crisis. On one hand, the changes in the gene expression profile reflect the organism's attempt to reduce energy expenditure and put a hold on cell proliferation to survive the crisis; on the other hand, these changes may have long-lasting effect on the structure and function of the brain later in life.

### Developmental Changes of Gene Expression between P5 and P13

Gene ontology (GO) analysis provides a picture of the developmental changes in hypothalamic gene expression between day 5 and day 13 of postnatal life (Figure 3), which is similar to the reported changes in other brain areas. Of the genes with increased expression at P13 versus P5, many are involved in nerve impulse transmission. Some of their gene products are involved in axon myelination, including myelin basic protein (*Mbp*), claudin 11(*Cldn11*), proteolipid protein 1 (*Plp1*) and UDP galactosyltransferase 8A (*Ugt8a*). Other GO categories that are enriched include genes involved in metal ion transport and Ca^2+^-binding and transport, such as ATPase Ca^2+^-transporting, cardiac muscle fast twitch 1 (*Atp2a1*), calcium/calmodulin-dependent protein kinase II alpha (*Camk2a*), inositol 1,4,5-triphosphate receptor 1 (*Itpr1*), stromal interaction molecule 1 (*Stim1*), transient receptor potential cation channel, subfamily M, member 2 (*Trpm2*), and solute carrier family 24 (sodium/potassium/calcium exchanger), member 3 (*Slc24a3*). Multiple genes that function as transporters for monovalent ions, such as sodium and potassium ions, are also detected. Genes that function in secretory pathways responsible for the delivery of neurotransmitters are enriched, such as complexin 1 (*Cplx1*), palmitoyl-protein thioesterase 1 (*Ppt1*), syntaxin 4A (*Stx4a*) and synapsin II (*Syn2*). This correlates with increased neural signaling at this stage. Genes involved in glycolysis are also enriched, including aldolase 3, C isoform (*Aldoc*), enolase 2 gamma neuronal (*Eno2*), malate dehydrogenase 1, NAD (soluble) (*Mdh1*) and oxoglutarate dehydrogenase (lipoamide) (*Ogdh*). This pattern of change in gene expression reflects the switch from neonatal lipid metabolism to carbohydrate fuel usage as the major energy source for the adult brain.

Expression of genes involved in DNA replication, chromosome condensation and segregation is decreased at P13 versus P5, as well as that of cyclin-dependent protein kinase inhibitors that regulate the cell cycle. This pattern of changes is consistent with the known stages of brain development where rapid growth and differentiation end in the first postnatal week. Transcripts of genes for apoptotic mitochondrial changes are reduced at P13, which may indicate that tissue remodeling is slowing down. Similar changes in gene expression have been reported for other brain regions, including cerebellum [43], hippocampus [44], and cerebrum [45]. A novel finding, not previously identified in other brain regions, is the reduced expression of four genes encoding nuclear porin structures, nucleoporins *Nup107*, *Nup210*, *Nup43 and Nup205*, which may reflect a change in nuclear transport during this period of development.

### Lack of Detection of Gene Expression Differences between WT and *Snord116del* Hypothalami

In comparison with previous PWS mouse models, the *Snord116del* mouse has the smallest deletion and reproduces several major aspects of the human PWS phenotype, such as neonatal growth retardation, adult hyperphagia and hyperghrelinemia, elevated anxiety and motor learning deficiency [12]. To probe into the underlying physiology of the defect in *Snord116del* mice and in search for the effector genes and potential molecular target(s) for the *Snord116* C/D box snoRNA cluster, we compared the hypothalamic gene expression patterns of the *Snord116del* mice and wild-type littermates at P5, before and after maternal deprivation, and at P13. We detected no reliable differences between the genotypes.

Results of previous gene expression microarray analyses of tissues from human PWS individuals or mouse models had been uninformative or inconsistent. A study of PWS lymphoblastoid cells, including four with paternal 15q11–q13 deletion and three with maternal UPD, reported changes in the expression of multiple genes compared to controls [46]. These changes remain to be validated in human brain tissues. Microarray analysis had also been performed on two mouse models with large deletions encompassing the PWS critical region. In a study of whole brains of a transgene-induced 6.8 Mb deletion mouse model at postnatal day 1, no consistent changes were observed [47]. In contrast, dramatic increases in *Pomc1* and melanocortin receptor *Mc5r* expression were reported at postnatal day 0 in brains of a PWS mouse model with an imprinting center deletion which affects gene expression in a multi-megabase domain [48]. As these changes were not observed in a transgene-induced 6.8 Mb deletion mouse [47] nor in our current study, they may be specific to the mutation engineered in the IC-deletion mouse model instead of reflecting changes relevent to PWS phenotypes.

Our inability to demonstrate significant differences between *Snord116del* mice and wild-type littermates at the time points and conditions studied could be due to one or more of the following reasons. First, the *Snord116* deletion may affect gene expression only in a distinct population of cells or hypothalamic sub-regions, and the isolated hypothalami, although significantly smaller than whole brain (1/20 in weight), are still a crude mixture of multiple cell types. Second, relevant changes in gene expression may be small in magnitude and below the detection limit of the methods used. Third, *Snord116del* mice may have a primary defect in another brain region. A good candidate is the hindbrain dorsal vagal complex (DVC), to which neural projections descending from hypothalamus connect. The DVC has been implicated in the regulation of neonatal milk intake [49], as well as adult feeding behavior and visceral responses [50].

The function and molecular target(s) of *Snord116* are yet unknown. Canonical C/D box snoRNAs that have target sequences in rRNA, snRNA and tRNA, function to guide 2′-O-methylation of the ribose group of the nucleotide that base-pairs with the fifth nucleotide upstream of the D box of the snoRNA. *Snord116* has no sequence similarity with these RNA species. Despite the localization of snoRNAs in the nucleolus, their ability to modify mRNA has been suggested by reports that another imprinted snoRNA, *Snord115*, affects alternative splicing and editing of serotonin receptor *5HT2C* pre-mRNA [51], [52]. The future study of *Snord116del* mice may benefit from tiling exon arrays targeted to detect alternative splicing, as well as from examining isolated nuclei of the hypothalamus and additional brain regions, such as the dorsal vagal complex of hindbrain, to uncover the molecular mechanism that lead to neonatal failure to thrive and subsequent hyperphagia.

## Materials and Methods

### Mouse Breeding, Genotyping and Dissection

All animals were housed in a facility with light-on period from 7 am–7 pm with a room temperature of 23–25°C. *Snord116del* males (B6(Cg)-Snord116<tm1.1Uta>/J) [12] were mated to C57BL/6J females, and the pups were used to obtain hypothalamic RNA. For fasting studies, the dams were removed from the home cages of P5 pups at 9 am, and the pups in the same litter (5–9 pups) were left together in the home cage. At P13, the *Snord116del* pups are ∼40% smaller than the wild-type littermates and appear malnourished, therefore, they were not subjected to the stress of fasting. P5 and P13 pups were sacrificed between 3 and 4 pm by decapitation, and the whole brains were isolated within 5 min after decapitation and placed in >10× volume of cold RNAlater solution (Ambion) on ice with gentle swirling. After soaking overnight, a 3 mm thick section anterior to the ventral junction of the forebrain and cerebellum, which extend 0.5 mm beyond optic chiasma anteriorly, was dissected out using a brain slicer with 1mm spacing, and a hemicircular piece centered around the 3^rd^ ventricle, weighing on average 10–12 mg, was dissected under the microscope by using a micropunch with a 3mm diameter, and was used as hypothalamic sample. Based on comparisons with an adult mouse brain atlas [53] the isolated brain sections should contain all the hypothalamic nuclei and a small portion of the substantia nigra in the lateral posterior region, and should exclude thalamic nuclei. All experimental procedures were approved by the Stanford University Animal Care and Use Committee, and NIH Guidelines were followed.

### Extraction and Pooling of RNA

RNA was isolated using Trizol (Invitrogen) according to manufacturer's instruction. Subsequently, total RNA was treated with 10 units of RNase-free DNase (Ambion) in 200 µl solution at 37°C for 30 min, extracted with phenol-chloroform, ethanol precipitated, and dissolved in 30 µl DEPC-treated ddH_2_O. RNA integrity was evaluated by agarose gel electrophoresis demonstrating that the ribosomal RNA bands were intact. RNA concentration was determined by spectrophotometric measurement of OD260/280 (ratio>1.8). We obtained about 10 µg of DNase-treated total RNA for each 10 mg hypothalamic sample. For gene expression microarray analysis, we used pooled RNA to sample a larger number of individuals and to reduce the effect of individual variation. We were also concerned about litter to litter variation, which was determined to be an important factor in our previous study of a mouse model for Rett syndrome [54]. For each age and condition, the WT and deletion pools are from the same litter, and we chose litters with similar numbers of WT and deletion mice. Only for pools 11 and 12 individuals from three litters were combined. Equal amounts of RNA from individual hypothalamic samples were pooled (Table 2).

**Table 2:** Pooling scheme of hypothalamic samples for microarray analyses.

Pool#	ID	Condition	Genotype	Litter#(size), # of pups in each pool (sex)
1	AW	P5	WT	Lt1(10), 5 WT pups (5M)
2	AD	P5	Del	Lt1(10), 5 Del pups (2M, 3 F)
3	BW	P5-Fast	WT	Lt2(8), 4 WT pups (4M)
4	BD	P5-Fast	Del	Lt2(8), 4 Del pups (4 F)
5	CW	P5	WT	Lt3(7), 3 WT pups (3 F)
6	CD	P5	Del	Lt3(7), 4 Del pups (3 M, 1 F)
7	DW	P5-Fast	WT	Lt4(8), 5 WT pups (4 M, 1 F)
8	DD	P5-Fast	Del	Lt4(8), 3 Del pups (3 F)
9	EW	P5	WT	Lt5(6), 2 WT pups (1 M, 1 unknown)
10	ED	P5	Del	Lt5(6), 2 Del p5ups (1 M, 1 F)
11	FW	P5	WT	Lt6(6), 1 WT (1 F); Lt7(5), 4 WT (2 M, 2F); Lt8(4), 3 WT pups (3F)
12	FD	P5	Del	Lt6(6), 5 Del (4 F, 1 unknown); Lt7(5), 1 Del (1M); Lt8(4), 1 Del pups (1F)
13	GW	P13	WT	Lt9(9), 5 WT pups (3M, 2F)
14	GD	P13	Del	Lt9(9), 4 Del pups (2M, 1F, 1 unknown)
15	HW	P13	WT	Lt10(8), 3 WT pups (1M, 2F)
16	HD	P13	Del	Lt10(8), 5 Del pups (2M, 3F)

### Illumina Expression Array Hybridization and Data Analysis

We used the Illumina MouseRef-8 v1.1 Expression BeadChips (Illumina, Cat. no. BD-26–201) for gene expression profiling. These chips cover over 24,000 well-annotated mouse RefSeq transcripts, and each contains eight slots for analyzing eight samples. We used two Beadchips to analyze the 16 RNA pools described above. We generated biotinylated, amplified RNA from pooled hypothalamic RNA by using the TotalPrep RNA Amplification Kit (Ambion, Cat. no. IL1791-1) following the manufacturer's instructions. Briefly, we synthesized first strand cDNA by using 250 ng total RNA. After second strand cDNA synthesis and cDNA purification, *in vitro* transcription overnight generated biotinylated amplified RNA, which was purified according to instructions and eluted in 100 µl nuclease-free water. A total of 850 ng of RNA was hybridized to the chips. Chips were read by the Illumina BeadStation 500× and the data were processed by the BeadScan software.

The raw data were processed by the Illumina BeadStudio Data Analysis Software, which generated the averaged intensity signals across ∼30 beads for each transcript. We imported the intensity data for all 24,600 transcripts across 16 samples into R [55], log transformed with base of 2, and quantile normalized the data to generate an equal intensity distribution across the 16 samples [56]. We re-aligned the >24,600 Illumina probes to the mouse genome and found that 20,328 probes are of high mapping quality. The mapped probes are available from the authors upon request. The 20,328 probes map to 15,928 unique HUGO gene symbols. We averaged the normalized expression values of probes corresponding to the same symbols. Using the resulting dataset of 15,928 symbols, we calculated two-group t tests to identify transcripts showing differential expression between the following conditions, as illustrated in Fig. 1: Comparison 1: P5 WT vs. *Snord116del* non-fasted mice; Comparison 2: P5 WT vs. *Snord116del* fasted mice; comparison 3: P5 non-fasted vs. fasted WT mice; comparison 4: P5 non-fasted vs. fasted *Snord116del* mice; comparison 5: P13 WT vs. P13 *Snord116del* mice; comparison 6: P5 WT vs. P13 WT mice; comparison 7: P5 *Snord116del* vs. P13 *Snord116del* mice. For each of the comparisons, we recorded the t scores, *P*-values and fold-changes (expressed in log scale). These data were further filtered, with genes showing 2^0.3^-fold difference and P≤0.05 considered significantly different. The raw data and normalized data for the 16 samples were MIAME compliant and have been deposited in NCBI's Gene Expression Omnibus [57]. They are accessible through GEO Series accession number GSE14984 (http://www.ncbi.nlm.nih.gov/geo/query/acc.cgi?acc=GSE14984).

### Real-Time Quantitative RT-PCR Validation

Hypothalamic RNA samples from each individual mouse were reverse transcribed with M-MLV reverse transcriptase (NEB). For each 20µl reaction, 1µg of RNA was used, and RT reactions were diluted 1∶20, and 5 µl was used in each real-time PCR reaction with SYBR green methods (Applied Biosystems) on an ABI-5700 instrument. Each sample was tested in triplicate. For most of the genes analyzed by real-time RT-PCR, the primers sequences were derived from PrimerBank [58]. The primer sequences are listed in Supplementary Table S5. The expression of each gene was normalized to that of ribosomal protein S28 in the same sample. The average level of WT samples without fasting was defined as 1.00 when calculating the ratio of the expression levels of other test conditions. For pair-wise comparison, Student's t test was used. For comparison of the P5 WT and deletion hypothalamic RNA samples with or without 6 hr fasting, StatView software was used to perform ANOVA analysis, and the *p* values of Fisher's test results are reported.

### Gene Ontology Analysis

We used Gene Ontology Tree Machine of Vanderbilt University (http://bioinfo.vanderbilt.edu/gotm) **for** gene ontology analysis. We submitted the list of genes with fold of change >2^0.3^, t>4 and false discovery rate <0.05 for P5–P13 comparisons. From the highly branched Gene Ontology tree, we report the lowest level of branches with *p* values less than 0.05. And if a few categories shared most genes, we only report one representative group.

## Supporting Information

Figure S1 Body weight of pups used for hypothalamic gene expression study. Horizontal bar, average weight. In parentheses, total number of pups used in each group. W, wild-type; D, Snord116del.
(0.19 MB TIF)

Table S1 Comparison 3. P5 wild type non-starved vs. starved (1-AW, 5-CW, 9-EW, 11-FW) vs. (3-BW, 7-DW).
(0.25 MB DOC)

Table S2 Comparison 4. P5 Snord116del starved vs non-starved (2-AD, 6-CD, 10-ED, 12-FD) vs. (4-BD, 8-DD).
(0.18 MB DOC)

Table S3 Comparison 6. P5 wild type vs. P13 wild type (1-AW, 5-CW, 9-EW, 11-FW) vs.(13-GW. 15-HW)
(0.61 MB DOC)

Table S4 Comparison 7. P5 Snord116del vs. P13 Snord116del (2-AD, 6-CD, 10-ED, 12-FD) vs. (14-GD, 16-GD).
(0.46 MB DOC)

Table S5 Primers for real-time RT-PCR validation.
(0.06 MB DOC)

## References

[1] Wadhwa PD, Buss C, Entringer S, Swanson JM (2009). Developmental origins of health and disease: brief history of the approach and current focus on epigenetic mechanisms.. Semin Reprod Med.

[2] Holm VA, Cassidy SB, Butler MG, Hanchett JM, Greenswag LR (1993). Prader-Willi syndrome: consensus diagnostic criteria.. Pediatrics.

[3] White SM, Thompson EM, Kidd A, Savarirayan R, Turner A (2004). Growth, behavior, and clinical findings in 27 patients with Kabuki (Niikawa-Kuroki) syndrome.. Am J Med Genet A.

[4] Gecz J, Turner G, Nelson J, Partington M (2006). The Borjeson-Forssman-Lehman syndrome (BFLS, MIM #301900).. Eur J Hum Genet.

[5] Cassidy SB, McCandless SE, Cassidy SB, Allanson JE (2005). Prader-Willi Syndrome.. Management of Genetic Syndromes. 2nd ed.

[6] Nieuwenhuizen AG, Rutters F (2008). The hypothalamic-pituitary-adrenal-axis in the regulation of energy balance.. Physiol Behav.

[7] Holland A, Whittington J, Hinton E (2003). The paradox of Prader-Willi syndrome: a genetic model of starvation.. Lancet.

[8] Morton GJ, Cummings DE, Baskin DG, Barsh GS, Schwartz MW (2006). Central nervous system control of food intake and body weight.. Nature.

[9] Li JY, Lescure PA, Misek DE, Lai YM, Chai BX (2002). Food deprivation-induced expression of minoxidil sulfotransferase in the hypothalamus uncovered by microarray analysis.. J Biol Chem.

[10] Schmidt MV, Levine S, Alam S, Harbich D, Sterlemann V (2006). Metabolic signals modulate hypothalamic-pituitary-adrenal axis activation during maternal separation of the neonatal mouse.. J Neuroendocrinol.

[11] de Smith AJ, Purmann C, Walters RG, Ellis RJ, Holder SE (2009). A deletion of the HBII-85 class of small nucleolar RNAs (snoRNAs) is associated with hyperphagia, obesity and hypogonadism.. Hum Mol Genet.

[12] Ding F, Li HH, Zhang S, Solomon NM, Camper SA (2008). SnoRNA Snord116 (Pwcr1/MBII-85) deletion causes growth deficiency and hyperphagia in mice.. PLoS ONE.

[13] DelParigi A, Tschop M, Heiman ML, Salbe AD, Vozarova B (2002). High circulating ghrelin: a potential cause for hyperphagia and obesity in prader-willi syndrome.. J Clin Endocrinol Metab.

[14] Cummings DE, Clement K, Purnell JQ, Vaisse C, Foster KE (2002). Elevated plasma ghrelin levels in Prader Willi syndrome.. Nat Med.

[15] Lau C, Henning SJ (1984). Regulation of milk ingestion in the infant rat.. Physiol Behav.

[16] Xu AW, Ste-Marie L, Kaelin CB, Barsh GS (2007). Inactivation of signal transducer and activator of transcription 3 in proopiomelanocortin (Pomc) neurons causes decreased pomc expression, mild obesity, and defects in compensatory refeeding.. Endocrinology.

[17] Schmidt M, Enthoven L, van Woezik JH, Levine S, de Kloet ER (2004). The dynamics of the hypothalamic-pituitary-adrenal axis during maternal deprivation.. J Neuroendocrinol.

[18] Schmidt MV, Liebl C, Sterlemann V, Ganea K, Hartmann J (2008). Neuropeptide Y mediates the initial hypothalamic-pituitary-adrenal response to maternal separation in the neonatal mouse.. J Endocrinol.

[19] Cirulli F, Gottlieb S, Arosenfeld P, Levine S (1992). Maternal factors regulate stress responsiveness in the neonatal rat.. Psychobiology.

[20] Malik SS, Fewell JE (2003). Thermoregulation in rats during early postnatal maturation: importance of nitric oxide.. Am J Physiol Regul Integr Comp Physiol.

[21] Bertin R, Mouroux I, De Marco F, Portet R (1990). Norepinephrine turnover in brown adipose tissue of young rats: effects of rearing temperature.. Am J Physiol.

[22] Hofer MA, Brunelli SA, Shair HN (1993). The effects of 24-hr maternal separation and of litter-size reduction on the isolation-distress response of 12-day-old rat pups.. Dev Psychobiol.

[23] Barker DD, Wang C, Moore J, Dickinson LK, Lehmann R (1992). Pumilio is essential for function but not for distribution of the Drosophila abdominal determinant Nanos.. Genes Dev.

[24] Tautz D, Pfeifle C (1989). A non-radioactive in situ hybridization method for the localization of specific RNAs in Drosophila embryos reveals translational control of the segmentation gene hunchback.. Chromosoma.

[25] Tsuda M, Sasaoka Y, Kiso M, Abe K, Haraguchi S (2003). Conserved role of nanos proteins in germ cell development.. Science.

[26] Tsuda M, Kiso M, Saga Y (2006). Implication of nanos2-3′UTR in the expression and function of nanos2.. Mech Dev.

[27] Suzuki A, Tsuda M, Saga Y (2007). Functional redundancy among Nanos proteins and a distinct role of Nanos2 during male germ cell development.. Development.

[28] Roche TE, Hiromasa Y (2007). Pyruvate dehydrogenase kinase regulatory mechanisms and inhibition in treating diabetes, heart ischemia, and cancer.. Cell Mol Life Sci.

[29] Spriet LL, Tunstall RJ, Watt MJ, Mehan KA, Hargreaves M (2004). Pyruvate dehydrogenase activation and kinase expression in human skeletal muscle during fasting.. J Appl Physiol.

[30] Vannucci RC, Vannucci SJ (2000). Glucose metabolism in the developing brain.. Semin Perinatol.

[31] Xu Q, Wang Y, Dabdoub A, Smallwood PM, Williams J (2004). Vascular development in the retina and inner ear: control by Norrin and Frizzled-4, a high-affinity ligand-receptor pair.. Cell.

[32] Luhmann UF, Neidhardt J, Kloeckener-Gruissem B, Schafer NF, Glaus E (2008). Vascular changes in the cerebellum of Norrin/Ndph knockout mice correlate with high expression of Norrin and Frizzled-4.. Eur J Neurosci.

[33] Cummins PM, O'Connor B (1998). Pyroglutamyl peptidase: an overview of the three known enzymatic forms.. Biochim Biophys Acta.

[34] Perlman JH, Colson AO, Jain R, Czyzewski B, Cohen LA (1997). Role of the extracellular loops of the thyrotropin-releasing hormone receptor: evidence for an initial interaction with thyrotropin-releasing hormone.. Biochemistry.

[35] Larrinaga G, Callado LF, Agirregoitia N, Varona A, Gil J (2005). Subcellular distribution of membrane-bound aminopeptidases in the human and rat brain.. Neurosci Lett.

[36] Nikolaev AY, Papanikolaou NA, Li M, Qin J, Gu W (2004). Identification of a novel BRMS1-homologue protein p40 as a component of the mSin3A/p33(ING1b)/HDAC1 deacetylase complex.. Biochem Biophys Res Commun.

[37] Meehan WJ, Welch DR (2003). Breast cancer metastasis suppressor 1: update.. Clin Exp Metastasis.

[38] Hurst DR, Xie Y, Vaidya KS, Mehta A, Moore BP (2008). Alterations of BRMS1-ARID4A interaction modify gene expression but still suppress metastasis in human breast cancer cells.. J Biol Chem.

[39] Hedley BD, Vaidya KS, Phadke P, MacKenzie L, Dales DW (2008). BRMS1 suppresses breast cancer metastasis in multiple experimental models of metastasis by reducing solitary cell survival and inhibiting growth initiation.. Clin Exp Metastasis.

[40] Bonifacino JS, Hurley JH (2008). Retromer.. Curr Opin Cell Biol.

[41] Gullapalli A, Wolfe BL, Griffin CT, Magnuson T, Trejo J (2006). An essential role for SNX1 in lysosomal sorting of protease-activated receptor-1: evidence for retromer-, Hrs-, and Tsg101-independent functions of sorting nexins.. Mol Biol Cell.

[42] Bouret SG, Simerly RB (2004). Minireview: Leptin and development of hypothalamic feeding circuits.. Endocrinology.

[43] Miki R, Kadota K, Bono H, Mizuno Y, Tomaru Y (2001). Delineating developmental and metabolic pathways in vivo by expression profiling using the RIKEN set of 18,816 full-length enriched mouse cDNA arrays.. Proc Natl Acad Sci U S A.

[44] Mody M, Cao Y, Cui Z, Tay KY, Shyong A (2001). Genome-wide gene expression profiles of the developing mouse hippocampus.. Proc Natl Acad Sci U S A.

[45] Lim CR, Fukakusa A, Matsubara K (2004). Gene expression profiling of mouse postnatal cerebellar development using cDNA microarrays.. Gene.

[46] Bittel DC, Kibiryeva N, McNulty SG, Driscoll DJ, Butler MG (2007). Whole genome microarray analysis of gene expression in an imprinting center deletion mouse model of Prader-Willi syndrome.. Am J Med Genet A.

[47] Stefan M, Portis T, Longnecker R, Nicholls RD (2005). A nonimprinted Prader-Willi Syndrome (PWS)-region gene regulates a different chromosomal domain in trans but the imprinted pws loci do not alter genome-wide mRNA levels.. Genomics.

[48] Bittel DC, Kibiryeva N, Sell SM, Strong TV, Butler MG (2007). Whole genome microarray analysis of gene expression in Prader-Willi syndrome.. Am J Med Genet A.

[49] Rinaman L (2006). Ontogeny of hypothalamic-hindbrain feeding control circuits.. Dev Psychobiol.

[50] Grill HJ, Schwartz MW, Kaplan JM, Foxhall JS, Breininger J (2002). Evidence that the caudal brainstem is a target for the inhibitory effect of leptin on food intake.. Endocrinology.

[51] Kishore S, Stamm S (2006). The snoRNA HBII-52 regulates alternative splicing of the serotonin receptor 2C.. Science.

[52] Vitali P, Basyuk E, Le Meur E, Bertrand E, Muscatelli F (2005). ADAR2-mediated editing of RNA substrates in the nucleolus is inhibited by C/D small nucleolar RNAs.. J Cell Biol.

[53] Franklin K, Paxinos G (1997). The Mouse Brain in stereotaxic coordinates.

[54] Jordan C, Li HH, Kwan HC, Francke U (2007). Cerebellar gene expression profiles of mouse models for Rett syndrome reveal novel MeCP2 targets.. BMC Med Genet.

[55] R CDT (2008). R: A language and environment for statistical computing.

[56] Bolstad BM, Irizarry RA, Astrand M, Speed TP (2003). A comparison of normalization methods for high density oligonucleotide array data based on variance and bias.. Bioinformatics.

[57] Edgar R, Domrachev M, Lash AE (2002). Gene Expression Omnibus: NCBI gene expression and hybridization array data repository.. Nucleic Acids Res.

[58] Wang X, Seed B (2003). A PCR primer bank for quantitative gene expression analysis.. Nucleic Acids Res.

