# Supplementary figures and images for: Neonatal Maternal Deprivation Response and Developmental Changes in Gene Expression Revealed by Hypothalamic Gene Expression Profiling in Mice
Source: PLoS One. 2010 Feb 24;5(2):e9402. doi: 10.1371/journal.pone.0009402 (PMC2827556; doi:10.1371/journal.pone.0009402)

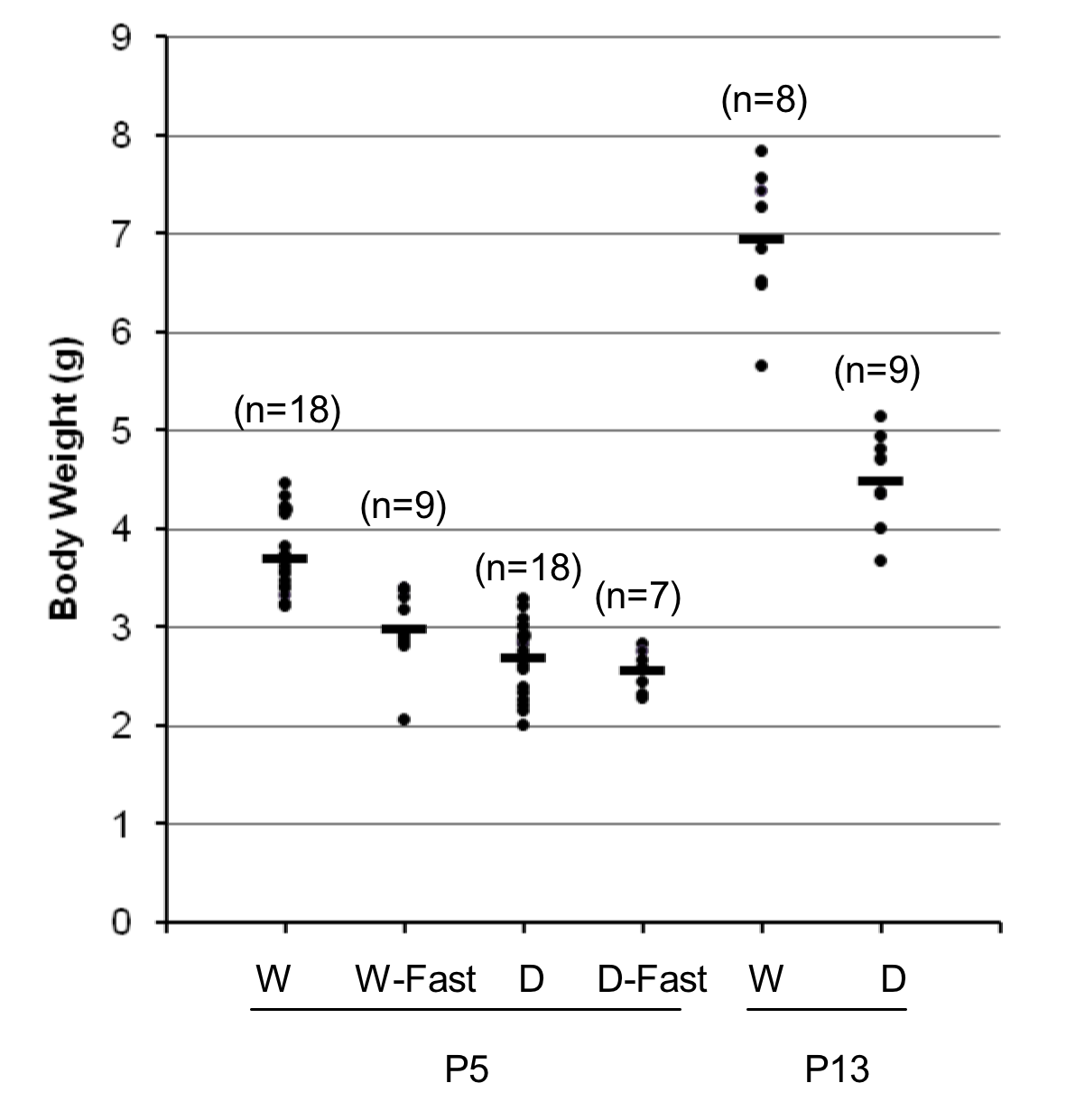

Supplement: Figure S1 — Body weight of pups used for hypothalamic gene expression study. Horizontal bar, average weight. In parentheses, total number of pups used in each group. W, wild-type; D, Snord116del. (0.19 MB TIF) [file pone.0009402.s001.tif]
