# Supplementary material for: Neonatal Maternal Deprivation Response and Developmental Changes in Gene Expression Revealed by Hypothalamic Gene Expression Profiling in Mice
Source: PLoS One. 2010 Feb 24;5(2):e9402. doi: 10.1371/journal.pone.0009402 (PMC2827556; doi:10.1371/journal.pone.0009402)
Supplement: Table S1 — Comparison 3. P5 wild type non-starved vs. starved (1-AW, 5-CW, 9-EW, 11-FW) vs. (3-BW, 7-DW). (0.25 MB DOC) [file pone.0009402.s002.doc]

Table S1. Comparison 3. P5 wild type non-starved vs. starved (1-AW, 5-CW, 9-EW, 11-FW)

vs. (3-BW, 7-DW). t-value is the ratio of the estimated change divided by the standard error.

BH-FDR: Bonferroni Hochberg false discovery rate. High-lighted are genes validated by

quantitative RT-PCR as shown in table 1.

| Comp3 | Gene Symbol | t -value | Fold_change | p-value | BH-FDR |
| --- | --- | --- | --- | --- | --- |
| 1 | Arrdc4 | 5.89 | 1.18 | 0.0042 | 0.299 |
| 2 | 9830001H06Rik | 6.94 | 0.78 | 0.0023 | 0.275 |
| 3 | **Nanos2** | 9.01 | 0.68 | 0.0008 | 0.224 |
| 4 | Mvp | 9.79 | 0.62 | 0.0006 | 0.217 |
| 5 | **Pdk4** | 14.11 | 0.59 | 0.0001 | 0.214 |
| 6 | Zfp281 | 6.39 | 0.57 | 0.0031 | 0.291 |
| 7 | Olfr167 | 4.66 | 0.56 | 0.0096 | 0.338 |
| 8 | Prss27 | 5.11 | 0.56 | 0.0069 | 0.322 |
| 9 | 2700045P11Rik | 5.53 | 0.53 | 0.0052 | 0.309 |
| 10 | Drd4 | 4.54 | 0.52 | 0.0105 | 0.349 |
| 11 | Mical3 | 6.82 | 0.50 | 0.0024 | 0.277 |
| 12 | Slc36a4 | 6.72 | 0.50 | 0.0026 | 0.284 |
| 13 | Prr7 | 6.23 | 0.50 | 0.0034 | 0.298 |
| 14 | Fus | 5.85 | 0.48 | 0.0043 | 0.299 |
| 15 | Agrp | 4.09 | 0.48 | 0.0150 | 0.362 |
| 16 | Ppif | 4.53 | 0.47 | 0.0106 | 0.349 |
| 17 | Itpka | 10.37 | 0.46 | 0.0005 | 0.216 |
| 18 | Snx30 | 15.71 | 0.46 | 0.0001 | 0.214 |
| 19 | BC005764 | 10.76 | 0.46 | 0.0004 | 0.214 |
| 20 | Pou3f1 | 5.15 | 0.45 | 0.0068 | 0.322 |
| 21 | Ugt3a2 | 4.05 | 0.45 | 0.0155 | 0.364 |
| 22 | Crsp7 | 8.70 | 0.45 | 0.0010 | 0.224 |
| 23 | Ahnak | 9.33 | 0.44 | 0.0007 | 0.217 |
| 24 | Pde1a | 21.74 | 0.44 | 0.0000 | 0.211 |
| 25 | Nipsnap1 | 5.03 | 0.44 | 0.0073 | 0.322 |
| 26 | Arf6 | 4.24 | 0.43 | 0.0133 | 0.358 |
| 27 | Gtf2f2 | 10.30 | 0.43 | 0.0005 | 0.216 |
| 28 | Cyp2j5 | 7.80 | 0.43 | 0.0015 | 0.264 |
| 29 | 4833401D15Rik | 4.82 | 0.42 | 0.0085 | 0.331 |
| 30 | Frat1 | 6.68 | 0.42 | 0.0026 | 0.284 |
| 31 | Odz4 | 9.95 | 0.42 | 0.0006 | 0.217 |
| 32 | 0610009K11Rik | 4.67 | 0.42 | 0.0095 | 0.338 |
| 33 | Sp5 | 4.26 | 0.42 | 0.0131 | 0.358 |
| 34 | Rfc3 | 6.01 | 0.42 | 0.0039 | 0.299 |
| 35 | Gmeb2 | 5.96 | 0.42 | 0.0040 | 0.299 |
| 36 | Ccdc71 | 4.61 | 0.42 | 0.0099 | 0.343 |
| 37 | Exosc4 | 4.11 | 0.41 | 0.0147 | 0.362 |
| 38 | Mt2 | 4.75 | 0.41 | 0.0090 | 0.336 |
| 39 | 4632419K20Rik | 9.34 | 0.41 | 0.0007 | 0.217 |
| 40 | Bbs1 | 5.10 | 0.41 | 0.0070 | 0.322 |
| 41 | 4930583H14Rik | 6.30 | 0.41 | 0.0032 | 0.294 |
| 42 | Prr8 | 4.18 | 0.41 | 0.0139 | 0.362 |
| 43 | 6620401K05Rik | 5.05 | 0.41 | 0.0073 | 0.322 |
| 44 | Pde10a | 4.44 | 0.40 | 0.0114 | 0.353 |
| 45 | Ctnnbl1 | 5.08 | 0.40 | 0.0071 | 0.322 |
| 46 | Klf5 | 4.10 | 0.40 | 0.0149 | 0.362 |
| 47 | Rgag4 | 4.02 | 0.40 | 0.0158 | 0.364 |
| 48 | Kpna4 | 4.04 | 0.40 | 0.0156 | 0.364 |
| 49 | D16Ertd472e | 4.80 | 0.40 | 0.0087 | 0.333 |
| 50 | Rasal1 | 10.22 | 0.40 | 0.0005 | 0.217 |
| 51 | Strn4 | 8.64 | 0.38 | 0.0010 | 0.224 |
| 52 | Mllt7 | 5.27 | 0.37 | 0.0062 | 0.321 |
| 53 | Hivep1 | 6.15 | 0.37 | 0.0035 | 0.298 |
| 54 | Rab11fip5 | 10.71 | 0.37 | 0.0004 | 0.214 |
| 55 | Olfr1214 | 4.18 | 0.36 | 0.0139 | 0.362 |
| 56 | Ankrd50 | 12.76 | 0.36 | 0.0002 | 0.214 |
| 57 | Kcnk12 | 4.96 | 0.36 | 0.0077 | 0.322 |
| 58 | 6330527O06Rik | 4.16 | 0.36 | 0.0142 | 0.362 |
| 59 | Hmgcr | 4.02 | 0.36 | 0.0159 | 0.364 |
| 60 | Prap1 | 4.29 | 0.36 | 0.0128 | 0.358 |
| 61 | Slc27a4 | 4.46 | 0.36 | 0.0112 | 0.353 |
| 62 | 1700095G12Rik | 6.37 | 0.35 | 0.0031 | 0.291 |
| 63 | Igbp1b | 4.46 | 0.35 | 0.0112 | 0.353 |
| 64 | R3hcc1 | 4.72 | 0.35 | 0.0092 | 0.338 |
| 65 | Zfp719 | 5.05 | 0.35 | 0.0072 | 0.322 |
| 66 | Eefsec | 5.54 | 0.35 | 0.0052 | 0.309 |
| 67 | Gpr135 | 4.45 | 0.35 | 0.0112 | 0.353 |
| 68 | Nfkbib | 4.76 | 0.35 | 0.0089 | 0.335 |
| 69 | Grin2c | 6.93 | 0.35 | 0.0023 | 0.275 |
| 70 | Spink3 | 6.07 | 0.35 | 0.0037 | 0.299 |
| 71 | Htatip | 9.45 | 0.34 | 0.0007 | 0.217 |
| 72 | Olfr90 | 5.01 | 0.34 | 0.0074 | 0.322 |
| 73 | Rcn1 | 5.23 | 0.34 | 0.0064 | 0.322 |
| 74 | Casp7 | 4.77 | 0.34 | 0.0088 | 0.334 |
| 75 | Cpne5 | 4.58 | 0.34 | 0.0102 | 0.347 |
| 76 | Ptcra | 4.09 | 0.34 | 0.0149 | 0.362 |
| 77 | Zc3hav1 | 6.20 | 0.34 | 0.0034 | 0.298 |
| 78 | Gk5 | 5.47 | 0.34 | 0.0054 | 0.311 |
| 79 | Taf5l | 6.03 | 0.34 | 0.0038 | 0.299 |
| 80 | 2410016O06Rik | 4.38 | 0.33 | 0.0119 | 0.354 |
| 81 | Slc7a9 | 5.26 | 0.33 | 0.0062 | 0.321 |
| 82 | Ptprcap | 8.08 | 0.33 | 0.0013 | 0.246 |
| 83 | Uvrag | 6.32 | 0.32 | 0.0032 | 0.293 |
| 84 | Cd163 | 6.18 | 0.32 | 0.0035 | 0.298 |
| 85 | Zdhhc25 | 7.25 | 0.32 | 0.0019 | 0.273 |
| 86 | Bcl11a | 7.66 | 0.32 | 0.0016 | 0.273 |
| 87 | Per1 | 9.32 | 0.32 | 0.0007 | 0.217 |
| 88 | Sult1c2 | 5.11 | 0.32 | 0.0069 | 0.322 |
| 89 | Otos | 4.11 | 0.32 | 0.0147 | 0.362 |
| 90 | 1700054N08Rik | 6.52 | 0.31 | 0.0028 | 0.287 |
| 91 | Zfp536 | 4.17 | 0.31 | 0.0140 | 0.362 |
| 92 | Smn1 | 4.32 | 0.31 | 0.0125 | 0.357 |
| 93 | Amn | 5.18 | 0.31 | 0.0066 | 0.322 |
| 94 | Cecr6 | 7.42 | 0.31 | 0.0018 | 0.273 |
| 95 | Col14a1 | 4.46 | 0.31 | 0.0111 | 0.353 |
| 96 | Utp15 | 5.95 | 0.31 | 0.0040 | 0.299 |
| 97 | Ap3m1 | 7.48 | 0.31 | 0.0017 | 0.273 |
| 98 | Vps37b | 9.99 | 0.31 | 0.0006 | 0.217 |
| 99 | Btbd7 | 4.23 | 0.31 | 0.0133 | 0.358 |
| 100 | Magi1 | 11.74 | 0.31 | 0.0003 | 0.214 |
| 101 | Lsp1 | 4.88 | 0.31 | 0.0082 | 0.331 |
| 102 | Fbxo43 | 4.17 | 0.30 | 0.0141 | 0.362 |
| 103 | Wtip | 4.46 | 0.30 | 0.0111 | 0.353 |
| 104 | 2610524H06Rik | 4.11 | 0.30 | 0.0147 | 0.362 |
| 105 | V1rc24 | 5.36 | 0.30 | 0.0059 | 0.319 |
| 106 | 2410066E13Rik | 8.64 | 0.30 | 0.0010 | 0.224 |

| Comp3 | Gene Symbol | t-value | Fold_change | p-value | BH_FDR |
| --- | --- | --- | --- | --- | --- |
| 1 | Dusp18 | -11.92 | -0.76 | 0.0003 | 0.214 |
| 2 | Nkx2-2 | -8.53 | -0.69 | 0.0010 | 0.228 |
| 3 | Lime1 | -6.77 | -0.66 | 0.0025 | 0.282 |
| 4 | Ing2 | -4.99 | -0.63 | 0.0075 | 0.322 |
| 5 | **Snx1** | -6.07 | -0.62 | 0.0037 | 0.299 |
| 6 | Slc11a2 | -11.54 | -0.62 | 0.0003 | 0.214 |
| 7 | 2410002O22Rik | -12.65 | -0.62 | 0.0002 | 0.214 |
| 8 | **Pgpep1** | -6.71 | -0.60 | 0.0026 | 0.284 |
| 9 | Slc22a4 | -6.18 | -0.59 | 0.0035 | 0.298 |
| 10 | Serpini1 | -4.64 | -0.58 | 0.0097 | 0.341 |
| 11 | Sh3bgrl2 | -4.01 | -0.58 | 0.0160 | 0.364 |
| 12 | Krcc1 | -5.09 | -0.56 | 0.0070 | 0.322 |
| 13 | Mitd1 | -6.89 | -0.54 | 0.0023 | 0.277 |
| 14 | Mgmt | -6.36 | -0.54 | 0.0031 | 0.291 |
| 15 | Cd59b | -4.82 | -0.52 | 0.0085 | 0.331 |
| 16 | Klhl29 | -5.37 | -0.50 | 0.0058 | 0.319 |
| 17 | Nek1 | -4.51 | -0.50 | 0.0107 | 0.349 |
| 18 | Pcdh20 | -4.69 | -0.50 | 0.0094 | 0.338 |
| 19 | Pcmtd2 | -4.89 | -0.49 | 0.0081 | 0.331 |
| 20 | Mtrf1l | -8.91 | -0.49 | 0.0009 | 0.224 |
| 21 | Agtr1a | -4.69 | -0.48 | 0.0094 | 0.338 |
| 22 | Mfap4 | -4.11 | -0.48 | 0.0148 | 0.362 |
| 23 | Abcg1 | -5.41 | -0.47 | 0.0057 | 0.317 |
| 24 | Pkd1 | -5.64 | -0.46 | 0.0049 | 0.299 |
| 25 | 9130227C08Rik | -12.81 | -0.45 | 0.0002 | 0.214 |
| 26 | Lima1 | -7.64 | -0.45 | 0.0016 | 0.273 |
| 27 | Ccm2 | -5.00 | -0.44 | 0.0075 | 0.322 |
| 28 | Hpcal4 | -8.43 | -0.44 | 0.0011 | 0.232 |
| 29 | Samd9l | -10.88 | -0.44 | 0.0004 | 0.214 |
| 30 | 2610301B20Rik | -5.05 | -0.43 | 0.0072 | 0.322 |
| 31 | **Brms1l** | -12.69 | -0.43 | 0.0002 | 0.214 |
| 32 | Fstl3 | -5.69 | -0.43 | 0.0047 | 0.299 |
| 33 | Mbnl2 | -4.38 | -0.43 | 0.0118 | 0.354 |
| 34 | Pglyrp1 | -4.17 | -0.43 | 0.0140 | 0.362 |
| 35 | Kctd9 | -12.85 | -0.43 | 0.0002 | 0.214 |
| 36 | Kdelc1 | -5.30 | -0.43 | 0.0061 | 0.319 |
| 37 | Gpr125 | -7.08 | -0.43 | 0.0021 | 0.275 |
| 38 | Ak3l1 | -7.13 | -0.43 | 0.0020 | 0.275 |
| 39 | 1190005P17Rik | -4.54 | -0.42 | 0.0105 | 0.349 |
| 40 | 2610044O15Rik | -5.20 | -0.42 | 0.0065 | 0.322 |
| 41 | Mrpl51 | -4.86 | -0.42 | 0.0083 | 0.331 |
| 42 | **Mett10d** | -6.49 | -0.41 | 0.0029 | 0.287 |
| 43 | Chordc1 | -4.80 | -0.41 | 0.0086 | 0.333 |
| 44 | Dtd1 | -6.13 | -0.41 | 0.0036 | 0.298 |
| 45 | Anxa5 | -5.98 | -0.40 | 0.0039 | 0.299 |
| 46 | Aqr | -4.32 | -0.40 | 0.0124 | 0.357 |
| 47 | Pcca | -4.29 | -0.40 | 0.0127 | 0.357 |
| 48 | Klf11 | -6.82 | -0.40 | 0.0024 | 0.277 |
| 49 | Itga6 | -5.06 | -0.39 | 0.0072 | 0.322 |
| 50 | **Ndph** | -10.76 | -0.39 | 0.0004 | 0.214 |
| 51 | Lims2 | -5.66 | -0.39 | 0.0048 | 0.299 |
| 52 | Car4 | -4.12 | -0.39 | 0.0147 | 0.362 |
| 53 | Manea | -5.66 | -0.39 | 0.0048 | 0.299 |
| 54 | Edd1 | -7.26 | -0.38 | 0.0019 | 0.273 |
| 55 | Tanc1 | -5.58 | -0.38 | 0.0051 | 0.305 |
| 56 | Hisppd2a | -4.38 | -0.38 | 0.0119 | 0.354 |
| 57 | Zfp111 | -6.50 | -0.38 | 0.0029 | 0.287 |
| 58 | Rexo4 | -6.60 | -0.38 | 0.0027 | 0.287 |
| 59 | Msc | -5.78 | -0.38 | 0.0044 | 0.299 |
| 60 | D19Ertd386e | -6.29 | -0.38 | 0.0033 | 0.294 |
| 61 | Prkag3 | -5.15 | -0.37 | 0.0067 | 0.322 |
| 62 | Hspa12b | -8.68 | -0.37 | 0.0010 | 0.224 |
| 63 | Kcne1l | -9.19 | -0.37 | 0.0008 | 0.224 |
| 64 | Ccdc66 | -4.69 | -0.36 | 0.0094 | 0.338 |
| 65 | Sdccag8 | -4.45 | -0.36 | 0.0112 | 0.353 |
| 66 | Ccdc51 | -4.22 | -0.36 | 0.0135 | 0.358 |
| 67 | Mfsd9 | -8.61 | -0.36 | 0.0010 | 0.224 |
| 68 | Papolg | -4.44 | -0.36 | 0.0113 | 0.353 |
| 69 | Gprc5b | -4.25 | -0.35 | 0.0131 | 0.358 |
| 70 | Sav1 | -5.14 | -0.35 | 0.0068 | 0.322 |
| 71 | Rps6ka4 | -6.05 | -0.35 | 0.0038 | 0.299 |
| 72 | Cept1 | -5.05 | -0.35 | 0.0072 | 0.322 |
| 73 | Tjap1 | -5.12 | -0.35 | 0.0069 | 0.322 |
| 74 | Herpud1 | -4.35 | -0.35 | 0.0121 | 0.354 |
| 75 | Golga1 | -8.97 | -0.34 | 0.0009 | 0.224 |
| 76 | Asb14 | -4.83 | -0.34 | 0.0085 | 0.331 |
| 77 | Cd300lb | -4.72 | -0.34 | 0.0092 | 0.338 |
| 78 | Tspan12 | -4.24 | -0.34 | 0.0133 | 0.358 |
| 79 | Pold3 | -5.48 | -0.33 | 0.0054 | 0.311 |
| 80 | Olfr1 | -4.22 | -0.33 | 0.0135 | 0.358 |
| 81 | 4933433C11Rik | -4.02 | -0.33 | 0.0158 | 0.364 |
| 82 | 4632428N05Rik | -4.37 | -0.33 | 0.0120 | 0.354 |
| 83 | Fastkd1 | -4.57 | -0.33 | 0.0103 | 0.348 |
| 84 | BB146404 | -4.32 | -0.33 | 0.0124 | 0.357 |
| 85 | Mrm1 | -4.89 | -0.33 | 0.0081 | 0.331 |
| 86 | Il16 | -4.26 | -0.33 | 0.0131 | 0.358 |
| 87 | BC004022 | -6.17 | -0.32 | 0.0035 | 0.298 |
| 88 | Shoc2 | -4.84 | -0.32 | 0.0084 | 0.331 |
| 89 | Trim39 | -4.86 | -0.32 | 0.0083 | 0.331 |
| 90 | Fzd6 | -4.37 | -0.32 | 0.0119 | 0.354 |
| 91 | Actrt1 | -5.45 | -0.32 | 0.0055 | 0.315 |
| 92 | Btaf1 | -4.97 | -0.32 | 0.0076 | 0.322 |
| 93 | Nin | -5.15 | -0.32 | 0.0067 | 0.322 |
| 94 | Mrpl36 | -8.37 | -0.32 | 0.0011 | 0.232 |
| 95 | Slc27a6 | -4.82 | -0.32 | 0.0085 | 0.331 |
| 96 | Chac1 | -4.22 | -0.32 | 0.0135 | 0.358 |
| 97 | E030019B06Rik | -4.18 | -0.32 | 0.0140 | 0.362 |
| 98 | A930018M24Rik | -4.96 | -0.32 | 0.0077 | 0.322 |
| 99 | 5730442P18Rik | -6.51 | -0.32 | 0.0029 | 0.287 |
| 100 | B930008K04Rik | -4.13 | -0.31 | 0.0145 | 0.362 |
| 101 | Stag3 | -4.46 | -0.31 | 0.0112 | 0.353 |
| 102 | AW822216 | -8.93 | -0.31 | 0.0009 | 0.224 |
| 103 | Tpx2 | -9.51 | -0.31 | 0.0007 | 0.217 |
| 104 | Arpc1b | -4.40 | -0.31 | 0.0116 | 0.354 |
| 105 | 4933427D14Rik | -5.76 | -0.31 | 0.0045 | 0.299 |
| 106 | Eps15l1 | -5.33 | -0.31 | 0.0060 | 0.319 |
| 107 | Tspan14 | -5.96 | -0.30 | 0.0040 | 0.299 |
| 108 | Ddx11 | -4.23 | -0.30 | 0.0134 | 0.358 |
| 109 | Ramp2 | -5.97 | -0.30 | 0.0040 | 0.299 |
| 110 | Trpm8 | -4.77 | -0.30 | 0.0088 | 0.334 |
| 111 | Pld1 | -4.75 | -0.30 | 0.0090 | 0.336 |
| 112 | Tmem56 | -6.13 | -0.30 | 0.0036 | 0.298 |
