# Supplementary material for: Neonatal Maternal Deprivation Response and Developmental Changes in Gene Expression Revealed by Hypothalamic Gene Expression Profiling in Mice
Source: PLoS One. 2010 Feb 24;5(2):e9402. doi: 10.1371/journal.pone.0009402 (PMC2827556; doi:10.1371/journal.pone.0009402)
Supplement: Table S2 — Comparison 4. P5 Snord116del starved vs non-starved (2-AD, 6-CD, 10-ED, 12-FD) vs. (4-BD, 8-DD). (0.18 MB DOC) [file pone.0009402.s003.doc]

Table S2. Comparison 4. P5 Snord116del starved vs non-starved (2-AD, 6-CD, 10-ED,

12-FD) vs. (4-BD, 8-DD). t-value is the ratio of the estimated change divided by the standard error.

BH-FDR: Bonferroni Hochberg false discovery rate. High-lighted are genes validated by

quantitative RT-PCR as shown in table 1.

| Comp.4 | Gene Symbol | t-value | fold_change | p-value | BH-FDR |
| --- | --- | --- | --- | --- | --- |
| 1 | Nanos2 | 6.40 | 0.82 | 0.0031 | 0.339 |
| 2 | 1700093K21Rik | 7.56 | 0.75 | 0.0016 | 0.319 |
| 3 | 9830001H06Rik | 7.93 | 0.65 | 0.0014 | 0.319 |
| 4 | Gabrd | 4.11 | 0.59 | 0.0148 | 0.389 |
| 5 | Klhl15 | 4.09 | 0.58 | 0.0149 | 0.389 |
| 6 | Btf3l4 | 6.77 | 0.57 | 0.0025 | 0.339 |
| 7 | Mrg1 | 4.25 | 0.56 | 0.0132 | 0.388 |
| 8 | Ccl17 | 6.08 | 0.53 | 0.0037 | 0.339 |
| 9 | Pdk4 | 4.59 | 0.52 | 0.0101 | 0.379 |
| 10 | Nploc4 | 5.63 | 0.51 | 0.0049 | 0.339 |
| 11 | Dgcr8 | 7.01 | 0.51 | 0.0022 | 0.334 |
| 12 | Plvap | 7.42 | 0.49 | 0.0018 | 0.319 |
| 13 | Ly6g5b | 4.46 | 0.48 | 0.0112 | 0.379 |
| 14 | Eif2ak3 | 4.74 | 0.48 | 0.0090 | 0.376 |
| 15 | Prrc1 | 9.72 | 0.48 | 0.0006 | 0.289 |
| 16 | Thsd1 | 6.31 | 0.48 | 0.0032 | 0.339 |
| 17 | Olfr1155 | 4.51 | 0.47 | 0.0107 | 0.379 |
| 18 | Art5 | 9.18 | 0.47 | 0.0008 | 0.289 |
| 19 | Cdk3 | 4.84 | 0.46 | 0.0084 | 0.370 |
| 20 | Suv420h1 | 7.50 | 0.45 | 0.0017 | 0.319 |
| 21 | Lrrn2 | 5.20 | 0.45 | 0.0065 | 0.351 |
| 22 | Nol11 | 4.46 | 0.45 | 0.0111 | 0.379 |
| 23 | Ddx19a | 4.63 | 0.44 | 0.0098 | 0.379 |
| 24 | Tmem14a | 10.96 | 0.44 | 0.0004 | 0.262 |
| 25 | Mospd3 | 6.37 | 0.44 | 0.0031 | 0.339 |
| 26 | Sp5 | 5.66 | 0.44 | 0.0048 | 0.339 |
| 27 | Mmp15 | 9.27 | 0.43 | 0.0008 | 0.289 |
| 28 | Ptprn2 | 5.83 | 0.43 | 0.0043 | 0.339 |
| 29 | Arnt2 | 4.16 | 0.43 | 0.0142 | 0.388 |
| 30 | Epb4.2 | 7.34 | 0.42 | 0.0018 | 0.319 |
| 31 | Rfc3 | 5.95 | 0.42 | 0.0040 | 0.339 |
| 32 | Rbm16 | 6.88 | 0.42 | 0.0023 | 0.338 |
| 33 | Samd4b | 4.17 | 0.42 | 0.0140 | 0.388 |
| 34 | Zfp335 | 4.51 | 0.42 | 0.0107 | 0.379 |
| 35 | Grpel1 | 7.75 | 0.41 | 0.0015 | 0.319 |
| 36 | Odz4 | 8.86 | 0.41 | 0.0009 | 0.304 |
| 37 | Arih2 | 4.06 | 0.41 | 0.0154 | 0.389 |
| 38 | Olfr1232 | 12.21 | 0.40 | 0.0003 | 0.214 |
| 39 | Znrf2 | 4.22 | 0.40 | 0.0135 | 0.388 |
| 40 | Uvrag | 4.35 | 0.40 | 0.0121 | 0.381 |
| 41 | Aloxe3 | 7.49 | 0.39 | 0.0017 | 0.319 |
| 42 | Dicer1 | 4.65 | 0.39 | 0.0097 | 0.379 |
| 43 | Ahctf1 | 4.03 | 0.39 | 0.0157 | 0.389 |
| 44 | Nlrp4e | 7.83 | 0.38 | 0.0014 | 0.319 |
| 45 | Olfr1143 | 4.83 | 0.38 | 0.0084 | 0.370 |
| 46 | Kif26b | 8.20 | 0.38 | 0.0012 | 0.319 |
| 47 | B930041F14Rik | 9.11 | 0.38 | 0.0008 | 0.289 |
| 48 | Olfr676 | 4.03 | 0.38 | 0.0157 | 0.389 |
| 49 | Rbmx | 6.05 | 0.38 | 0.0038 | 0.339 |
| 50 | Fhod3 | 5.07 | 0.37 | 0.0072 | 0.363 |
| 51 | Lins2 | 4.57 | 0.37 | 0.0103 | 0.379 |
| 52 | Tlx1 | 5.02 | 0.37 | 0.0074 | 0.363 |
| 53 | Pde4c | 5.46 | 0.37 | 0.0055 | 0.339 |
| 54 | Jup | 4.92 | 0.36 | 0.0080 | 0.366 |
| 55 | Olfr124 | 6.58 | 0.36 | 0.0028 | 0.339 |
| 56 | Pde2a | 4.17 | 0.36 | 0.0140 | 0.388 |
| 57 | Kiss1 | 4.87 | 0.36 | 0.0082 | 0.370 |
| 58 | Lsm11 | 5.33 | 0.35 | 0.0060 | 0.339 |
| 59 | Armc5 | 4.14 | 0.35 | 0.0144 | 0.388 |
| 60 | Dlx2 | 4.95 | 0.35 | 0.0078 | 0.365 |
| 61 | Zkscan17 | 4.39 | 0.35 | 0.0117 | 0.379 |
| 62 | Kifc3 | 6.26 | 0.35 | 0.0033 | 0.339 |
| 63 | D10Wsu102e | 5.71 | 0.35 | 0.0047 | 0.339 |
| 64 | Olfm2 | 10.65 | 0.35 | 0.0004 | 0.262 |
| 65 | Yrdc | 8.46 | 0.35 | 0.0011 | 0.313 |
| 66 | Jund1 | 4.94 | 0.35 | 0.0078 | 0.365 |
| 67 | Kif21b | 5.30 | 0.34 | 0.0061 | 0.339 |
| 68 | Mtap6 | 5.50 | 0.34 | 0.0053 | 0.339 |
| 69 | Fkbp7 | 7.23 | 0.34 | 0.0019 | 0.321 |
| 70 | 5730590G19Rik | 6.51 | 0.34 | 0.0029 | 0.339 |
| 71 | Tns3 | 9.66 | 0.34 | 0.0006 | 0.289 |
| 72 | Timp2 | 5.31 | 0.34 | 0.0060 | 0.339 |
| 73 | Pigr | 4.10 | 0.34 | 0.0148 | 0.389 |
| 74 | Sez6l2 | 4.53 | 0.34 | 0.0106 | 0.379 |
| 75 | Gpr6 | 4.19 | 0.34 | 0.0138 | 0.388 |
| 76 | Cant1 | 4.10 | 0.34 | 0.0149 | 0.389 |
| 77 | 1110051M20Rik | 4.52 | 0.34 | 0.0107 | 0.379 |
| 78 | Olfr461 | 6.68 | 0.34 | 0.0026 | 0.339 |
| 79 | Map3k11 | 5.34 | 0.34 | 0.0059 | 0.339 |
| 80 | Ltb4r1 | 4.77 | 0.33 | 0.0089 | 0.376 |
| 81 | Usp30 | 4.20 | 0.33 | 0.0136 | 0.388 |
| 82 | Onecut2 | 5.25 | 0.33 | 0.0063 | 0.345 |
| 83 | Gmeb2 | 7.75 | 0.33 | 0.0015 | 0.319 |
| 84 | 4833446K15Rik | 4.28 | 0.33 | 0.0129 | 0.388 |
| 85 | Lrpprc | 5.02 | 0.32 | 0.0074 | 0.363 |
| 86 | 2810485I05Rik | 6.08 | 0.32 | 0.0037 | 0.339 |
| 87 | Mtac2d1 | 5.81 | 0.32 | 0.0044 | 0.339 |
| 88 | Rpe | 4.37 | 0.32 | 0.0119 | 0.380 |
| 89 | Armcx2 | 4.97 | 0.32 | 0.0076 | 0.365 |
| 90 | Hcn3 | 4.24 | 0.32 | 0.0133 | 0.388 |
| 91 | Tnnc2 | 11.19 | 0.32 | 0.0004 | 0.255 |
| 92 | Nucb2 | 5.93 | 0.32 | 0.0040 | 0.339 |
| 93 | Prkab1 | 6.27 | 0.32 | 0.0033 | 0.339 |
| 94 | Fgl2 | 4.69 | 0.32 | 0.0094 | 0.379 |
| 95 | Wdr61 | 10.34 | 0.32 | 0.0005 | 0.262 |
| 96 | 1700041E20Rik | 6.07 | 0.32 | 0.0037 | 0.339 |
| 97 | Zdhhc3 | 4.38 | 0.31 | 0.0119 | 0.380 |
| 98 | Mon1b | 5.32 | 0.31 | 0.0060 | 0.339 |
| 99 | Dnttip2 | 9.19 | 0.31 | 0.0008 | 0.289 |
| 100 | Pvrl1 | 4.50 | 0.31 | 0.0108 | 0.379 |
| 101 | 6330417G02Rik | 4.04 | 0.31 | 0.0156 | 0.389 |
| 102 | Slc15a3 | 4.40 | 0.31 | 0.0117 | 0.379 |
| 103 | Kcnb1 | 5.47 | 0.31 | 0.0054 | 0.339 |
| 104 | Amigo3 | 5.80 | 0.31 | 0.0044 | 0.339 |
| 105 | Dbf4 | 4.89 | 0.31 | 0.0081 | 0.370 |
| 106 | Tas1r2 | 4.60 | 0.31 | 0.0100 | 0.379 |
| 107 | Mier3 | 5.78 | 0.30 | 0.0044 | 0.339 |
| 108 | 2810046M22Rik | 4.14 | 0.30 | 0.0143 | 0.388 |
| 109 | 1600029D21Rik | 5.30 | 0.30 | 0.0061 | 0.339 |

| Comp4 | Symbol | t-value | fold_change | p-value | BH-FDR |
| --- | --- | --- | --- | --- | --- |
| 1 | Ptpn9 | -6.99 | -0.94 | 0.0022 | 0.334 |
| 2 | Pgpep1 | -5.74 | -0.80 | 0.0046 | 0.339 |
| 3 | Sparc | -4.08 | -0.73 | 0.0150 | 0.389 |
| 4 | Edd1 | -8.34 | -0.70 | 0.0011 | 0.315 |
| 5 | Plp1 | -5.87 | -0.67 | 0.0042 | 0.339 |
| 6 | 2900041A09Rik | -4.98 | -0.64 | 0.0076 | 0.364 |
| 7 | S100a1 | -12.10 | -0.63 | 0.0003 | 0.214 |
| 8 | Ing2 | -4.92 | -0.62 | 0.0079 | 0.366 |
| 9 | Tmem100 | -12.18 | -0.59 | 0.0003 | 0.214 |
| 10 | Rel | -5.17 | -0.58 | 0.0066 | 0.352 |
| 11 | Ncaph | -5.01 | -0.58 | 0.0074 | 0.363 |
| 12 | Ciao1 | -13.68 | -0.58 | 0.0002 | 0.201 |
| 13 | Zfp420 | -5.19 | -0.55 | 0.0065 | 0.351 |
| 14 | Plekha8 | -8.70 | -0.55 | 0.0010 | 0.308 |
| 15 | Trhr | -4.47 | -0.55 | 0.0111 | 0.379 |
| 16 | C87436 | -7.62 | -0.55 | 0.0016 | 0.319 |
| 17 | Tmem142c | -5.66 | -0.54 | 0.0048 | 0.339 |
| 18 | Bcas1 | -5.00 | -0.53 | 0.0075 | 0.363 |
| 19 | Ndph | -17.47 | -0.53 | 0.0001 | 0.188 |
| 20 | Mterfd3 | -4.15 | -0.52 | 0.0143 | 0.388 |
| 21 | 1700022C21Rik | -7.77 | -0.52 | 0.0015 | 0.319 |
| 22 | AU020772 | -4.11 | -0.50 | 0.0148 | 0.389 |
| 23 | Gdf9 | -4.24 | -0.50 | 0.0133 | 0.388 |
| 24 | Mett10d | -5.67 | -0.50 | 0.0048 | 0.339 |
| 25 | Tomm34 | -4.24 | -0.49 | 0.0132 | 0.388 |
| 26 | D430028G21Rik | -22.91 | -0.47 | 0.0000 | 0.171 |
| 27 | C030039L03Rik | -4.44 | -0.47 | 0.0113 | 0.379 |
| 28 | Ttc8 | -4.81 | -0.46 | 0.0086 | 0.372 |
| 29 | Gsto2 | -4.71 | -0.46 | 0.0093 | 0.379 |
| 30 | Brms1l | -5.63 | -0.45 | 0.0049 | 0.339 |
| 31 | Pkm2 | -10.38 | -0.45 | 0.0005 | 0.262 |
| 32 | D10Jhu81e | -14.28 | -0.44 | 0.0001 | 0.201 |
| 33 | Senp8 | -5.20 | -0.44 | 0.0065 | 0.351 |
| 34 | 4632417K18Rik | -4.15 | -0.44 | 0.0143 | 0.388 |
| 35 | Mkks | -7.25 | -0.44 | 0.0019 | 0.321 |
| 36 | Tgm2 | -7.22 | -0.43 | 0.0020 | 0.321 |
| 37 | St6gal1 | -5.59 | -0.43 | 0.0050 | 0.339 |
| 38 | Lrrtm2 | -4.05 | -0.42 | 0.0155 | 0.389 |
| 39 | 9130227C08Rik | -5.15 | -0.42 | 0.0067 | 0.356 |
| 40 | Osr2 | -7.43 | -0.42 | 0.0017 | 0.319 |
| 41 | Mitd1 | -10.23 | -0.42 | 0.0005 | 0.264 |
| 42 | Hes5 | -4.73 | -0.42 | 0.0091 | 0.376 |
| 43 | Afg3l2 | -4.95 | -0.41 | 0.0077 | 0.365 |
| 44 | Id4 | -4.50 | -0.41 | 0.0108 | 0.379 |
| 45 | Kif3a | -4.16 | -0.41 | 0.0141 | 0.388 |
| 46 | Rpusd4 | -17.94 | -0.41 | 0.0001 | 0.188 |
| 47 | Elavl1 | -4.88 | -0.41 | 0.0082 | 0.370 |
| 48 | Mill2 | -4.14 | -0.40 | 0.0144 | 0.388 |
| 49 | 0610007P14Rik | -7.43 | -0.40 | 0.0018 | 0.319 |
| 50 | Ppnr | -5.15 | -0.39 | 0.0068 | 0.356 |
| 51 | Mettl8 | -5.99 | -0.39 | 0.0039 | 0.339 |
| 52 | RP23-143A14.5 | -4.09 | -0.39 | 0.0150 | 0.389 |
| 53 | Cst10 | -8.60 | -0.39 | 0.0010 | 0.313 |
| 54 | Samd9l | -5.64 | -0.38 | 0.0049 | 0.339 |
| 55 | Ncbp2 | -5.04 | -0.38 | 0.0073 | 0.363 |
| 56 | Chchd7 | -4.61 | -0.38 | 0.0100 | 0.379 |
| 57 | 2310007A19Rik | -6.03 | -0.37 | 0.0038 | 0.339 |
| 58 | Tph1 | -4.54 | -0.37 | 0.0105 | 0.379 |
| 59 | Mrpl19 | -4.41 | -0.37 | 0.0116 | 0.379 |
| 60 | Diap3 | -5.12 | -0.37 | 0.0069 | 0.360 |
| 61 | S100a13 | -4.05 | -0.37 | 0.0155 | 0.389 |
| 62 | Rp1h | -5.65 | -0.37 | 0.0048 | 0.339 |
| 63 | Abcg3 | -4.04 | -0.37 | 0.0156 | 0.389 |
| 64 | Cdc91l1 | -5.39 | -0.37 | 0.0057 | 0.339 |
| 65 | Sh3bgrl2 | -15.15 | -0.36 | 0.0001 | 0.196 |
| 66 | Npal2 | -5.91 | -0.36 | 0.0041 | 0.339 |
| 67 | Ebp | -9.84 | -0.36 | 0.0006 | 0.289 |
| 68 | Slc22a4 | -5.41 | -0.36 | 0.0056 | 0.339 |
| 69 | Tmem177 | -4.26 | -0.36 | 0.0131 | 0.388 |
| 70 | Mfsd9 | -4.67 | -0.36 | 0.0095 | 0.379 |
| 71 | V1rc14 | -4.17 | -0.36 | 0.0140 | 0.388 |
| 72 | 9630041N07Rik | -5.52 | -0.36 | 0.0052 | 0.339 |
| 73 | Mphosph1 | -5.09 | -0.35 | 0.0070 | 0.363 |
| 74 | Rgs7bp | -4.71 | -0.35 | 0.0092 | 0.379 |
| 75 | Ankrd15 | -7.94 | -0.35 | 0.0014 | 0.319 |
| 76 | Papd5 | -4.11 | -0.35 | 0.0147 | 0.389 |
| 77 | Gtf2h3 | -4.82 | -0.35 | 0.0085 | 0.371 |
| 78 | Olfr1471 | -6.53 | -0.35 | 0.0028 | 0.339 |
| 79 | Angptl4 | -4.73 | -0.35 | 0.0091 | 0.376 |
| 80 | Rbpms | -4.44 | -0.35 | 0.0113 | 0.379 |
| 81 | Ms4a6c | -6.98 | -0.35 | 0.0022 | 0.334 |
| 82 | Card11 | -5.82 | -0.34 | 0.0043 | 0.339 |
| 83 | Phkg1 | -4.11 | -0.34 | 0.0147 | 0.389 |
| 84 | Stxbp4 | -6.82 | -0.34 | 0.0024 | 0.339 |
| 85 | Negr1 | -4.77 | -0.34 | 0.0088 | 0.376 |
| 86 | Oma1 | -4.42 | -0.34 | 0.0115 | 0.379 |
| 87 | Eftud1 | -6.40 | -0.34 | 0.0031 | 0.339 |
| 88 | 1190002H23Rik | -4.95 | -0.34 | 0.0077 | 0.365 |
| 89 | D530033C11Rik | -6.59 | -0.34 | 0.0028 | 0.339 |
| 90 | Ak3l1 | -4.22 | -0.34 | 0.0135 | 0.388 |
| 91 | Poli | -7.06 | -0.33 | 0.0021 | 0.334 |
| 92 | S100a10 | -5.55 | -0.33 | 0.0052 | 0.339 |
| 93 | Mrgprb5 | -5.34 | -0.33 | 0.0059 | 0.339 |
| 94 | Cml1 | -7.34 | -0.33 | 0.0018 | 0.319 |
| 95 | Slc8a3 | -5.99 | -0.33 | 0.0039 | 0.339 |
| 96 | Zgpat | -5.68 | -0.32 | 0.0048 | 0.339 |
| 97 | Sec11a | -4.74 | -0.32 | 0.0090 | 0.376 |
| 98 | 9530077C05Rik | -5.58 | -0.32 | 0.0051 | 0.339 |
| 99 | 2700049A03Rik | -4.51 | -0.32 | 0.0108 | 0.379 |
| 100 | Rab27a | -13.22 | -0.32 | 0.0002 | 0.201 |
| 101 | 2310016C16Rik | -7.92 | -0.32 | 0.0014 | 0.319 |
| 102 | Ptger2 | -4.17 | -0.32 | 0.0141 | 0.388 |
| 103 | Mesdc1 | -8.69 | -0.32 | 0.0010 | 0.308 |
| 104 | Gpr68 | -6.58 | -0.32 | 0.0028 | 0.339 |
| 105 | Gprc5b | -5.11 | -0.32 | 0.0069 | 0.361 |
| 106 | Mfsd8 | -4.50 | -0.32 | 0.0108 | 0.379 |
| 107 | Zfyve21 | -6.47 | -0.31 | 0.0029 | 0.339 |
| 108 | Chac1 | -7.95 | -0.31 | 0.0014 | 0.319 |
| 109 | Prlpk | -6.11 | -0.31 | 0.0036 | 0.339 |
| 110 | 1700034H14Rik | -5.23 | -0.31 | 0.0064 | 0.350 |
| 111 | Zbtb3 | -9.11 | -0.31 | 0.0008 | 0.289 |
| 112 | Rxfp4 | -4.44 | -0.31 | 0.0113 | 0.379 |
| 113 | Zdhhc8 | -6.03 | -0.31 | 0.0038 | 0.339 |
| 114 | E130012A19Rik | -5.32 | -0.31 | 0.0060 | 0.339 |
| 115 | Leprotl1 | -4.26 | -0.31 | 0.0131 | 0.388 |
| 116 | Mrpl24 | -6.50 | -0.30 | 0.0029 | 0.339 |
| 117 | 3110003A17Rik | -4.32 | -0.30 | 0.0124 | 0.385 |
| 118 | Josd2 | -5.04 | -0.30 | 0.0073 | 0.363 |
| 119 | Edg8 | -5.59 | -0.30 | 0.0050 | 0.339 |
| 120 | Olfr1260 | -4.21 | -0.30 | 0.0136 | 0.388 |
| 121 | Pomc1 | -5.36 | -0.30 | 0.0058 | 0.339 |
