# Supplementary material for: Neonatal Maternal Deprivation Response and Developmental Changes in Gene Expression Revealed by Hypothalamic Gene Expression Profiling in Mice
Source: PLoS One. 2010 Feb 24;5(2):e9402. doi: 10.1371/journal.pone.0009402 (PMC2827556; doi:10.1371/journal.pone.0009402)
Supplement: Table S3 — Comparison 6. P5 wild type vs. P13 wild type (1-AW, 5-CW, 9-EW, 11-FW) vs.(13-GW. 15-HW) (0.61 MB DOC) [file pone.0009402.s004.doc]

Table S3. Comparison 6. P5 wild type vs. P13 wild type (1-AW, 5-CW, 9-EW, 11-FW) vs.(13-GW. 15-HW) t-value is the ratio of the estimated change divided by the standard error.

BH-FDR: Bonferroni Hochberg false discovery rate.

| Comp 6 | Gene Symbol | | t-value | | fold_change | | p-value | | BH-FDR | |
| --- | --- | --- | --- | --- | --- | --- | --- | --- | --- | --- |
| 1 | Mobp | | 27.25 | | 5.74 | | 1.08E-05 | | 0.007 | |
| 2 | Mag | | 22.12 | | 3.75 | | 2.47E-05 | | 0.009 | |
| 3 | Mog | | 18.89 | | 3.71 | | 4.62E-05 | | 0.011 | |
| 4 | Mbp | | 17.12 | | 3.15 | | 6.82E-05 | | 0.012 | |
| 5 | Plp1 | | 16.00 | | 3.13 | | 8.92E-05 | | 0.013 | |
| 6 | Ugt8a | | 12.93 | | 3.01 | | 0.000207 | | 0.017 | |
| 7 | Cldn11 | | 13.70 | | 2.92 | | 0.000165 | | 0.016 | |
| 8 | Fa2h | | 17.33 | | 2.73 | | 6.51E-05 | | 0.012 | |
| 9 | BC055107 | | 12.46 | | 2.71 | | 0.000238 | | 0.017 | |
| 10 | 6330530A05Rik | | 65.29 | | 2.41 | | 3.3E-07 | | 0.002 | |
| 11 | Tmem10 | | 12.93 | | 2.37 | | 0.000207 | | 0.017 | |
| 12 | Trf | | 16.21 | | 2.16 | | 8.46E-05 | | 0.013 | |
| 13 | Ntsr2 | | 20.77 | | 2.07 | | 3.18E-05 | | 0.009 | |
| 14 | Enpp6 | | 13.99 | | 1.98 | | 0.000151 | | 0.015 | |
| 15 | Adamts4 | | 19.52 | | 1.93 | | 4.06E-05 | | 0.011 | |
| 16 | 9530066K23Rik | | 9.63 | | 1.83 | | 0.00065 | | 0.025 | |
| 17 | Gja12 | | 18.20 | | 1.83 | | 5.36E-05 | | 0.012 | |
| 18 | 2900041A09Rik | | 21.02 | | 1.82 | | 3.03E-05 | | 0.009 | |
| 19 | Lgi3 | | 15.65 | | 1.81 | | 9.73E-05 | | 0.013 | |
| 20 | Oxt | | 11.11 | | 1.78 | | 0.000373 | | 0.020 | |
| 21 | Rhbdl3 | | 17.72 | | 1.75 | | 5.96E-05 | | 0.012 | |
| 22 | Hrh3 | | 18.22 | | 1.73 | | 5.34E-05 | | 0.012 | |
| 23 | Bcas1 | | 10.19 | | 1.71 | | 0.000522 | | 0.023 | |
| 24 | Tmem166 | | 7.71 | | 1.70 | | 0.00152 | | 0.035 | |
| 25 | Gjb6 | | 8.81 | | 1.69 | | 0.000915 | | 0.028 | |
| 26 | Selplg | | 15.15 | | 1.64 | | 0.000111 | | 0.014 | |
| 27 | Klf9 | | 8.76 | | 1.62 | | 0.000938 | | 0.028 | |
| 28 | Gprc5b | | 12.30 | | 1.58 | | 0.000251 | | 0.017 | |
| 29 | Csrp1 | | 14.58 | | 1.54 | | 0.000129 | | 0.014 | |
| 30 | Hapln4 | | 12.03 | | 1.54 | | 0.000274 | | 0.018 | |
| 31 | Sparcl1 | | 9.23 | | 1.51 | | 0.000766 | | 0.026 | |
| 32 | Grin2c | | 17.38 | | 1.50 | | 6.43E-05 | | 0.012 | |
| 33 | Mbnl2 | | 11.31 | | 1.48 | | 0.000349 | | 0.020 | |
| 34 | Plxnb3 | | 8.05 | | 1.46 | | 0.001292 | | 0.033 | |
| 35 | Gpd1 | | 21.86 | | 1.46 | | 2.59E-05 | | 0.009 | |
| 36 | Sdc4 | | 7.51 | | 1.45 | | 0.001685 | | 0.037 | |
| 37 | Nefh | | 7.68 | | 1.43 | | 0.001545 | | 0.036 | |
| 38 | Tmem119 | | 10.12 | | 1.40 | | 0.000537 | | 0.023 | |
| 39 | Cartpt | | 21.07 | | 1.39 | | 3E-05 | | 0.009 | |
| 40 | Itih3 | | 6.73 | | 1.39 | | 0.002542 | | 0.044 | |
| 41 | Purb | | 8.95 | | 1.39 | | 0.000864 | | 0.027 | |
| 42 | Pvalb | | 9.34 | | 1.38 | | 0.000733 | | 0.026 | |
| 43 | Cnp1 | | 11.12 | | 1.37 | | 0.000372 | | 0.020 | |
| 44 | Gpr37l1 | | 23.57 | | 1.37 | | 1.92E-05 | | 0.008 | |
| 45 | Mgp | | 17.39 | | 1.36 | | 6.42E-05 | | 0.012 | |
| 46 | Ankrd15 | | 9.39 | | 1.36 | | 0.000718 | | 0.026 | |
| 47 | Cox6a2 | | 13.38 | | 1.35 | | 0.000181 | | 0.016 | |
| 48 | Ogfod1 | | 16.75 | | 1.34 | | 7.44E-05 | | 0.013 | |
| 49 | Dbp | | 10.83 | | 1.32 | | 0.000413 | | 0.021 | |
| 50 | Susd2 | | 24.70 | | 1.30 | | 1.6E-05 | | 0.008 | |
| 51 | Mal | | 7.25 | | 1.29 | | 0.001917 | | 0.039 | |
| 52 | Htra1 | | 11.09 | | 1.23 | | 0.000377 | | 0.021 | |
| 53 | Emb | | 9.66 | | 1.23 | | 0.000642 | | 0.025 | |
| 54 | Lims2 | | 14.22 | | 1.23 | | 0.000142 | | 0.015 | |
| 55 | Cyp2d22 | | 8.99 | | 1.23 | | 0.000847 | | 0.027 | |
| 56 | Sirt2 | | 9.67 | | 1.20 | | 0.00064 | | 0.025 | |
| 57 | Traf3 | | 24.77 | | 1.19 | | 1.58E-05 | | 0.008 | |
| 58 | Aldoc | | 12.86 | | 1.18 | | 0.000211 | | 0.017 | |
| 59 | Kcnj16 | | 9.32 | | 1.18 | | 0.000738 | | 0.026 | |
| 60 | Pllp | | 10.46 | | 1.17 | | 0.000472 | | 0.022 | |
| 61 | Zfp612 | | 8.09 | | 1.17 | | 0.00127 | | 0.032 | |
| 62 | Gpr83 | | 9.77 | | 1.16 | | 0.000614 | | 0.025 | |
| 63 | Efhd1 | | 7.52 | | 1.15 | | 0.001673 | | 0.037 | |
| 64 | Sult1a1 | | 8.35 | | 1.14 | | 0.001124 | | 0.031 | |
| 65 | 1700020C11Rik | | 16.70 | | 1.14 | | 7.54E-05 | | 0.013 | |
| 66 | Kcnip4 | | 12.88 | | 1.14 | | 0.00021 | | 0.017 | |
| 67 | Aldh1a1 | | 9.22 | | 1.14 | | 0.000767 | | 0.026 | |
| 68 | Kcna2 | | 15.91 | | 1.12 | | 9.12E-05 | | 0.013 | |
| 69 | Otub1 | | 12.13 | | 1.11 | | 0.000265 | | 0.018 | |
| 70 | Plekhh1 | | 17.24 | | 1.10 | | 6.64E-05 | | 0.012 | |
| 71 | Tmem59l | | 12.04 | | 1.09 | | 0.000273 | | 0.018 | |
| 72 | Sorl1 | | 10.20 | | 1.08 | | 0.000521 | | 0.023 | |
| 73 | 6330527O06Rik | | 7.25 | | 1.08 | | 0.001923 | | 0.039 | |
| 74 | Extl1 | | 17.75 | | 1.08 | | 5.92E-05 | | 0.012 | |
| 75 | Gltp | | 8.38 | | 1.07 | | 0.001108 | | 0.030 | |
| 76 | Abhd3 | | 76.78 | | 1.07 | | 1.72E-07 | | 0.002 | |
| 77 | Rasd1 | | 7.43 | | 1.05 | | 0.001756 | | 0.037 | |
| 78 | Pls3 | | 6.32 | | 1.04 | | 0.003205 | | 0.050 | |
| 79 | Cyp46a1 | | 8.32 | | 1.03 | | 0.001138 | | 0.031 | |
| 80 | Agt | | 7.36 | | 1.03 | | 0.001814 | | 0.038 | |
| 81 | Insl3 | | 7.85 | | 1.03 | | 0.001422 | | 0.034 | |
| 82 | Hdac11 | | 10.42 | | 1.03 | | 0.000479 | | 0.022 | |
| 83 | Edg8 | | 8.32 | | 1.02 | | 0.001141 | | 0.031 | |
| 84 | Nckipsd | | 12.31 | | 1.02 | | 0.00025 | | 0.017 | |
| 85 | Scn1b | | 11.59 | | 1.02 | | 0.000316 | | 0.019 | |
| 86 | Slc44a1 | | 13.57 | | 1.02 | | 0.000171 | | 0.016 | |
| 87 | Pip5k1b | | 20.73 | | 1.01 | | 3.2E-05 | | 0.009 | |
| 88 | Lynx1 | | 25.01 | | 1.01 | | 1.52E-05 | | 0.008 | |
| 89 | Trh | | 9.65 | | 0.99 | | 0.000645 | | 0.025 | |
| 90 | Cbx7 | | 15.30 | | 0.98 | | 0.000106 | | 0.014 | |
| 91 | D16H22S680E | | 7.69 | | 0.98 | | 0.00154 | | 0.036 | |
| 92 | Nat8l | | 14.23 | | 0.97 | | 0.000142 | | 0.015 | |
| 93 | Arpc1b | | 13.72 | | 0.97 | | 0.000163 | | 0.016 | |
| 94 | Gstt3 | | 7.43 | | 0.96 | | 0.001756 | | 0.037 | |
| 95 | Car4 | | 8.84 | | 0.96 | | 0.000905 | | 0.028 | |
| 96 | Pnck | | 14.58 | | 0.96 | | 0.000129 | | 0.014 | |
| 97 | LOC671769 | | 12.67 | | 0.96 | | 0.000223 | | 0.017 | |
| 98 | Neu4 | | 11.96 | | 0.96 | | 0.00028 | | 0.018 | |
| 99 | Fbxo2 | | 9.90 | | 0.94 | | 0.000584 | | 0.024 | |
| 100 | Itpr1 | | 6.77 | | 0.93 | | 0.002485 | | 0.044 | |
| 101 | Pdk2 | | 8.10 | | 0.93 | | 0.00126 | | 0.032 | |
| 102 | Slc45a3 | | 11.81 | | 0.92 | | 0.000294 | | 0.018 | |
| 103 | Rogdi | | 11.71 | | 0.92 | | 0.000304 | | 0.019 | |
| 104 | Cryab | | 20.11 | | 0.92 | | 3.61E-05 | | 0.010 | |
| 105 | Atp1b2 | | 6.71 | | 0.92 | | 0.002575 | | 0.045 | |
| 106 | Ar | | 13.94 | | 0.91 | | 0.000154 | | 0.015 | |
| 107 | Slc7a10 | | 8.10 | | 0.91 | | 0.001262 | | 0.032 | |
| 108 | Cmtm5 | | 6.79 | | 0.91 | | 0.002462 | | 0.044 | |
| 109 | A930037G23Rik | | 7.31 | | 0.91 | | 0.001865 | | 0.039 | |
| 110 | Gpld1 | | 18.83 | | 0.91 | | 4.68E-05 | | 0.011 | |
| 111 | Gng13 | | 11.51 | | 0.90 | | 0.000325 | | 0.019 | |
| 112 | Elovl7 | | 10.79 | | 0.89 | | 0.000419 | | 0.021 | |
| 113 | Spsb1 | | 6.75 | | 0.89 | | 0.002505 | | 0.044 | |
| 114 | Kcnab2 | | 9.71 | | 0.88 | | 0.00063 | | 0.025 | |
| 115 | Eno2 | | 7.04 | | 0.87 | | 0.002141 | | 0.041 | |
| 116 | Josd2 | | 7.26 | | 0.87 | | 0.001914 | | 0.039 | |
| 117 | Abca2 | | 7.64 | | 0.87 | | 0.001574 | | 0.036 | |
| 118 | Dusp14 | | 9.54 | | 0.87 | | 0.000674 | | 0.026 | |
| 119 | Emcn | | 10.09 | | 0.87 | | 0.000542 | | 0.023 | |
| 120 | Mertk | | 8.46 | | 0.85 | | 0.001071 | | 0.030 | |
| 121 | Sh3bgrl2 | | 7.03 | | 0.85 | | 0.002157 | | 0.041 | |
| 122 | Agrp | | 7.17 | | 0.85 | | 0.002005 | | 0.039 | |
| 123 | Rab3gap1 | | 7.12 | | 0.85 | | 0.002059 | | 0.040 | |
| 124 | Sox10 | | 12.36 | | 0.84 | | 0.000246 | | 0.017 | |
| 125 | Eltd1 | | 17.49 | | 0.84 | | 6.27E-05 | | 0.012 | |
| 126 | Fgf1 | | 35.58 | | 0.84 | | 3.72E-06 | | 0.005 | |
| 127 | Cyp2j9 | | 14.05 | | 0.84 | | 0.000149 | | 0.015 | |
| 128 | Atp5a1 | | 10.58 | | 0.83 | | 0.000451 | | 0.022 | |
| 129 | C920006C10Rik | | 11.94 | | 0.83 | | 0.000281 | | 0.018 | |
| 130 | Bfsp2 | | 8.11 | | 0.83 | | 0.001259 | | 0.032 | |
| 131 | Gamt | | 10.34 | | 0.83 | | 0.000494 | | 0.022 | |
| 132 | Tnfrsf12a | | 13.31 | | 0.82 | | 0.000184 | | 0.016 | |
| 133 | C330021A05Rik | | 7.75 | | 0.82 | | 0.001493 | | 0.035 | |
| 134 | Cxcl14 | | 10.90 | | 0.82 | | 0.000403 | | 0.021 | |
| 135 | Hagh | | 8.71 | | 0.82 | | 0.000956 | | 0.029 | |
| 136 | Anxa5 | | 10.14 | | 0.82 | | 0.000532 | | 0.023 | |
| 137 | Mt1 | | 9.36 | | 0.82 | | 0.000725 | | 0.026 | |
| 138 | Cgrrf1 | | 12.80 | | 0.82 | | 0.000215 | | 0.017 | |
| 139 | Pigz | | 7.86 | | 0.81 | | 0.001418 | | 0.034 | |
| 140 | 1100001E04Rik | | 13.61 | | 0.81 | | 0.000169 | | 0.016 | |
| 141 | Tmem63a | | 8.49 | | 0.81 | | 0.001055 | | 0.030 | |
| 142 | Iscu | | 8.79 | | 0.81 | | 0.000924 | | 0.028 | |
| 143 | Brs3 | | 6.43 | | 0.81 | | 0.003013 | | 0.048 | |
| 144 | Ndrg4 | | 6.88 | | 0.80 | | 0.002333 | | 0.042 | |
| 145 | Snta1 | | 9.60 | | 0.80 | | 0.000659 | | 0.025 | |
| 146 | Cd59b | | 10.51 | | 0.80 | | 0.000464 | | 0.022 | |
| 147 | Amotl1 | | 9.01 | | 0.80 | | 0.000841 | | 0.027 | |
| 148 | Lysmd2 | | 10.49 | | 0.80 | | 0.000468 | | 0.022 | |
| 149 | Cox17 | | 6.66 | | 0.80 | | 0.002634 | | 0.045 | |
| 150 | Rph3a | | 10.46 | | 0.79 | | 0.000472 | | 0.022 | |
| 151 | Rhbdl1 | | 11.71 | | 0.79 | | 0.000304 | | 0.019 | |
| 152 | Pcdh20 | | 10.71 | | 0.79 | | 0.000431 | | 0.022 | |
| 153 | Myoc | | 6.37 | | 0.79 | | 0.003109 | | 0.049 | |
| 154 | Pdlim2 | | 34.17 | | 0.79 | | 4.38E-06 | | 0.005 | |
| 155 | Chn2 | | 7.36 | | 0.79 | | 0.001816 | | 0.038 | |
| 156 | Cpne6 | | 9.35 | | 0.79 | | 0.000729 | | 0.026 | |
| 157 | Tsc22d4 | | 11.82 | | 0.79 | | 0.000293 | | 0.018 | |
| 158 | Pacsin1 | | 7.13 | | 0.78 | | 0.002041 | | 0.040 | |
| 159 | Npb | | 9.15 | | 0.78 | | 0.000793 | | 0.027 | |
| 160 | Etl4 | | 12.31 | | 0.78 | | 0.00025 | | 0.017 | |
| 161 | Flywch1 | | 16.14 | | 0.78 | | 8.63E-05 | | 0.013 | |
| 162 | Ptprb | | 7.81 | | 0.77 | | 0.001451 | | 0.035 | |
| 163 | 2010004A03Rik | | 17.26 | | 0.77 | | 6.61E-05 | | 0.012 | |
| 164 | Cd74 | | 52.06 | | 0.77 | | 8.15E-07 | | 0.002 | |
| 165 | Hdc | | 8.89 | | 0.77 | | 0.000885 | | 0.028 | |
| 166 | Nhsl1 | | 6.56 | | 0.76 | | 0.002801 | | 0.047 | |
| 167 | Phyhip | | 7.81 | | 0.76 | | 0.001447 | | 0.035 | |
| 168 | Abcg1 | | 6.65 | | 0.76 | | 0.002656 | | 0.045 | |
| 169 | Hspa2 | | 7.74 | | 0.76 | | 0.001501 | | 0.035 | |
| 170 | Srxn1 | | 8.16 | | 0.76 | | 0.001228 | | 0.032 | |
| 171 | Acot11 | | 13.27 | | 0.76 | | 0.000187 | | 0.016 | |
| 172 | Higd1a | | 8.67 | | 0.75 | | 0.000972 | | 0.029 | |
| 173 | Rgs7bp | | 8.01 | | 0.74 | | 0.001319 | | 0.033 | |
| 174 | BC029169 | | 10.77 | | 0.74 | | 0.000422 | | 0.021 | |
| 175 | S100a13 | | 8.17 | | 0.74 | | 0.001219 | | 0.032 | |
| 176 | Ubl3 | | 11.13 | | 0.74 | | 0.000371 | | 0.020 | |
| 177 | Myh11 | | 10.17 | | 0.73 | | 0.000526 | | 0.023 | |
| 178 | Acsl6 | | 11.31 | | 0.73 | | 0.000349 | | 0.020 | |
| 179 | Impact | | 7.19 | | 0.73 | | 0.001981 | | 0.039 | |
| 180 | BC006662 | | 8.49 | | 0.72 | | 0.001055 | | 0.030 | |
| 181 | Gpx3 | | 9.63 | | 0.72 | | 0.000651 | | 0.025 | |
| 182 | Ctsf | | 7.20 | | 0.72 | | 0.001973 | | 0.039 | |
| 183 | Hhatl | | 8.72 | | 0.72 | | 0.000954 | | 0.029 | |
| 184 | Atp13a4 | | 7.95 | | 0.72 | | 0.001353 | | 0.034 | |
| 185 | Dgkg | | 15.52 | | 0.72 | | 0.000101 | | 0.013 | |
| 186 | Tmem56 | | 9.32 | | 0.72 | | 0.000738 | | 0.026 | |
| 187 | Lgals3bp | | 6.53 | | 0.72 | | 0.002834 | | 0.047 | |
| 188 | Apod | | 8.89 | | 0.72 | | 0.000886 | | 0.028 | |
| 189 | Hsd11b1 | | 7.98 | | 0.71 | | 0.001339 | | 0.033 | |
| 190 | Bhlhb2 | | 6.87 | | 0.71 | | 0.002348 | | 0.043 | |
| 191 | Igsf1 | | 21.57 | | 0.71 | | 2.73E-05 | | 0.009 | |
| 192 | Epb4.1l2 | | 14.68 | | 0.71 | | 0.000125 | | 0.014 | |
| 193 | Cyfip1 | | 8.80 | | 0.71 | | 0.000921 | | 0.028 | |
| 194 | Rasgrf1 | | 28.99 | | 0.71 | | 8.43E-06 | | 0.006 | |
| 195 | Atp2a1 | | 9.45 | | 0.71 | | 0.000698 | | 0.026 | |
| 196 | Prom1 | | 6.70 | | 0.71 | | 0.002576 | | 0.045 | |
| 197 | 4632428N05Rik | | 7.05 | | 0.70 | | 0.00214 | | 0.041 | |
| 198 | Tanc1 | | 10.11 | | 0.70 | | 0.000538 | | 0.023 | |
| 199 | Ninj2 | | 7.25 | | 0.70 | | 0.001918 | | 0.039 | |
| 200 | Tagln3 | | 10.43 | | 0.69 | | 0.000478 | | 0.022 | |
| 201 | Anxa6 | | 6.73 | | 0.69 | | 0.002542 | | 0.044 | |
| 202 | Ckmt1 | | 8.37 | | 0.69 | | 0.001115 | | 0.030 | |
| 203 | Syt12 | | 7.13 | | 0.69 | | 0.00205 | | 0.040 | |
| 204 | Cplx1 | | 8.65 | | 0.68 | | 0.000982 | | 0.029 | |
| 205 | Fn3k | | 8.54 | | 0.68 | | 0.001033 | | 0.030 | |
| 206 | Crhbp | | 9.04 | | 0.67 | | 0.000829 | | 0.027 | |
| 207 | Amigo2 | | 10.74 | | 0.67 | | 0.000426 | | 0.021 | |
| 208 | Plekhf1 | | 9.76 | | 0.67 | | 0.000617 | | 0.025 | |
| 209 | Bcap29 | | 6.64 | | 0.67 | | 0.002663 | | 0.045 | |
| 210 | B230342M21Rik | | 6.92 | | 0.67 | | 0.002289 | | 0.042 | |
| 211 | Nudt18 | | 6.57 | | 0.66 | | 0.002781 | | 0.047 | |
| 212 | Ipo8 | | 7.65 | | 0.66 | | 0.001567 | | 0.036 | |
| 213 | Tnnt1 | | 6.30 | | 0.66 | | 0.003242 | | 0.050 | |
| 214 | Ap2a1 | | 6.43 | | 0.66 | | 0.003005 | | 0.048 | |
| 215 | Pef1 | | 16.98 | | 0.66 | | 7.06E-05 | | 0.012 | |
| 216 | Tspyl2 | | 7.04 | | 0.66 | | 0.002142 | | 0.041 | |
| 217 | Ankrd56 | | 8.23 | | 0.66 | | 0.001191 | | 0.031 | |
| 218 | Rutbc2 | | 15.38 | | 0.66 | | 0.000104 | | 0.014 | |
| 219 | Dtd1 | | 15.70 | | 0.65 | | 9.61E-05 | | 0.013 | |
| 220 | Gabrg2 | | 7.13 | | 0.65 | | 0.002048 | | 0.040 | |
| 221 | Phkg1 | | 13.26 | | 0.65 | | 0.000187 | | 0.016 | |
| 222 | Plxdc1 | | 11.86 | | 0.65 | | 0.00029 | | 0.018 | |
| 223 | Krt222 | | 9.70 | | 0.65 | | 0.000631 | | 0.025 | |
| 224 | Fbxl16 | | 9.04 | | 0.64 | | 0.00083 | | 0.027 | |
| 225 | Atp6v1b2 | | 7.27 | | 0.64 | | 0.001902 | | 0.039 | |
| 226 | Tmem38a | | 16.01 | | 0.64 | | 8.91E-05 | | 0.013 | |
| 227 | P2ry14 | | 8.76 | | 0.63 | | 0.000935 | | 0.028 | |
| 228 | Gabarapl1 | | 9.00 | | 0.63 | | 0.000842 | | 0.027 | |
| 229 | Slc6a11 | | 8.49 | | 0.63 | | 0.001056 | | 0.030 | |
| 230 | Vamp4 | | 9.15 | | 0.63 | | 0.000793 | | 0.027 | |
| 231 | Msrb2 | | 32.23 | | 0.63 | | 5.53E-06 | | 0.005 | |
| 232 | Dnaic1 | | 9.91 | | 0.63 | | 0.000583 | | 0.024 | |
| 233 | Stard13 | | 6.43 | | 0.63 | | 0.003002 | | 0.048 | |
| 234 | Ifitm3 | | 8.13 | | 0.62 | | 0.001245 | | 0.032 | |
| 235 | Ttc7b | | 6.56 | | 0.62 | | 0.002786 | | 0.047 | |
| 236 | Rerg | | 6.74 | | 0.62 | | 0.002525 | | 0.044 | |
| 237 | Plekha2 | | 6.98 | | 0.62 | | 0.002214 | | 0.042 | |
| 238 | Nptn | | 6.66 | | 0.62 | | 0.002647 | | 0.045 | |
| 239 | 2410187C16Rik | | 16.38 | | 0.62 | | 8.13E-05 | | 0.013 | |
| 240 | Abhd12 | | 9.85 | | 0.62 | | 0.000596 | | 0.024 | |
| 241 | Cd83 | | 7.63 | | 0.62 | | 0.001582 | | 0.036 | |
| 242 | A630054L15Rik | | 6.70 | | 0.61 | | 0.002577 | | 0.045 | |
| 243 | Pik3r4 | | 10.54 | | 0.61 | | 0.000459 | | 0.022 | |
| 244 | Inpp5a | | 14.79 | | 0.61 | | 0.000122 | | 0.014 | |
| 245 | Slc5a5 | | 11.63 | | 0.61 | | 0.000313 | | 0.019 | |
| 246 | 5730472N09Rik | | 10.93 | | 0.61 | | 0.000398 | | 0.021 | |
| 247 | Cpeb1 | | 9.90 | | 0.61 | | 0.000584 | | 0.024 | |
| 248 | Myrip | | 15.80 | | 0.61 | | 9.37E-05 | | 0.013 | |
| 249 | Gpc5 | | 8.44 | | 0.61 | | 0.00108 | | 0.030 | |
| 250 | Plekhb1 | | 11.97 | | 0.60 | | 0.000279 | | 0.018 | |
| 251 | Itm2c | | 10.91 | | 0.60 | | 0.000401 | | 0.021 | |
| 252 | Cadps2 | | 10.39 | | 0.60 | | 0.000485 | | 0.022 | |
| 253 | Pop5 | | 7.53 | | 0.60 | | 0.001669 | | 0.037 | |
| 254 | Gmpr | | 6.83 | | 0.59 | | 0.002398 | | 0.043 | |
| 255 | 6430573F11Rik | | 8.30 | | 0.59 | | 0.001151 | | 0.031 | |
| 256 | Rhbdd1 | | 8.29 | | 0.59 | | 0.001156 | | 0.031 | |
| 257 | Syngr1 | | 9.48 | | 0.59 | | 0.000692 | | 0.026 | |
| 258 | Gpr103 | | 6.37 | | 0.58 | | 0.003119 | | 0.049 | |
| 259 | Creld1 | | 14.99 | | 0.58 | | 0.000115 | | 0.014 | |
| 260 | Cml4 | | 15.94 | | 0.58 | | 9.06E-05 | | 0.013 | |
| 261 | Rassf5 | | 7.33 | | 0.58 | | 0.00184 | | 0.038 | |
| 262 | Ets2 | | 7.04 | | 0.58 | | 0.002147 | | 0.041 | |
| 263 | Elovl1 | | 7.51 | | 0.58 | | 0.001684 | | 0.037 | |
| 264 | Dexi | | 11.22 | | 0.57 | | 0.000359 | | 0.020 | |
| 265 | Ramp1 | | 9.13 | | 0.57 | | 0.000798 | | 0.027 | |
| 266 | Pnpo | | 11.64 | | 0.57 | | 0.000312 | | 0.019 | |
| 267 | Hprt1 | | 6.70 | | 0.57 | | 0.002588 | | 0.045 | |
| 268 | Slc1a2 | | 6.41 | | 0.57 | | 0.003049 | | 0.048 | |
| 269 | Resp18 | | 17.18 | | 0.57 | | 6.74E-05 | | 0.012 | |
| 270 | Extl2 | | 9.05 | | 0.57 | | 0.000827 | | 0.027 | |
| 271 | Hspa9 | | 6.79 | | 0.57 | | 0.002461 | | 0.044 | |
| 272 | Cd164 | | 8.38 | | 0.57 | | 0.001108 | | 0.030 | |
| 273 | 4922503N01Rik | | 7.34 | | 0.57 | | 0.001833 | | 0.038 | |
| 274 | Tpm2 | | 7.21 | | 0.57 | | 0.001959 | | 0.039 | |
| 275 | 2810439F02Rik | | 9.62 | | 0.56 | | 0.000654 | | 0.025 | |
| 276 | Trim54 | | 7.78 | | 0.56 | | 0.001471 | | 0.035 | |
| 277 | Camk2a | | 7.23 | | 0.56 | | 0.001942 | | 0.039 | |
| 278 | Galntl1 | | 8.37 | | 0.56 | | 0.001113 | | 0.030 | |
| 279 | Clu | | 6.77 | | 0.56 | | 0.002488 | | 0.044 | |
| 280 | Timm17a | | 7.19 | | 0.55 | | 0.001979 | | 0.039 | |
| 281 | Sqstm1 | | 20.38 | | 0.55 | | 3.42E-05 | | 0.010 | |
| 282 | Lrrc57 | | 6.59 | | 0.55 | | 0.002753 | | 0.046 | |
| 283 | Scg2 | | 19.38 | | 0.55 | | 4.18E-05 | | 0.011 | |
| 284 | Stip1 | | 11.20 | | 0.54 | | 0.000362 | | 0.020 | |
| 285 | Gadd45a | | 6.99 | | 0.54 | | 0.002203 | | 0.042 | |
| 286 | 0610011F06Rik | | 26.06 | | 0.54 | | 1.29E-05 | | 0.008 | |
| 287 | A030007L17Rik | | 6.73 | | 0.54 | | 0.002533 | | 0.044 | |
| 288 | Paqr7 | | 7.21 | | 0.54 | | 0.001965 | | 0.039 | |
| 289 | Plekhb2 | | 6.41 | | 0.54 | | 0.003049 | | 0.048 | |
| 290 | Mdh1 | | 15.13 | | 0.53 | | 0.000111 | | 0.014 | |
| 291 | Stim1 | | 13.37 | | 0.53 | | 0.000181 | | 0.016 | |
| 292 | Nr1d1 | | 7.43 | | 0.53 | | 0.001756 | | 0.037 | |
| 293 | Aqp4 | | 6.45 | | 0.53 | | 0.002978 | | 0.048 | |
| 294 | 2310016M24Rik | | 8.86 | | 0.53 | | 0.000896 | | 0.028 | |
| 295 | 6330406I15Rik | | 7.18 | | 0.53 | | 0.001989 | | 0.039 | |
| 296 | Scrg1 | | 10.79 | | 0.53 | | 0.000418 | | 0.021 | |
| 297 | Gsn | | 6.35 | | 0.52 | | 0.003144 | | 0.049 | |
| 298 | 1110049F12Rik | | 8.89 | | 0.52 | | 0.000886 | | 0.028 | |
| 299 | Arf3 | | 8.15 | | 0.52 | | 0.001233 | | 0.032 | |
| 300 | Ihpk1 | | 10.03 | | 0.52 | | 0.000556 | | 0.023 | |
| 301 | Dio2 | | 6.58 | | 0.52 | | 0.002763 | | 0.046 | |
| 302 | Iqsec3 | | 7.45 | | 0.52 | | 0.001734 | | 0.037 | |
| 303 | Txnl1 | | 6.97 | | 0.52 | | 0.00223 | | 0.042 | |
| 304 | Lman2 | | 9.43 | | 0.51 | | 0.000706 | | 0.026 | |
| 305 | Syn2 | | 10.39 | | 0.51 | | 0.000485 | | 0.022 | |
| 306 | Peg3 | | 7.92 | | 0.51 | | 0.001374 | | 0.034 | |
| 307 | Gsbs | | 7.39 | | 0.51 | | 0.001792 | | 0.038 | |
| 308 | Rab6ip1 | | 11.53 | | 0.50 | | 0.000323 | | 0.019 | |
| 309 | Slc25a44 | | 10.23 | | 0.50 | | 0.000515 | | 0.023 | |
| 310 | Tmem33 | | 6.85 | | 0.50 | | 0.002372 | | 0.043 | |
| 311 | Ttc33 | | 10.46 | | 0.50 | | 0.000472 | | 0.022 | |
| 312 | Ypel3 | | 17.27 | | 0.50 | | 6.6E-05 | | 0.012 | |
| 313 | Atp6v1c1 | | 13.69 | | 0.49 | | 0.000165 | | 0.016 | |
| 314 | B230208H17Rik | | 9.26 | | 0.49 | | 0.000757 | | 0.026 | |
| 315 | Hdhd2 | | 15.05 | | 0.49 | | 0.000113 | | 0.014 | |
| 316 | Ltc4s | | 9.04 | | 0.49 | | 0.000831 | | 0.027 | |
| 317 | Becn1 | | 7.16 | | 0.49 | | 0.002013 | | 0.039 | |
| 318 | Mrpl4 | | 7.46 | | 0.48 | | 0.001722 | | 0.037 | |
| 319 | D4Bwg0951e | | 10.57 | | 0.48 | | 0.000453 | | 0.022 | |
| 320 | Ogdh | | 8.12 | | 0.48 | | 0.001248 | | 0.032 | |
| 321 | 6332401O19Rik | | 10.20 | | 0.48 | | 0.00052 | | 0.023 | |
| 322 | Ephx1 | | 9.15 | | 0.48 | | 0.000793 | | 0.027 | |
| 323 | Thrsp | | 9.04 | | 0.47 | | 0.00083 | | 0.027 | |
| 324 | Cox11 | | 7.30 | | 0.47 | | 0.001874 | | 0.039 | |
| 325 | Serpinf1 | | 10.69 | | 0.47 | | 0.000434 | | 0.022 | |
| 326 | Txndc4 | | 7.32 | | 0.47 | | 0.001851 | | 0.039 | |
| 327 | AU040829 | | 11.76 | | 0.47 | | 0.000299 | | 0.018 | |
| 328 | Laptm4a | | 8.86 | | 0.46 | | 0.000897 | | 0.028 | |
| 329 | Ppp1r3f | | 17.61 | | 0.46 | | 6.11E-05 | | 0.012 | |
| 330 | Inppl1 | | 9.38 | | 0.46 | | 0.000719 | | 0.026 | |
| 331 | Pld1 | | 6.37 | | 0.46 | | 0.003121 | | 0.049 | |
| 332 | Prkar1a | | 6.57 | | 0.46 | | 0.002782 | | 0.047 | |
| 333 | Tspan3 | | 11.80 | | 0.46 | | 0.000295 | | 0.018 | |
| 334 | Gpr123 | | 16.69 | | 0.46 | | 7.55E-05 | | 0.013 | |
| 335 | Sh3bp5l | | 6.72 | | 0.45 | | 0.00255 | | 0.044 | |
| 336 | Tmem126b | | 7.77 | | 0.45 | | 0.001481 | | 0.035 | |
| 337 | 4921511K06Rik | | 13.58 | | 0.45 | | 0.00017 | | 0.016 | |
| 338 | Kcnab1 | | 7.31 | | 0.45 | | 0.001865 | | 0.039 | |
| 339 | 2310011J03Rik | | 10.69 | | 0.45 | | 0.000433 | | 0.022 | |
| 340 | Snph | | 7.08 | | 0.44 | | 0.002101 | | 0.040 | |
| 341 | LOC641240 | | 7.96 | | 0.44 | | 0.001351 | | 0.034 | |
| 342 | M6pr | | 13.15 | | 0.44 | | 0.000193 | | 0.017 | |
| 343 | Urod | | 12.29 | | 0.44 | | 0.000252 | | 0.017 | |
| 344 | Uchl5 | | 9.18 | | 0.44 | | 0.000781 | | 0.026 | |
| 345 | Actr8 | | 8.04 | | 0.44 | | 0.001302 | | 0.033 | |
| 346 | Pnpla7 | | 22.00 | | 0.44 | | 2.53E-05 | | 0.009 | |
| 347 | Ppp1r14a | | 7.60 | | 0.44 | | 0.001608 | | 0.036 | |
| 348 | Itih2 | | 9.44 | | 0.44 | | 0.000702 | | 0.026 | |
| 349 | Lgals8 | | 8.77 | | 0.43 | | 0.000932 | | 0.028 | |
| 350 | Egr4 | | 7.26 | | 0.43 | | 0.001912 | | 0.039 | |
| 351 | Mgll | | 20.23 | | 0.43 | | 3.52E-05 | | 0.010 | |
| 352 | 4732418C07Rik | | 6.59 | | 0.43 | | 0.002753 | | 0.046 | |
| 353 | Slc24a3 | | 57.51 | | 0.42 | | 5.47E-07 | | 0.002 | |
| 354 | Slc2a3 | | 7.37 | | 0.42 | | 0.001803 | | 0.038 | |
| 355 | Osbpl6 | | 10.46 | | 0.42 | | 0.000473 | | 0.022 | |
| 356 | Ptplb | | 7.94 | | 0.42 | | 0.00136 | | 0.034 | |
| 357 | Sharpin | | 7.61 | | 0.42 | | 0.001601 | | 0.036 | |
| 358 | Antxr1 | | 8.22 | | 0.42 | | 0.001195 | | 0.031 | |
| 359 | Ghr | | 8.83 | | 0.42 | | 0.000909 | | 0.028 | |
| 360 | Ppt1 | | 12.49 | | 0.42 | | 0.000237 | | 0.017 | |
| 361 | Lcat | | 9.38 | | 0.42 | | 0.000719 | | 0.026 | |
| 362 | Nudt9 | | 7.84 | | 0.41 | | 0.001431 | | 0.034 | |
| 363 | Tmem25 | | 8.93 | | 0.41 | | 0.000868 | | 0.027 | |
| 364 | 6330409N04Rik | | 7.22 | | 0.41 | | 0.001953 | | 0.039 | |
| 365 | Gprasp2 | | 14.32 | | 0.41 | | 0.000138 | | 0.015 | |
| 366 | Tnnt2 | | 6.91 | | 0.41 | | 0.002296 | | 0.042 | |
| 367 | Kcnab3 | | 10.92 | | 0.41 | | 0.0004 | | 0.021 | |
| 368 | 2310061C15Rik | | 6.58 | | 0.40 | | 0.00276 | | 0.046 | |
| 369 | H2-Eb1 | | 20.34 | | 0.40 | | 3.45E-05 | | 0.010 | |
| 370 | Nme3 | | 10.57 | | 0.40 | | 0.000453 | | 0.022 | |
| 371 | Bag2 | | 6.54 | | 0.40 | | 0.002829 | | 0.047 | |
| 372 | Tef | | 7.15 | | 0.40 | | 0.002026 | | 0.039 | |
| 373 | St8sia5 | | 9.49 | | 0.40 | | 0.000689 | | 0.026 | |
| 374 | Jam3 | | 7.52 | | 0.40 | | 0.001675 | | 0.037 | |
| 375 | Aco2 | | 7.98 | | 0.39 | | 0.001339 | | 0.033 | |
| 376 | Tmed4 | | 17.08 | | 0.39 | | 6.9E-05 | | 0.012 | |
| 377 | Atxn1 | | 16.26 | | 0.39 | | 8.38E-05 | | 0.013 | |
| 378 | Sh3gl2 | | 8.32 | | 0.39 | | 0.001139 | | 0.031 | |
| 379 | Ube2q2 | | 7.31 | | 0.38 | | 0.001865 | | 0.039 | |
| 380 | Fastkd3 | | 6.90 | | 0.38 | | 0.002319 | | 0.042 | |
| 381 | 4933407N01Rik | | 7.16 | | 0.37 | | 0.002012 | | 0.039 | |
| 382 | Flt1 | | 6.94 | | 0.37 | | 0.002262 | | 0.042 | |
| 383 | Pdxp | | 14.58 | | 0.37 | | 0.000129 | | 0.014 | |
| 384 | Acbd4 | | 8.69 | | 0.37 | | 0.000964 | | 0.029 | |
| 385 | Aip | | 8.56 | | 0.37 | | 0.001024 | | 0.030 | |
| 386 | Apba3 | | 12.13 | | 0.36 | | 0.000265 | | 0.018 | |
| 387 | Acp6 | | 8.96 | | 0.36 | | 0.000857 | | 0.027 | |
| 388 | Kcnj9 | | 7.32 | | 0.36 | | 0.001852 | | 0.039 | |
| 389 | 1700012G19Rik | | 9.59 | | 0.36 | | 0.00066 | | 0.025 | |
| 390 | Herc3 | | 7.15 | | 0.36 | | 0.002026 | | 0.039 | |
| 391 | AW049604 | | 8.16 | | 0.36 | | 0.001226 | | 0.032 | |
| 392 | Lman2l | | 6.99 | | 0.35 | | 0.002205 | | 0.042 | |
| 393 | LOC638935 | | 9.38 | | 0.35 | | 0.000721 | | 0.026 | |
| 394 | Ppp1r11 | | 8.99 | | 0.35 | | 0.000846 | | 0.027 | |
| 395 | Sod2 | | 6.46 | | 0.35 | | 0.002961 | | 0.048 | |
| 396 | 5830472M02Rik | | 7.44 | | 0.35 | | 0.001746 | | 0.037 | |
| 397 | 1300010F03Rik | | 10.53 | | 0.35 | | 0.00046 | | 0.022 | |
| 398 | Stx4a | | 6.75 | | 0.35 | | 0.002513 | | 0.044 | |
| 399 | BC060631 | | 12.21 | | 0.35 | | 0.000258 | | 0.018 | |
| 400 | Cryge | | 8.11 | | 0.35 | | 0.001259 | | 0.032 | |
| 401 | 2010005J08Rik | | 7.64 | | 0.34 | | 0.001576 | | 0.036 | |
| 402 | Nol3 | | 7.63 | | 0.34 | | 0.001584 | | 0.036 | |
| 403 | Igtp | | 7.68 | | 0.34 | | 0.001549 | | 0.036 | |
| 404 | Trak2 | | 8.57 | | 0.34 | | 0.00102 | | 0.030 | |
| 405 | Pja2 | | 6.66 | | 0.34 | | 0.002644 | | 0.045 | |
| 406 | Ccdc44 | | 7.02 | | 0.34 | | 0.002165 | | 0.041 | |
| 407 | Mypn | | 8.29 | | 0.34 | | 0.001156 | | 0.031 | |
| 408 | Trpm2 | | 12.66 | | 0.33 | | 0.000224 | | 0.017 | |
| 409 | Tmem19 | | 7.77 | | 0.33 | | 0.001481 | | 0.035 | |
| 410 | Zfp691 | | 13.04 | | 0.33 | | 0.0002 | | 0.017 | |
| 411 | Ptrh1 | | 7.53 | | 0.33 | | 0.001669 | | 0.037 | |
| 412 | Ube2f | | 22.37 | | 0.33 | | 2.37E-05 | | 0.009 | |
| 413 | OTTMUSG00000007655 | | 8.77 | | 0.32 | | 0.000932 | | 0.028 | |
| 414 | Fundc1 | | 6.46 | | 0.32 | | 0.00295 | | 0.048 | |
| 415 | Nde1 | | 14.98 | | 0.32 | | 0.000116 | | 0.014 | |
| 416 | Phka1 | | 7.03 | | 0.32 | | 0.002157 | | 0.041 | |
| 417 | Rasgef1b | | 7.30 | | 0.32 | | 0.001872 | | 0.039 | |
| 418 | Slc25a37 | | 7.80 | | 0.32 | | 0.00146 | | 0.035 | |
| 419 | Zfp37 | | 6.73 | | 0.32 | | 0.002538 | | 0.044 | |
| 420 | 2010003K11Rik | | 6.50 | | 0.32 | | 0.002889 | | 0.047 | |
| 421 | Tsr2 | | 8.50 | | 0.31 | | 0.00105 | | 0.030 | |
| 422 | Rgs17 | | 8.44 | | 0.31 | | 0.001082 | | 0.030 | |
| 423 | Ly6g6d | 12.45 | | 0.31 | | 0.000239 | | 0.017 | |  |
| 424 | Hdac4 | | 6.32 | | 0.31 | | 0.003213 | | 0.050 | |
| 425 | Adora2b | | 9.84 | | 0.31 | | 0.000599 | | 0.024 | |
| 426 | Mkx | | 8.53 | | 0.31 | | 0.001035 | | 0.030 | |
| 427 | Acp2 | | 7.63 | | 0.31 | | 0.001584 | | 0.036 | |
| 428 | Ddx1 | | 12.12 | | 0.31 | | 0.000266 | | 0.018 | |
| 429 | Tmem93 | | 7.95 | | 0.31 | | 0.001355 | | 0.034 | |
| 430 | Epo | | 11.48 | | 0.30 | | 0.000329 | | 0.019 | |
| 431 | Luzp1 | | 6.61 | | 0.30 | | 0.002717 | | 0.046 | |

| Comp6 | Symbol | t-value | fold_change | p-value | BH-FDR |
| --- | --- | --- | --- | --- | --- |
| 1 | St8sia2 | -15.63 | -2.77 | 9.79E-05 | 0.013 |
| 2 | Dpysl3 | -52.47 | -2.07 | 7.9E-07 | 0.002 |
| 3 | Akr1c19 | -44.50 | -1.81 | 1.53E-06 | 0.002 |
| 4 | 2410146L05Rik | -15.72 | -1.74 | 9.58E-05 | 0.013 |
| 5 | Sbk1 | -29.51 | -1.70 | 7.85E-06 | 0.006 |
| 6 | Dpysl5 | -7.43 | -1.69 | 0.001753 | 0.037 |
| 7 | Cdkn1a | -11.44 | -1.56 | 0.000334 | 0.019 |
| 8 | Pafah1b3 | -15.35 | -1.56 | 0.000105 | 0.014 |
| 9 | Casp3 | -19.41 | -1.54 | 4.15E-05 | 0.011 |
| 10 | Bcl11b | -10.38 | -1.54 | 0.000487 | 0.022 |
| 11 | Vash2 | -15.18 | -1.51 | 0.00011 | 0.014 |
| 12 | Rab3d | -23.56 | -1.47 | 1.92E-05 | 0.008 |
| 13 | Smpd3 | -15.75 | -1.45 | 9.48E-05 | 0.013 |
| 14 | B3gnt5 | -7.31 | -1.44 | 0.001863 | 0.039 |
| 15 | Sh3bp2 | -22.29 | -1.41 | 2.4E-05 | 0.009 |
| 16 | Ddah2 | -6.51 | -1.39 | 0.00288 | 0.047 |
| 17 | 2310003H01Rik | -6.93 | -1.31 | 0.002278 | 0.042 |
| 18 | Klf7 | -7.15 | -1.29 | 0.002026 | 0.039 |
| 19 | Ypel1 | -34.84 | -1.28 | 4.05E-06 | 0.005 |
| 20 | Lefty2 | -30.26 | -1.28 | 7.11E-06 | 0.006 |
| 21 | Zbtb12 | -7.47 | -1.24 | 0.001717 | 0.037 |
| 22 | Epha8 | -12.27 | -1.24 | 0.000253 | 0.017 |
| 23 | Stag3 | -11.41 | -1.24 | 0.000337 | 0.019 |
| 24 | Bzw2 | -14.16 | -1.23 | 0.000144 | 0.015 |
| 25 | Pcdh21 | -8.61 | -1.22 | 0.000999 | 0.029 |
| 26 | Rac3 | -11.50 | -1.21 | 0.000327 | 0.019 |
| 27 | Dkkl1 | -13.49 | -1.21 | 0.000175 | 0.016 |
| 28 | Hn1 | -6.58 | -1.20 | 0.002764 | 0.046 |
| 29 | 4930519N16Rik | -22.76 | -1.18 | 2.21E-05 | 0.009 |
| 30 | Zbtb5 | -16.17 | -1.16 | 8.55E-05 | 0.013 |
| 31 | Gpc2 | -15.23 | -1.14 | 0.000108 | 0.014 |
| 32 | 9030425E11Rik | -22.88 | -1.14 | 2.16E-05 | 0.009 |
| 33 | Ccdc120 | -12.33 | -1.13 | 0.000249 | 0.017 |
| 34 | Sct | -16.57 | -1.10 | 7.76E-05 | 0.013 |
| 35 | Ccnu | -9.12 | -1.09 | 0.000802 | 0.027 |
| 36 | Bcl11a | -10.49 | -1.08 | 0.000467 | 0.022 |
| 37 | Arrdc4 | -15.40 | -1.08 | 0.000104 | 0.014 |
| 38 | P2rx2 | -6.53 | -1.07 | 0.002837 | 0.047 |
| 39 | Prox1 | -12.83 | -1.05 | 0.000213 | 0.017 |
| 40 | Dok4 | -14.49 | -1.04 | 0.000132 | 0.014 |
| 41 | 3110047P20Rik | -13.25 | -1.03 | 0.000187 | 0.016 |
| 42 | Dctd | -14.76 | -1.03 | 0.000123 | 0.014 |
| 43 | Lgals3 | -7.80 | -1.02 | 0.001459 | 0.035 |
| 44 | Nuak2 | -14.02 | -1.01 | 0.00015 | 0.015 |
| 45 | Aloxe3 | -10.47 | -1.01 | 0.000471 | 0.022 |
| 46 | Stac | -13.05 | -1.00 | 0.000199 | 0.017 |
| 47 | 1110038B12Rik | -11.76 | -0.99 | 0.000299 | 0.018 |
| 48 | Lck | -13.58 | -0.98 | 0.00017 | 0.016 |
| 49 | Gbx2 | -6.99 | -0.98 | 0.002201 | 0.042 |
| 50 | 2310007A19Rik | -8.98 | -0.98 | 0.000851 | 0.027 |
| 51 | D130058I21Rik | -11.49 | -0.98 | 0.000328 | 0.019 |
| 52 | Csrp2 | -28.03 | -0.98 | 9.64E-06 | 0.007 |
| 53 | BC065085 | -10.94 | -0.97 | 0.000397 | 0.021 |
| 54 | Igf2bp3 | -12.31 | -0.97 | 0.00025 | 0.017 |
| 55 | 2810046M22Rik | -8.43 | -0.97 | 0.001086 | 0.030 |
| 56 | Mcm5 | -7.41 | -0.96 | 0.001771 | 0.038 |
| 57 | Tesk1 | -10.76 | -0.96 | 0.000423 | 0.021 |
| 58 | Gpr21 | -11.59 | -0.96 | 0.000317 | 0.019 |
| 59 | Lrrc23 | -9.86 | -0.96 | 0.000595 | 0.024 |
| 60 | Ccdc23 | -14.63 | -0.96 | 0.000127 | 0.014 |
| 61 | Tead2 | -12.17 | -0.94 | 0.000262 | 0.018 |
| 62 | Chst7 | -8.48 | -0.94 | 0.001059 | 0.030 |
| 63 | Efnb1 | -9.92 | -0.93 | 0.000579 | 0.024 |
| 64 | Slc26a1 | -10.87 | -0.93 | 0.000407 | 0.021 |
| 65 | Nans | -16.84 | -0.92 | 7.3E-05 | 0.013 |
| 66 | Pdrg1 | -10.53 | -0.91 | 0.00046 | 0.022 |
| 67 | Rasip1 | -9.31 | -0.91 | 0.000741 | 0.026 |
| 68 | Rpl22 | -10.89 | -0.91 | 0.000404 | 0.021 |
| 69 | 4632417K18Rik | -13.90 | -0.91 | 0.000155 | 0.015 |
| 70 | Fgd2 | -8.92 | -0.90 | 0.000874 | 0.028 |
| 71 | Tdrd6 | -9.25 | -0.90 | 0.000759 | 0.026 |
| 72 | Emid2 | -13.97 | -0.89 | 0.000152 | 0.015 |
| 73 | 2810003C17Rik | -13.82 | -0.88 | 0.000159 | 0.015 |
| 74 | Amn | -6.42 | -0.87 | 0.003032 | 0.048 |
| 75 | Igf2bp2 | -14.06 | -0.87 | 0.000149 | 0.015 |
| 76 | C1qtnf2 | -12.53 | -0.87 | 0.000233 | 0.017 |
| 77 | Sh3yl1 | -9.89 | -0.86 | 0.000586 | 0.024 |
| 78 | 2010310D06Rik | -22.01 | -0.86 | 2.52E-05 | 0.009 |
| 79 | Nkd2 | -10.61 | -0.85 | 0.000447 | 0.022 |
| 80 | Cenpa | -6.88 | -0.85 | 0.002344 | 0.043 |
| 81 | Ykt6 | -11.40 | -0.85 | 0.000338 | 0.019 |
| 82 | Tsga2 | -7.04 | -0.84 | 0.002149 | 0.041 |
| 83 | Rala | -7.95 | -0.83 | 0.001357 | 0.034 |
| 84 | 2310057J16Rik | -15.18 | -0.83 | 0.00011 | 0.014 |
| 85 | Klk8 | -7.90 | -0.82 | 0.001386 | 0.034 |
| 86 | Dcx | -33.42 | -0.82 | 4.78E-06 | 0.005 |
| 87 | Fbp2 | -10.96 | -0.82 | 0.000394 | 0.021 |
| 88 | Psme2 | -13.12 | -0.81 | 0.000195 | 0.017 |
| 89 | Cdkn1c | -10.31 | -0.81 | 0.000499 | 0.022 |
| 90 | Fign | -6.56 | -0.80 | 0.002796 | 0.047 |
| 91 | Triobp | -11.24 | -0.80 | 0.000357 | 0.020 |
| 92 | Prep | -7.27 | -0.80 | 0.001901 | 0.039 |
| 93 | Gipc2 | -7.95 | -0.80 | 0.001359 | 0.034 |
| 94 | Ceecam1 | -18.02 | -0.80 | 5.57E-05 | 0.012 |
| 95 | Agrn | -24.15 | -0.79 | 1.74E-05 | 0.008 |
| 96 | 1500010J02Rik | -8.38 | -0.79 | 0.001111 | 0.030 |
| 97 | Igsf9 | -11.02 | -0.79 | 0.000385 | 0.021 |
| 98 | Dbn1 | -6.93 | -0.78 | 0.002281 | 0.042 |
| 99 | Slc29a4 | -10.46 | -0.78 | 0.000472 | 0.022 |
| 100 | Cish | -12.17 | -0.78 | 0.000261 | 0.018 |
| 101 | Kif23 | -20.36 | -0.78 | 3.44E-05 | 0.010 |
| 102 | Casp7 | -17.18 | -0.78 | 6.73E-05 | 0.012 |
| 103 | 9630031F12Rik | -14.03 | -0.77 | 0.00015 | 0.015 |
| 104 | Map3k6 | -10.09 | -0.77 | 0.000544 | 0.023 |
| 105 | Hsd11b2 | -6.50 | -0.77 | 0.00289 | 0.047 |
| 106 | Rtn2 | -6.30 | -0.77 | 0.003245 | 0.050 |
| 107 | Krt19 | -10.98 | -0.76 | 0.000391 | 0.021 |
| 108 | Nr2f1 | -6.49 | -0.76 | 0.002901 | 0.047 |
| 109 | Nme6 | -12.64 | -0.76 | 0.000226 | 0.017 |
| 110 | Mthfd2 | -24.27 | -0.75 | 1.71E-05 | 0.008 |
| 111 | Agtrl1 | -11.57 | -0.75 | 0.000319 | 0.019 |
| 112 | Slc38a5 | -31.46 | -0.75 | 6.08E-06 | 0.005 |
| 113 | Gdpd5 | -9.26 | -0.75 | 0.000755 | 0.026 |
| 114 | Dnahc2 | -10.08 | -0.75 | 0.000545 | 0.023 |
| 115 | AW146242 | -16.12 | -0.74 | 8.66E-05 | 0.013 |
| 116 | Fchsd1 | -8.76 | -0.74 | 0.000936 | 0.028 |
| 117 | Tes | -23.03 | -0.74 | 2.11E-05 | 0.009 |
| 118 | Zbtb8 | -7.87 | -0.74 | 0.001411 | 0.034 |
| 119 | Pif1 | -7.25 | -0.74 | 0.001923 | 0.039 |
| 120 | Pih1d1 | -12.99 | -0.74 | 0.000203 | 0.017 |
| 121 | Tacstd2 | -20.93 | -0.74 | 3.08E-05 | 0.009 |
| 122 | Slc14a2 | -9.95 | -0.73 | 0.000573 | 0.024 |
| 123 | 4930519N13Rik | -12.14 | -0.73 | 0.000264 | 0.018 |
| 124 | Bcnp1 | -17.18 | -0.73 | 6.74E-05 | 0.012 |
| 125 | Lypd3 | -35.67 | -0.73 | 3.69E-06 | 0.005 |
| 126 | Ankrd13b | -55.87 | -0.73 | 6.15E-07 | 0.002 |
| 127 | Prr7 | -7.56 | -0.73 | 0.001637 | 0.037 |
| 128 | Tubb3 | -7.53 | -0.73 | 0.001664 | 0.037 |
| 129 | Chrna3 | -9.20 | -0.73 | 0.000775 | 0.026 |
| 130 | Hyal2 | -7.51 | -0.73 | 0.001683 | 0.037 |
| 131 | Top2a | -15.23 | -0.72 | 0.000108 | 0.014 |
| 132 | Crcp | -8.01 | -0.72 | 0.001316 | 0.033 |
| 133 | BC052040 | -9.88 | -0.72 | 0.000588 | 0.024 |
| 134 | Hsdl1 | -57.52 | -0.72 | 5.47E-07 | 0.002 |
| 135 | Acat2 | -10.63 | -0.72 | 0.000443 | 0.022 |
| 136 | Dusp2 | -6.42 | -0.71 | 0.00302 | 0.048 |
| 137 | Rev3l | -12.84 | -0.71 | 0.000212 | 0.017 |
| 138 | Zfp41 | -12.39 | -0.71 | 0.000244 | 0.017 |
| 139 | H2-DMa | -8.39 | -0.71 | 0.001104 | 0.030 |
| 140 | Zfp90 | -6.39 | -0.71 | 0.003072 | 0.049 |
| 141 | Cenpt | -12.82 | -0.70 | 0.000214 | 0.017 |
| 142 | Itpkc | -18.67 | -0.70 | 4.85E-05 | 0.012 |
| 143 | Tubb5 | -10.05 | -0.70 | 0.000552 | 0.023 |
| 144 | Arv1 | -7.62 | -0.70 | 0.001594 | 0.036 |
| 145 | Zfp202 | -6.56 | -0.69 | 0.002799 | 0.047 |
| 146 | Ppp1r1a | -29.91 | -0.69 | 7.44E-06 | 0.006 |
| 147 | C030003D03Rik | -6.71 | -0.69 | 0.002564 | 0.045 |
| 148 | 4933433K01Rik | -10.82 | -0.69 | 0.000413 | 0.021 |
| 149 | Bnip1 | -11.39 | -0.69 | 0.000339 | 0.019 |
| 150 | Eif2s2 | -10.78 | -0.69 | 0.00042 | 0.021 |
| 151 | Zfp282 | -7.72 | -0.68 | 0.001513 | 0.035 |
| 152 | Basp1 | -7.13 | -0.68 | 0.00205 | 0.040 |
| 153 | Ankfy1 | -6.79 | -0.68 | 0.002463 | 0.044 |
| 154 | Maged2 | -8.67 | -0.67 | 0.000973 | 0.029 |
| 155 | Zkscan17 | -11.59 | -0.67 | 0.000317 | 0.019 |
| 156 | Klf6 | -13.55 | -0.67 | 0.000172 | 0.016 |
| 157 | Mfap3 | -6.65 | -0.66 | 0.002661 | 0.045 |
| 158 | Pold1 | -7.37 | -0.66 | 0.001805 | 0.038 |
| 159 | Kif2c | -7.58 | -0.66 | 0.001622 | 0.036 |
| 160 | Palld | -8.50 | -0.66 | 0.001051 | 0.030 |
| 161 | Ibrdc3 | -8.81 | -0.66 | 0.000918 | 0.028 |
| 162 | Pold3 | -8.74 | -0.66 | 0.000946 | 0.029 |
| 163 | Fbxo46 | -14.00 | -0.66 | 0.000151 | 0.015 |
| 164 | Vit | -9.70 | -0.65 | 0.000631 | 0.025 |
| 165 | Rasgrp2 | -6.32 | -0.65 | 0.003202 | 0.050 |
| 166 | Arf2 | -10.85 | -0.65 | 0.000409 | 0.021 |
| 167 | Ttl | -6.81 | -0.65 | 0.002429 | 0.044 |
| 168 | Aldh1b1 | -6.79 | -0.65 | 0.002457 | 0.044 |
| 169 | Aldh1l2 | -9.22 | -0.65 | 0.000768 | 0.026 |
| 170 | Pou3f1 | -10.18 | -0.65 | 0.000525 | 0.023 |
| 171 | Pkia | -24.78 | -0.65 | 1.57E-05 | 0.008 |
| 172 | Phc1 | -15.07 | -0.64 | 0.000113 | 0.014 |
| 173 | Cdca5 | -8.88 | -0.64 | 0.000888 | 0.028 |
| 174 | Samd10 | -15.05 | -0.64 | 0.000114 | 0.014 |
| 175 | Dio3 | -6.78 | -0.64 | 0.002471 | 0.044 |
| 176 | Nrap | -25.38 | -0.64 | 1.43E-05 | 0.008 |
| 177 | Ebi3 | -20.89 | -0.64 | 3.1E-05 | 0.009 |
| 178 | Wdr48 | -9.94 | -0.64 | 0.000575 | 0.024 |
| 179 | BC035295 | -16.80 | -0.64 | 7.35E-05 | 0.013 |
| 180 | Mfsd9 | -12.77 | -0.64 | 0.000216 | 0.017 |
| 181 | Gcat | -9.40 | -0.64 | 0.000713 | 0.026 |
| 182 | Rpl29 | -9.69 | -0.64 | 0.000635 | 0.025 |
| 183 | BC025575 | -6.34 | -0.64 | 0.003176 | 0.049 |
| 184 | Htr1d | -10.49 | -0.63 | 0.000466 | 0.022 |
| 185 | Phtf2 | -15.73 | -0.63 | 9.55E-05 | 0.013 |
| 186 | Tekt1 | -11.84 | -0.63 | 0.000292 | 0.018 |
| 187 | Mvd | -12.00 | -0.63 | 0.000276 | 0.018 |
| 188 | 1700010A17Rik | -8.46 | -0.63 | 0.00107 | 0.030 |
| 189 | Erc2 | -12.74 | -0.63 | 0.000219 | 0.017 |
| 190 | AW551984 | -10.42 | -0.63 | 0.000479 | 0.022 |
| 191 | Lsm10 | -40.62 | -0.63 | 2.2E-06 | 0.003 |
| 192 | Pard6g | -9.33 | -0.63 | 0.000734 | 0.026 |
| 193 | 39516 | -8.97 | -0.62 | 0.000855 | 0.027 |
| 194 | Meig1 | -8.99 | -0.62 | 0.000848 | 0.027 |
| 195 | AI836003 | -7.98 | -0.62 | 0.001334 | 0.033 |
| 196 | Tekt2 | -6.32 | -0.62 | 0.003198 | 0.050 |
| 197 | Nasp | -14.18 | -0.62 | 0.000144 | 0.015 |
| 198 | Klhl23 | -7.81 | -0.62 | 0.00145 | 0.035 |
| 199 | Fxn | -15.48 | -0.61 | 0.000102 | 0.013 |
| 200 | Ablim3 | -6.47 | -0.61 | 0.002944 | 0.048 |
| 201 | Klf5 | -7.92 | -0.61 | 0.001372 | 0.034 |
| 202 | 1700012H17Rik | -14.03 | -0.61 | 0.00015 | 0.015 |
| 203 | Cdc42ep2 | -6.66 | -0.61 | 0.002644 | 0.045 |
| 204 | Emp1 | -7.16 | -0.60 | 0.002015 | 0.039 |
| 205 | Prkra | -15.30 | -0.60 | 0.000106 | 0.014 |
| 206 | Anapc5 | -13.49 | -0.60 | 0.000175 | 0.016 |
| 207 | Ppm1g | -19.91 | -0.60 | 3.75E-05 | 0.010 |
| 208 | 2410066E13Rik | -13.01 | -0.60 | 0.000202 | 0.017 |
| 209 | Egfl9 | -6.77 | -0.60 | 0.002483 | 0.044 |
| 210 | Nup43 | -8.52 | -0.59 | 0.001039 | 0.030 |
| 211 | Odz1 | -8.25 | -0.59 | 0.001178 | 0.031 |
| 212 | Gfod2 | -7.45 | -0.59 | 0.001733 | 0.037 |
| 213 | Rad9b | -9.68 | -0.59 | 0.000637 | 0.025 |
| 214 | 2310039H08Rik | -7.45 | -0.59 | 0.001733 | 0.037 |
| 215 | Taf6l | -10.37 | -0.59 | 0.000488 | 0.022 |
| 216 | 2310007H09Rik | -7.11 | -0.59 | 0.002071 | 0.040 |
| 217 | D11Ertd497e | -6.87 | -0.58 | 0.00235 | 0.043 |
| 218 | Csnk1e | -16.88 | -0.58 | 7.22E-05 | 0.013 |
| 219 | Fgd5 | -6.32 | -0.58 | 0.003206 | 0.050 |
| 220 | Hnrpab | -9.91 | -0.58 | 0.000583 | 0.024 |
| 221 | Telo2 | -9.28 | -0.58 | 0.000749 | 0.026 |
| 222 | 1700001L19Rik | -7.70 | -0.58 | 0.001533 | 0.035 |
| 223 | Myo1b | -12.96 | -0.58 | 0.000205 | 0.017 |
| 224 | Dnahc8 | -6.35 | -0.58 | 0.003144 | 0.049 |
| 225 | Rbm9 | -12.54 | -0.58 | 0.000233 | 0.017 |
| 226 | Rpp25 | -6.96 | -0.57 | 0.002242 | 0.042 |
| 227 | Ccdc101 | -12.51 | -0.57 | 0.000235 | 0.017 |
| 228 | Zfp184 | -7.97 | -0.57 | 0.001342 | 0.033 |
| 229 | Wnt7a | -6.90 | -0.57 | 0.002308 | 0.042 |
| 230 | Zdhhc12 | -9.27 | -0.57 | 0.000753 | 0.026 |
| 231 | Lrrc3 | -8.26 | -0.57 | 0.001172 | 0.031 |
| 232 | Hic2 | -7.26 | -0.57 | 0.001916 | 0.039 |
| 233 | Zfp358 | -7.87 | -0.57 | 0.00141 | 0.034 |
| 234 | Epb4.1l4a | -12.16 | -0.56 | 0.000263 | 0.018 |
| 235 | Stx1a | -7.33 | -0.56 | 0.001841 | 0.038 |
| 236 | 1700027N10Rik | -8.94 | -0.56 | 0.000867 | 0.027 |
| 237 | Lhx2 | -12.43 | -0.56 | 0.000241 | 0.017 |
| 238 | Prr15 | -7.43 | -0.56 | 0.001755 | 0.037 |
| 239 | Zscan21 | -6.81 | -0.55 | 0.002435 | 0.044 |
| 240 | Gpx7 | -9.17 | -0.55 | 0.000785 | 0.026 |
| 241 | Tbr1 | -6.30 | -0.55 | 0.003237 | 0.050 |
| 242 | Lzic | -9.33 | -0.55 | 0.000735 | 0.026 |
| 243 | Nln | -8.64 | -0.55 | 0.000985 | 0.029 |
| 244 | Tbcd | -9.40 | -0.55 | 0.000714 | 0.026 |
| 245 | 4930427A07Rik | -7.30 | -0.55 | 0.001868 | 0.039 |
| 246 | Slc6a4 | -8.27 | -0.55 | 0.001168 | 0.031 |
| 247 | 1190005I06Rik | -8.13 | -0.55 | 0.001245 | 0.032 |
| 248 | Snx8 | -16.57 | -0.55 | 7.76E-05 | 0.013 |
| 249 | Unc45a | -17.08 | -0.54 | 6.9E-05 | 0.012 |
| 250 | Ikzf4 | -10.00 | -0.54 | 0.000562 | 0.023 |
| 251 | Raf1 | -12.37 | -0.54 | 0.000245 | 0.017 |
| 252 | Stxbp2 | -7.01 | -0.54 | 0.002178 | 0.041 |
| 253 | Pip5k2b | -6.51 | -0.54 | 0.002879 | 0.047 |
| 254 | A930005I04Rik | -6.59 | -0.54 | 0.002745 | 0.046 |
| 255 | 3110056O03Rik | -7.82 | -0.54 | 0.001444 | 0.035 |
| 256 | 2010001J22Rik | -60.77 | -0.54 | 4.39E-07 | 0.002 |
| 257 | BC039093 | -14.17 | -0.54 | 0.000144 | 0.015 |
| 258 | D5Ertd593e | -8.75 | -0.54 | 0.000939 | 0.028 |
| 259 | 2210412D01Rik | -6.79 | -0.53 | 0.002456 | 0.044 |
| 260 | U46068 | -6.94 | -0.53 | 0.002265 | 0.042 |
| 261 | Mapre1 | -17.24 | -0.53 | 6.64E-05 | 0.012 |
| 262 | Isl1 | -7.23 | -0.53 | 0.001945 | 0.039 |
| 263 | Grb2 | -7.58 | -0.53 | 0.001623 | 0.036 |
| 264 | Zfp704 | -7.64 | -0.53 | 0.001578 | 0.036 |
| 265 | Rps19bp1 | -7.41 | -0.52 | 0.001772 | 0.038 |
| 266 | Zfp13 | -7.39 | -0.52 | 0.001784 | 0.038 |
| 267 | Sfrp1 | -6.49 | -0.51 | 0.002911 | 0.047 |
| 268 | 1810015A11Rik | -9.21 | -0.51 | 0.000773 | 0.026 |
| 269 | 6330442E10Rik | -8.22 | -0.51 | 0.001196 | 0.031 |
| 270 | Zfp46 | -9.52 | -0.51 | 0.000681 | 0.026 |
| 271 | Sh3d19 | -9.93 | -0.51 | 0.000577 | 0.024 |
| 272 | Fibcd1 | -8.50 | -0.51 | 0.00105 | 0.030 |
| 273 | Scml4 | -9.55 | -0.50 | 0.000671 | 0.025 |
| 274 | Edg4 | -12.11 | -0.50 | 0.000267 | 0.018 |
| 275 | Tjp3 | -8.96 | -0.50 | 0.000858 | 0.027 |
| 276 | Dynlrb2 | -10.49 | -0.49 | 0.000466 | 0.022 |
| 277 | Nipsnap3a | -9.03 | -0.49 | 0.000834 | 0.027 |
| 278 | Nmral1 | -9.92 | -0.49 | 0.00058 | 0.024 |
| 279 | Acaa1b | -8.26 | -0.48 | 0.001175 | 0.031 |
| 280 | Phf13 | -8.98 | -0.48 | 0.000851 | 0.027 |
| 281 | Shf | -6.49 | -0.48 | 0.002898 | 0.047 |
| 282 | 5830434P21Rik | -14.67 | -0.48 | 0.000126 | 0.014 |
| 283 | 2410137M14Rik | -14.08 | -0.48 | 0.000148 | 0.015 |
| 284 | Gap43 | -7.72 | -0.48 | 0.001512 | 0.035 |
| 285 | Kcnu1 | -9.42 | -0.48 | 0.000707 | 0.026 |
| 286 | Cdc42ep3 | -6.89 | -0.48 | 0.002326 | 0.042 |
| 287 | Bcorl1 | -10.41 | -0.48 | 0.00048 | 0.022 |
| 288 | Tcf19 | -7.88 | -0.48 | 0.001405 | 0.034 |
| 289 | 0610009J22Rik | -9.06 | -0.47 | 0.000824 | 0.027 |
| 290 | Nkiras2 | -9.08 | -0.47 | 0.000816 | 0.027 |
| 291 | Unc13b | -12.90 | -0.47 | 0.000208 | 0.017 |
| 292 | Rhebl1 | -7.44 | -0.47 | 0.001744 | 0.037 |
| 293 | 2810417H13Rik | -7.92 | -0.47 | 0.001374 | 0.034 |
| 294 | Ccdc71 | -6.89 | -0.47 | 0.002327 | 0.042 |
| 295 | Zbtb20 | -6.56 | -0.47 | 0.002794 | 0.047 |
| 296 | Ddx54 | -9.66 | -0.47 | 0.000643 | 0.025 |
| 297 | Exosc4 | -16.98 | -0.47 | 7.05E-05 | 0.012 |
| 298 | Nup205 | -25.70 | -0.47 | 1.36E-05 | 0.008 |
| 299 | Med4 | -9.57 | -0.47 | 0.000666 | 0.025 |
| 300 | Nsdhl | -6.53 | -0.47 | 0.002841 | 0.047 |
| 301 | Scarb1 | -8.67 | -0.47 | 0.000972 | 0.029 |
| 302 | Pqlc1 | -7.64 | -0.46 | 0.001576 | 0.036 |
| 303 | Mfge8 | -12.25 | -0.46 | 0.000255 | 0.017 |
| 304 | BC057371 | -7.43 | -0.46 | 0.001756 | 0.037 |
| 305 | Hcn3 | -7.14 | -0.46 | 0.00204 | 0.040 |
| 306 | Trp53rk | -8.62 | -0.46 | 0.000995 | 0.029 |
| 307 | 0610009O03Rik | -9.30 | -0.46 | 0.000744 | 0.026 |
| 308 | 1190002A17Rik | -6.97 | -0.46 | 0.002222 | 0.042 |
| 309 | Cfl1 | -23.27 | -0.46 | 2.02E-05 | 0.008 |
| 310 | B130050I23Rik | -6.95 | -0.46 | 0.002249 | 0.042 |
| 311 | Nradd | -7.97 | -0.45 | 0.001343 | 0.033 |
| 312 | Comtd1 | -8.27 | -0.45 | 0.001166 | 0.031 |
| 313 | Tmem132a | -8.60 | -0.45 | 0.001006 | 0.030 |
| 314 | Nipbl | -12.57 | -0.45 | 0.00023 | 0.017 |
| 315 | Kcnrg | -9.55 | -0.45 | 0.000673 | 0.026 |
| 316 | Rbx1 | -6.31 | -0.45 | 0.003232 | 0.050 |
| 317 | Suv420h2 | -10.98 | -0.45 | 0.00039 | 0.021 |
| 318 | Pfn3 | -6.34 | -0.45 | 0.003174 | 0.049 |
| 319 | Zfp324 | -7.39 | -0.45 | 0.001786 | 0.038 |
| 320 | BC011248 | -10.52 | -0.45 | 0.000463 | 0.022 |
| 321 | Car11 | -7.54 | -0.45 | 0.001661 | 0.037 |
| 322 | Polr3b | -9.52 | -0.44 | 0.00068 | 0.026 |
| 323 | 9430038I01Rik | -7.67 | -0.44 | 0.001556 | 0.036 |
| 324 | Polr2i | -6.37 | -0.44 | 0.003111 | 0.049 |
| 325 | Med12 | -7.26 | -0.44 | 0.001909 | 0.039 |
| 326 | 6430510M02Rik | -6.49 | -0.44 | 0.002902 | 0.047 |
| 327 | Sec61a1 | -6.73 | -0.44 | 0.002533 | 0.044 |
| 328 | Wfdc2 | -6.97 | -0.44 | 0.002231 | 0.042 |
| 329 | Zbtb22 | -14.93 | -0.44 | 0.000117 | 0.014 |
| 330 | Tmem39b | -7.93 | -0.44 | 0.001372 | 0.034 |
| 331 | Dmkn | -11.21 | -0.44 | 0.00036 | 0.020 |
| 332 | Dnmt3b | -8.74 | -0.44 | 0.000945 | 0.029 |
| 333 | Wdr31 | -8.53 | -0.44 | 0.001038 | 0.030 |
| 334 | Commd9 | -8.70 | -0.43 | 0.000962 | 0.029 |
| 335 | 5730590G19Rik | -27.17 | -0.43 | 1.09E-05 | 0.007 |
| 336 | Csdc2 | -7.28 | -0.43 | 0.001892 | 0.039 |
| 337 | Nup210 | -10.53 | -0.43 | 0.00046 | 0.022 |
| 338 | Tkt | -10.45 | -0.43 | 0.000475 | 0.022 |
| 339 | Rassf7 | -7.19 | -0.43 | 0.001985 | 0.039 |
| 340 | 4930455F23Rik | -6.78 | -0.43 | 0.002472 | 0.044 |
| 341 | Rusc2 | -8.62 | -0.43 | 0.000997 | 0.029 |
| 342 | Foxn4 | -6.96 | -0.43 | 0.002234 | 0.042 |
| 343 | Wtip | -8.99 | -0.43 | 0.000847 | 0.027 |
| 344 | Rufy3 | -8.22 | -0.43 | 0.001192 | 0.031 |
| 345 | Cdk5r1 | -8.54 | -0.43 | 0.001031 | 0.030 |
| 346 | Pou2f1 | -8.04 | -0.42 | 0.001301 | 0.033 |
| 347 | Il19 | -12.24 | -0.42 | 0.000255 | 0.017 |
| 348 | Cib2 | -6.52 | -0.42 | 0.002861 | 0.047 |
| 349 | Caln1 | -8.77 | -0.42 | 0.000931 | 0.028 |
| 350 | 2310008H04Rik | -6.60 | -0.42 | 0.002726 | 0.046 |
| 351 | Tmem132c | -16.40 | -0.42 | 8.1E-05 | 0.013 |
| 352 | Foxj2 | -6.71 | -0.42 | 0.002566 | 0.045 |
| 353 | 9630019K15Rik | -8.58 | -0.41 | 0.001012 | 0.030 |
| 354 | Spink2 | -6.38 | -0.41 | 0.003088 | 0.049 |
| 355 | Ppp4r1 | -6.43 | -0.41 | 0.003013 | 0.048 |
| 356 | Foxp2 | -15.57 | -0.41 | 9.93E-05 | 0.013 |
| 357 | E2f1 | -8.60 | -0.41 | 0.001004 | 0.030 |
| 358 | Lrrc8d | -7.66 | -0.41 | 0.001563 | 0.036 |
| 359 | Sfxn1 | -13.36 | -0.41 | 0.000182 | 0.016 |
| 360 | Shd | -7.71 | -0.41 | 0.00152 | 0.035 |
| 361 | Supt3h | -8.41 | -0.41 | 0.001094 | 0.030 |
| 362 | Slc39a11 | -8.11 | -0.41 | 0.001256 | 0.032 |
| 363 | Aldh3a1 | -6.36 | -0.41 | 0.003137 | 0.049 |
| 364 | Zbtb39 | -6.94 | -0.41 | 0.002266 | 0.042 |
| 365 | Ube3c | -6.55 | -0.41 | 0.002803 | 0.047 |
| 366 | Parp6 | -10.80 | -0.41 | 0.000417 | 0.021 |
| 367 | Crym | -7.34 | -0.40 | 0.001837 | 0.038 |
| 368 | D15Wsu169e | -10.89 | -0.40 | 0.000403 | 0.021 |
| 369 | Jmjd6 | -9.42 | -0.40 | 0.000708 | 0.026 |
| 370 | Cpne5 | -10.16 | -0.40 | 0.000528 | 0.023 |
| 371 | Csf2ra | -8.53 | -0.40 | 0.001035 | 0.030 |
| 372 | Taf1c | -10.03 | -0.40 | 0.000557 | 0.023 |
| 373 | Gdpd1 | -13.25 | -0.40 | 0.000187 | 0.016 |
| 374 | Il17rd | -6.59 | -0.40 | 0.00274 | 0.046 |
| 375 | Limk2 | -7.89 | -0.39 | 0.001395 | 0.034 |
| 376 | Rab6b | -7.26 | -0.39 | 0.001907 | 0.039 |
| 377 | Ube4b | -11.94 | -0.39 | 0.000282 | 0.018 |
| 378 | Nol9 | -10.25 | -0.39 | 0.000511 | 0.023 |
| 379 | Elof1 | -10.45 | -0.39 | 0.000475 | 0.022 |
| 380 | Pax9 | -9.28 | -0.39 | 0.00075 | 0.026 |
| 381 | Stau1 | -8.50 | -0.39 | 0.001052 | 0.030 |
| 382 | Gars | -24.66 | -0.38 | 1.61E-05 | 0.008 |
| 383 | Rnf152 | -6.72 | -0.38 | 0.002557 | 0.045 |
| 384 | Gpnmb | -7.23 | -0.38 | 0.001939 | 0.039 |
| 385 | Iqce | -8.96 | -0.38 | 0.000859 | 0.027 |
| 386 | Prlpl | -12.92 | -0.38 | 0.000207 | 0.017 |
| 387 | Slc4a7 | -6.59 | -0.38 | 0.002749 | 0.046 |
| 388 | Vps37b | -7.60 | -0.38 | 0.001606 | 0.036 |
| 389 | Rftn1 | -10.42 | -0.37 | 0.000479 | 0.022 |
| 390 | Letmd1 | -8.37 | -0.37 | 0.001114 | 0.030 |
| 391 | Dcps | -11.64 | -0.37 | 0.000311 | 0.019 |
| 392 | Tcp11 | -8.59 | -0.37 | 0.001008 | 0.030 |
| 393 | Orc6l | -7.83 | -0.37 | 0.001437 | 0.034 |
| 394 | Atpbd1b | -7.11 | -0.37 | 0.002067 | 0.040 |
| 395 | Gramd1a | -6.68 | -0.36 | 0.002616 | 0.045 |
| 396 | Cops7b | -7.75 | -0.36 | 0.00149 | 0.035 |
| 397 | Olfr736 | -11.88 | -0.36 | 0.000288 | 0.018 |
| 398 | Eftud2 | -10.48 | -0.36 | 0.000469 | 0.022 |
| 399 | Prr6 | -12.41 | -0.36 | 0.000243 | 0.017 |
| 400 | Phactr4 | -6.36 | -0.36 | 0.003141 | 0.049 |
| 401 | 2310036O22Rik | -8.47 | -0.35 | 0.001067 | 0.030 |
| 402 | Atpif1 | -6.47 | -0.35 | 0.00294 | 0.048 |
| 403 | Gpr120 | -7.80 | -0.35 | 0.001455 | 0.035 |
| 404 | Cxcr4 | -8.52 | -0.35 | 0.001039 | 0.030 |
| 405 | Morc2a | -9.38 | -0.35 | 0.00072 | 0.026 |
| 406 | Stat4 | -7.77 | -0.35 | 0.001478 | 0.035 |
| 407 | BC066135 | -7.53 | -0.35 | 0.001663 | 0.037 |
| 408 | Pcdh10 | -6.35 | -0.35 | 0.003157 | 0.049 |
| 409 | Prkag2 | -6.93 | -0.35 | 0.00228 | 0.042 |
| 410 | Cd82 | -8.48 | -0.35 | 0.001058 | 0.030 |
| 411 | Trim8 | -6.65 | -0.34 | 0.00266 | 0.045 |
| 412 | Nsmce2 | -6.91 | -0.34 | 0.002306 | 0.042 |
| 413 | Zfp3 | -7.22 | -0.34 | 0.001949 | 0.039 |
| 414 | Tsta3 | -9.26 | -0.34 | 0.000757 | 0.026 |
| 415 | Ccdc95 | -7.26 | -0.34 | 0.001913 | 0.039 |
| 416 | Gart | -8.94 | -0.34 | 0.000866 | 0.027 |
| 417 | Cma1 | -12.58 | -0.34 | 0.00023 | 0.017 |
| 418 | 2310016C16Rik | -6.49 | -0.34 | 0.002901 | 0.047 |
| 419 | Hoxd1 | -9.24 | -0.34 | 0.000762 | 0.026 |
| 420 | Itpk1 | -9.53 | -0.34 | 0.000678 | 0.026 |
| 421 | Tspan4 | -6.45 | -0.33 | 0.002971 | 0.048 |
| 422 | Timm50 | -7.17 | -0.33 | 0.002001 | 0.039 |
| 423 | Plcd1 | -7.07 | -0.33 | 0.002116 | 0.041 |
| 424 | Nup107 | -11.39 | -0.33 | 0.000339 | 0.019 |
| 425 | Ptprt | -7.21 | -0.33 | 0.001959 | 0.039 |
| 426 | Ccdc108 | -7.48 | -0.32 | 0.00171 | 0.037 |
| 427 | Csnk2a2 | -9.38 | -0.32 | 0.00072 | 0.026 |
| 428 | Pgd | -9.74 | -0.32 | 0.000623 | 0.025 |
| 429 | Incenp | -8.47 | -0.32 | 0.001066 | 0.030 |
| 430 | Hebp2 | -6.92 | -0.32 | 0.002285 | 0.042 |
| 431 | Ercc1 | -12.65 | -0.32 | 0.000225 | 0.017 |
| 432 | Kif27 | -7.98 | -0.32 | 0.00134 | 0.033 |
| 433 | Ccrk | -8.11 | -0.32 | 0.001255 | 0.032 |
| 434 | Zswim3 | -8.48 | -0.32 | 0.001061 | 0.030 |
| 435 | Ptprf | -6.82 | -0.32 | 0.00242 | 0.044 |
| 436 | Rhbdf1 | -6.47 | -0.31 | 0.002938 | 0.048 |
| 437 | Parp2 | -7.60 | -0.31 | 0.00161 | 0.036 |
| 438 | Faah | -6.34 | -0.31 | 0.003176 | 0.049 |
| 439 | Aof2 | -7.85 | -0.31 | 0.00142 | 0.034 |
| 440 | Supt16h | -7.89 | -0.31 | 0.001397 | 0.034 |
| 441 | Pak7 | -10.03 | -0.31 | 0.000557 | 0.023 |
| 442 | Rab8b | -9.09 | -0.31 | 0.000812 | 0.027 |
| 443 | Adra2c | -7.26 | -0.31 | 0.001914 | 0.039 |
| 444 | Cdk2 | -10.33 | -0.30 | 0.000496 | 0.022 |
| 445 | Epc1 | -7.52 | -0.30 | 0.001678 | 0.037 |
| 446 | 4632433K11Rik | -7.49 | -0.30 | 0.001696 | 0.037 |
| 447 | Lemd2 | -11.17 | -0.30 | 0.000365 | 0.020 |
