# Supplementary material for: Neonatal Maternal Deprivation Response and Developmental Changes in Gene Expression Revealed by Hypothalamic Gene Expression Profiling in Mice
Source: PLoS One. 2010 Feb 24;5(2):e9402. doi: 10.1371/journal.pone.0009402 (PMC2827556; doi:10.1371/journal.pone.0009402)
Supplement: Table S4 — Comparison 7. P5 Snord116del vs. P13 Snord116del (2-AD, 6-CD, 10-ED, 12-FD) vs. (14-GD, 16-GD). (0.46 MB DOC) [file pone.0009402.s005.doc]

Table S4. Comparison 7. P5 Snord116del vs. P13 Snord116del (2-AD, 6-CD, 10-ED, 12-FD)

vs. (14-GD, 16-GD). t-value is the ratio of the estimated change divided by the standard error.

BH-FDR: Bonferroni Hochberg false discovery rate.

| Comp7 | Gene Symbol | t-value | Fold_change | p-value | BH-FDR |
| --- | --- | --- | --- | --- | --- |
| 1 | Mobp | 25.58 | 5.52 | 1.39E-05 | 0.009 |
| 2 | Mag | 31.65 | 3.89 | 5.94E-06 | 0.008 |
| 3 | Mog | 27.11 | 3.77 | 1.10E-05 | 0.009 |
| 4 | Mbp | 22.31 | 3.18 | 2.39E-05 | 0.009 |
| 5 | Cldn11 | 36.90 | 2.99 | 3.22E-06 | 0.007 |
| 6 | Plp1 | 19.97 | 2.80 | 3.71E-05 | 0.010 |
| 7 | Ugt8a | 27.15 | 2.70 | 1.09E-05 | 0.009 |
| 8 | BC055107 | 13.48 | 2.61 | 0.000175 | 0.016 |
| 9 | 6330530A05Rik | 13.56 | 2.59 | 0.000171 | 0.016 |
| 10 | Fa2h | 19.69 | 2.56 | 3.92E-05 | 0.010 |
| 11 | Tmem10 | 20.23 | 2.52 | 3.52E-05 | 0.010 |
| 12 | Hapln4 | 11.79 | 2.16 | 0.000296 | 0.019 |
| 13 | 9530066K23Rik | 40.71 | 2.16 | 2.18E-06 | 0.007 |
| 14 | Selplg | 16.59 | 2.08 | 7.74E-05 | 0.012 |
| 15 | Enpp6 | 16.09 | 2.03 | 8.73E-05 | 0.013 |
| 16 | Tmem166 | 15.60 | 1.99 | 9.87E-05 | 0.013 |
| 17 | Oxt | 8.37 | 1.93 | 0.001114 | 0.035 |
| 18 | Ntsr2 | 19.63 | 1.88 | 3.97E-05 | 0.010 |
| 19 | Adamts4 | 16.85 | 1.82 | 7.27E-05 | 0.012 |
| 20 | Lgi3 | 24.63 | 1.80 | 1.61E-05 | 0.009 |
| 21 | Gja12 | 15.42 | 1.80 | 0.000103 | 0.014 |
| 22 | Hcrt | 9.14 | 1.67 | 0.000796 | 0.029 |
| 23 | 2900041A09Rik | 11.79 | 1.63 | 0.000296 | 0.019 |
| 24 | Gjb6 | 31.65 | 1.63 | 5.94E-06 | 0.008 |
| 25 | Klf9 | 17.03 | 1.57 | 6.98E-05 | 0.012 |
| 26 | Bcas1 | 16.87 | 1.57 | 7.23E-05 | 0.012 |
| 27 | Ogfod1 | 17.10 | 1.56 | 6.86E-05 | 0.012 |
| 28 | Gpd1 | 26.83 | 1.54 | 1.15E-05 | 0.009 |
| 29 | Cnp1 | 29.92 | 1.53 | 7.44E-06 | 0.008 |
| 30 | Hrh3 | 8.52 | 1.49 | 0.001042 | 0.034 |
| 31 | Cox6a2 | 14.73 | 1.48 | 0.000124 | 0.014 |
| 32 | Gprc5b | 13.26 | 1.48 | 0.000187 | 0.016 |
| 33 | Itih3 | 12.08 | 1.43 | 0.00027 | 0.019 |
| 34 | Mbnl2 | 11.02 | 1.40 | 0.000385 | 0.022 |
| 35 | Plxnb3 | 9.51 | 1.40 | 0.000683 | 0.028 |
| 36 | Dbp | 9.43 | 1.40 | 0.000704 | 0.028 |
| 37 | Sdc4 | 11.07 | 1.38 | 0.000379 | 0.022 |
| 38 | Cyp2d22 | 67.77 | 1.37 | 2.84E-07 | 0.005 |
| 39 | Mal | 25.36 | 1.36 | 1.44E-05 | 0.009 |
| 40 | Csrp1 | 15.65 | 1.34 | 9.74E-05 | 0.013 |
| 41 | Pllp | 9.96 | 1.31 | 0.00057 | 0.026 |
| 42 | Extl1 | 14.08 | 1.31 | 0.000148 | 0.016 |
| 43 | Htra1 | 22.82 | 1.29 | 2.18E-05 | 0.009 |
| 44 | Gpr37l1 | 8.72 | 1.29 | 0.000951 | 0.032 |
| 45 | Grin2c | 7.80 | 1.26 | 0.001456 | 0.039 |
| 46 | Glul | 9.28 | 1.23 | 0.000751 | 0.029 |
| 47 | 1700020C11Rik | 7.07 | 1.23 | 0.002117 | 0.047 |
| 48 | Bcl6 | 12.97 | 1.22 | 0.000204 | 0.017 |
| 49 | Gpr83 | 19.62 | 1.21 | 3.98E-05 | 0.010 |
| 50 | Aldoc | 7.39 | 1.16 | 0.001786 | 0.044 |
| 51 | Rasd1 | 8.98 | 1.16 | 0.00085 | 0.030 |
| 52 | Npb | 6.86 | 1.15 | 0.002359 | 0.049 |
| 53 | Sult1a1 | 21.31 | 1.14 | 2.86E-05 | 0.009 |
| 54 | Tspan2 | 8.38 | 1.14 | 0.001107 | 0.035 |
| 55 | Srpk3 | 12.08 | 1.14 | 0.000269 | 0.019 |
| 56 | Lims2 | 7.15 | 1.14 | 0.002025 | 0.046 |
| 57 | Slc17a7 | 12.49 | 1.14 | 0.000236 | 0.018 |
| 58 | Otub1 | 15.10 | 1.13 | 0.000112 | 0.014 |
| 59 | Cartpt | 8.19 | 1.13 | 0.00121 | 0.036 |
| 60 | Tuba4a | 20.72 | 1.13 | 3.21E-05 | 0.010 |
| 61 | Sparcl1 | 9.43 | 1.12 | 0.000706 | 0.028 |
| 62 | Cryab | 11.47 | 1.11 | 0.00033 | 0.020 |
| 63 | Emb | 10.65 | 1.11 | 0.00044 | 0.023 |
| 64 | Trh | 7.02 | 1.11 | 0.002165 | 0.047 |
| 65 | Pigz | 21.93 | 1.09 | 2.56E-05 | 0.009 |
| 66 | Kcnip4 | 10.76 | 1.09 | 0.000423 | 0.023 |
| 67 | Lynx1 | 8.95 | 1.09 | 0.000862 | 0.030 |
| 68 | Chi3l1 | 9.74 | 1.08 | 0.000622 | 0.027 |
| 69 | Zfp612 | 12.46 | 1.08 | 0.000239 | 0.018 |
| 70 | Kcna2 | 12.37 | 1.08 | 0.000246 | 0.018 |
| 71 | Slc44a1 | 22.80 | 1.07 | 2.19E-05 | 0.009 |
| 72 | Plekhh1 | 16.86 | 1.05 | 7.26E-05 | 0.012 |
| 73 | BC042720 | 6.99 | 1.04 | 0.002207 | 0.047 |
| 74 | Scn1b | 18.95 | 1.03 | 4.57E-05 | 0.010 |
| 75 | Bmp4 | 15.77 | 1.03 | 9.45E-05 | 0.013 |
| 76 | Gpr17 | 7.96 | 1.03 | 0.00135 | 0.038 |
| 77 | Neu4 | 17.93 | 1.03 | 5.68E-05 | 0.012 |
| 78 | Sncg | 8.82 | 1.02 | 0.000912 | 0.032 |
| 79 | Traf3 | 14.20 | 1.02 | 0.000143 | 0.015 |
| 80 | Atp2a1 | 9.50 | 1.01 | 0.000684 | 0.028 |
| 81 | Insl3 | 7.06 | 1.01 | 0.002119 | 0.047 |
| 82 | Eno2 | 18.22 | 1.01 | 5.34E-05 | 0.011 |
| 83 | Gng13 | 19.73 | 1.01 | 3.89E-05 | 0.010 |
| 84 | Tnnt1 | 12.50 | 1.00 | 0.000235 | 0.018 |
| 85 | Tanc1 | 8.85 | 1.00 | 0.000899 | 0.031 |
| 86 | Zmat3 | 9.92 | 0.99 | 0.00058 | 0.026 |
| 87 | Agt | 7.20 | 0.99 | 0.00197 | 0.045 |
| 88 | Susd2 | 7.23 | 0.99 | 0.001944 | 0.045 |
| 89 | Gltp | 9.31 | 0.98 | 0.00074 | 0.029 |
| 90 | Cyp2j9 | 12.27 | 0.98 | 0.000254 | 0.018 |
| 91 | Ankrd15 | 10.68 | 0.98 | 0.000435 | 0.023 |
| 92 | Ccdc28a | 8.49 | 0.97 | 0.001057 | 0.034 |
| 93 | 2410008K03Rik | 7.56 | 0.97 | 0.001641 | 0.042 |
| 94 | Cpne6 | 16.47 | 0.96 | 7.95E-05 | 0.013 |
| 95 | 2210011C24Rik | 13.63 | 0.96 | 0.000168 | 0.016 |
| 96 | Cox17 | 11.57 | 0.96 | 0.000318 | 0.020 |
| 97 | Flywch1 | 9.64 | 0.96 | 0.000646 | 0.027 |
| 98 | Asah2 | 10.05 | 0.96 | 0.000551 | 0.026 |
| 99 | Timp4 | 10.31 | 0.95 | 0.000499 | 0.024 |
| 100 | Kcnab2 | 23.45 | 0.94 | 1.96E-05 | 0.009 |
| 101 | Map2k1 | 16.59 | 0.94 | 7.73E-05 | 0.012 |
| 102 | Amotl1 | 13.90 | 0.94 | 0.000155 | 0.016 |
| 103 | Car4 | 6.96 | 0.93 | 0.002236 | 0.048 |
| 104 | Crebl2 | 9.14 | 0.92 | 0.000795 | 0.029 |
| 105 | Gstt3 | 8.53 | 0.92 | 0.001036 | 0.034 |
| 106 | Nckipsd | 14.47 | 0.91 | 0.000132 | 0.015 |
| 107 | 2010004A03Rik | 30.86 | 0.90 | 6.57E-06 | 0.008 |
| 108 | Pptc7 | 7.00 | 0.90 | 0.002192 | 0.047 |
| 109 | Pacsin1 | 15.93 | 0.90 | 9.07E-05 | 0.013 |
| 110 | Lysmd2 | 6.82 | 0.90 | 0.002418 | 0.050 |
| 111 | Hdc | 19.55 | 0.90 | 4.04E-05 | 0.010 |
| 112 | Dbndd2 | 33.57 | 0.89 | 4.70E-06 | 0.007 |
| 113 | 2310006M14Rik | 7.60 | 0.89 | 0.001607 | 0.042 |
| 114 | Tmem59l | 9.23 | 0.89 | 0.000766 | 0.029 |
| 115 | Hapln1 | 10.82 | 0.88 | 0.000415 | 0.023 |
| 116 | Gamt | 7.58 | 0.88 | 0.001626 | 0.042 |
| 117 | Sepp1 | 7.40 | 0.88 | 0.001779 | 0.044 |
| 118 | Xrcc3 | 7.44 | 0.87 | 0.001741 | 0.043 |
| 119 | Acot11 | 11.82 | 0.87 | 0.000293 | 0.019 |
| 120 | 6330406I15Rik | 15.25 | 0.87 | 0.000108 | 0.014 |
| 121 | Cplx1 | 9.55 | 0.86 | 0.000671 | 0.028 |
| 122 | Slc45a3 | 7.48 | 0.86 | 0.001704 | 0.043 |
| 123 | Cnksr3 | 10.90 | 0.85 | 0.000402 | 0.023 |
| 124 | Omg | 7.44 | 0.85 | 0.001744 | 0.043 |
| 125 | Kcnj16 | 9.54 | 0.84 | 0.000674 | 0.028 |
| 126 | Myoc | 12.94 | 0.84 | 0.000206 | 0.017 |
| 127 | F3 | 11.81 | 0.83 | 0.000295 | 0.019 |
| 128 | BC049806 | 12.34 | 0.83 | 0.000248 | 0.018 |
| 129 | Fgf1 | 24.45 | 0.82 | 1.66E-05 | 0.009 |
| 130 | Sox10 | 7.13 | 0.81 | 0.002044 | 0.046 |
| 131 | Itpr1 | 17.21 | 0.80 | 6.69E-05 | 0.012 |
| 132 | Igsf1 | 16.59 | 0.80 | 7.73E-05 | 0.012 |
| 133 | Dnaic1 | 20.20 | 0.80 | 3.54E-05 | 0.010 |
| 134 | Prom1 | 17.03 | 0.80 | 6.97E-05 | 0.012 |
| 135 | Abcg1 | 8.66 | 0.80 | 0.00098 | 0.033 |
| 136 | Abhd3 | 26.03 | 0.79 | 1.29E-05 | 0.009 |
| 137 | C230093N12Rik | 15.79 | 0.79 | 9.40E-05 | 0.013 |
| 138 | C330021A05Rik | 9.17 | 0.79 | 0.000785 | 0.029 |
| 139 | Sox8 | 37.21 | 0.79 | 3.12E-06 | 0.007 |
| 140 | Plxdc1 | 10.51 | 0.78 | 0.000464 | 0.024 |
| 141 | Cgrrf1 | 8.20 | 0.78 | 0.001207 | 0.036 |
| 142 | Elovl1 | 16.51 | 0.78 | 7.89E-05 | 0.013 |
| 143 | Peg3 | 12.83 | 0.78 | 0.000213 | 0.017 |
| 144 | 5730472N09Rik | 11.40 | 0.78 | 0.000338 | 0.021 |
| 145 | Sh3bgrl2 | 10.65 | 0.78 | 0.00044 | 0.023 |
| 146 | Edg8 | 34.63 | 0.78 | 4.15E-06 | 0.007 |
| 147 | 1100001E04Rik | 8.62 | 0.77 | 0.000996 | 0.033 |
| 148 | Fn3k | 8.12 | 0.77 | 0.001251 | 0.037 |
| 149 | Atp13a4 | 20.34 | 0.77 | 3.45E-05 | 0.010 |
| 150 | Tmem56 | 13.52 | 0.77 | 0.000173 | 0.016 |
| 151 | Epdr1 | 9.18 | 0.77 | 0.000781 | 0.029 |
| 152 | C030011O14Rik | 9.05 | 0.76 | 0.000826 | 0.030 |
| 153 | Ahcyl1 | 8.45 | 0.76 | 0.001073 | 0.034 |
| 154 | Elovl7 | 8.02 | 0.76 | 0.001312 | 0.037 |
| 155 | Acsbg1 | 9.95 | 0.75 | 0.000574 | 0.026 |
| 156 | Mrps12 | 7.00 | 0.75 | 0.002196 | 0.047 |
| 157 | Tsc22d4 | 7.30 | 0.75 | 0.001875 | 0.044 |
| 158 | Evi2a | 7.52 | 0.74 | 0.001672 | 0.042 |
| 159 | Slc25a33 | 10.55 | 0.74 | 0.000457 | 0.023 |
| 160 | Ache | 6.95 | 0.74 | 0.00225 | 0.048 |
| 161 | Nudt18 | 14.30 | 0.74 | 0.000139 | 0.015 |
| 162 | Scn1a | 12.21 | 0.73 | 0.000259 | 0.018 |
| 163 | Hrasls | 7.52 | 0.73 | 0.001677 | 0.042 |
| 164 | Cldn12 | 8.56 | 0.73 | 0.001024 | 0.034 |
| 165 | B230208H17Rik | 9.15 | 0.73 | 0.000793 | 0.029 |
| 166 | Pfkp | 12.57 | 0.73 | 0.00023 | 0.018 |
| 167 | Acsl6 | 8.24 | 0.73 | 0.001182 | 0.036 |
| 168 | Cx3cl1 | 8.28 | 0.73 | 0.001159 | 0.036 |
| 169 | Impact | 8.09 | 0.73 | 0.001266 | 0.037 |
| 170 | Mt1 | 11.79 | 0.72 | 0.000297 | 0.019 |
| 171 | Ar | 9.71 | 0.72 | 0.000629 | 0.027 |
| 172 | Etl4 | 7.14 | 0.71 | 0.002032 | 0.046 |
| 173 | Crybb1 | 13.65 | 0.70 | 0.000167 | 0.016 |
| 174 | Atp6v0a2 | 15.32 | 0.70 | 0.000106 | 0.014 |
| 175 | Rgs7bp | 17.16 | 0.70 | 6.77E-05 | 0.012 |
| 176 | Mt2 | 29.86 | 0.70 | 7.49E-06 | 0.008 |
| 177 | Rwdd2 | 9.45 | 0.70 | 0.000701 | 0.028 |
| 178 | Gpx3 | 7.47 | 0.70 | 0.001716 | 0.043 |
| 179 | Prodh | 13.42 | 0.70 | 0.000179 | 0.016 |
| 180 | A830036E02Rik | 8.05 | 0.69 | 0.001291 | 0.037 |
| 181 | Mink1 | 6.81 | 0.69 | 0.002424 | 0.050 |
| 182 | Lin7b | 8.89 | 0.68 | 0.000886 | 0.031 |
| 183 | Gaa | 8.72 | 0.68 | 0.000952 | 0.032 |
| 184 | Tmem80 | 29.21 | 0.68 | 8.18E-06 | 0.008 |
| 185 | Hpcal4 | 7.36 | 0.67 | 0.001814 | 0.044 |
| 186 | S100a13 | 7.76 | 0.67 | 0.001487 | 0.040 |
| 187 | Necap1 | 7.61 | 0.67 | 0.0016 | 0.042 |
| 188 | Dexi | 9.69 | 0.67 | 0.000636 | 0.027 |
| 189 | C1ql2 | 9.50 | 0.66 | 0.000684 | 0.028 |
| 190 | Itm2c | 9.47 | 0.66 | 0.000694 | 0.028 |
| 191 | A630054L15Rik | 7.49 | 0.66 | 0.001696 | 0.043 |
| 192 | Hspa5 | 13.99 | 0.66 | 0.000151 | 0.016 |
| 193 | Gal3st1 | 8.09 | 0.66 | 0.00127 | 0.037 |
| 194 | Brs3 | 7.22 | 0.65 | 0.001951 | 0.045 |
| 195 | 9130213B05Rik | 7.87 | 0.65 | 0.001411 | 0.039 |
| 196 | 1110012N22Rik | 11.02 | 0.65 | 0.000385 | 0.022 |
| 197 | Krt222 | 7.36 | 0.65 | 0.001812 | 0.044 |
| 198 | Axud1 | 8.25 | 0.65 | 0.001175 | 0.036 |
| 199 | Dgkg | 11.81 | 0.64 | 0.000295 | 0.019 |
| 200 | Ephx1 | 12.75 | 0.64 | 0.000218 | 0.017 |
| 201 | Dmrtc1a | 9.64 | 0.64 | 0.000647 | 0.027 |
| 202 | 9630046K23Rik | 15.07 | 0.64 | 0.000113 | 0.014 |
| 203 | Sgtb | 12.26 | 0.63 | 0.000254 | 0.018 |
| 204 | Phkg1 | 9.20 | 0.63 | 0.000774 | 0.029 |
| 205 | Chn2 | 12.52 | 0.63 | 0.000234 | 0.018 |
| 206 | Lrrc24 | 7.95 | 0.63 | 0.001355 | 0.038 |
| 207 | 1700037H04Rik | 12.87 | 0.63 | 0.00021 | 0.017 |
| 208 | Pstpip1 | 10.87 | 0.62 | 0.000407 | 0.023 |
| 209 | 1190007F08Rik | 20.57 | 0.62 | 3.30E-05 | 0.010 |
| 210 | C920006C10Rik | 8.84 | 0.62 | 0.000903 | 0.031 |
| 211 | Ttc7b | 8.41 | 0.62 | 0.001096 | 0.035 |
| 212 | Ppargc1b | 12.37 | 0.62 | 0.000245 | 0.018 |
| 213 | Idh3a | 7.83 | 0.62 | 0.001435 | 0.039 |
| 214 | Tmem38a | 6.89 | 0.61 | 0.002326 | 0.049 |
| 215 | Rasgrf1 | 12.32 | 0.61 | 0.00025 | 0.018 |
| 216 | Baiap2 | 7.95 | 0.61 | 0.001354 | 0.038 |
| 217 | Tiam1 | 9.81 | 0.61 | 0.000605 | 0.027 |
| 218 | Mfsd2 | 11.02 | 0.61 | 0.000385 | 0.022 |
| 219 | Cit | 9.34 | 0.60 | 0.000731 | 0.029 |
| 220 | Dtd1 | 7.99 | 0.60 | 0.001328 | 0.038 |
| 221 | BC054438 | 12.32 | 0.60 | 0.000249 | 0.018 |
| 222 | Grhpr | 7.28 | 0.60 | 0.00189 | 0.045 |
| 223 | Dio2 | 14.36 | 0.60 | 0.000137 | 0.015 |
| 224 | Ptplb | 10.45 | 0.60 | 0.000474 | 0.024 |
| 225 | Sult4a1 | 7.31 | 0.60 | 0.001866 | 0.044 |
| 226 | Ndg2 | 8.15 | 0.59 | 0.001233 | 0.036 |
| 227 | Mapk14 | 11.45 | 0.59 | 0.000332 | 0.020 |
| 228 | Mdh1 | 6.88 | 0.58 | 0.002344 | 0.049 |
| 229 | Nfic | 6.80 | 0.58 | 0.002439 | 0.050 |
| 230 | Avil | 13.36 | 0.58 | 0.000181 | 0.016 |
| 231 | Dnm1 | 8.21 | 0.58 | 0.0012 | 0.036 |
| 232 | Pop5 | 10.67 | 0.58 | 0.000437 | 0.023 |
| 233 | Cntnap1 | 14.87 | 0.58 | 0.000119 | 0.014 |
| 234 | Tmem33 | 7.55 | 0.58 | 0.001653 | 0.042 |
| 235 | Cd74 | 10.43 | 0.58 | 0.000477 | 0.024 |
| 236 | Slc25a14 | 9.98 | 0.57 | 0.000566 | 0.026 |
| 237 | Sbds | 13.06 | 0.57 | 0.000198 | 0.016 |
| 238 | Faim2 | 10.28 | 0.57 | 0.000504 | 0.024 |
| 239 | 2610204L23Rik | 7.21 | 0.57 | 0.001959 | 0.045 |
| 240 | BC006662 | 13.14 | 0.57 | 0.000194 | 0.016 |
| 241 | Nrip3 | 9.25 | 0.57 | 0.00076 | 0.029 |
| 242 | Ank | 9.37 | 0.57 | 0.000723 | 0.028 |
| 243 | Vamp4 | 7.76 | 0.56 | 0.001488 | 0.040 |
| 244 | Sirt3 | 7.77 | 0.56 | 0.00148 | 0.040 |
| 245 | Yipf2 | 9.40 | 0.56 | 0.000713 | 0.028 |
| 246 | Stip1 | 14.02 | 0.56 | 0.00015 | 0.016 |
| 247 | Gmpr | 11.40 | 0.56 | 0.000338 | 0.021 |
| 248 | Hdhd2 | 15.58 | 0.56 | 9.91E-05 | 0.013 |
| 249 | Sgms1 | 19.88 | 0.56 | 3.78E-05 | 0.010 |
| 250 | Car14 | 7.67 | 0.55 | 0.001555 | 0.041 |
| 251 | Gpc5 | 12.10 | 0.55 | 0.000268 | 0.019 |
| 252 | Timm17a | 10.03 | 0.55 | 0.000555 | 0.026 |
| 253 | Sv2a | 19.93 | 0.55 | 3.74E-05 | 0.010 |
| 254 | Bace2 | 13.50 | 0.55 | 0.000174 | 0.016 |
| 255 | Trp53inp2 | 9.40 | 0.54 | 0.000715 | 0.028 |
| 256 | 6430548M08Rik | 7.92 | 0.54 | 0.001374 | 0.038 |
| 257 | D430028G21Rik | 9.81 | 0.54 | 0.000605 | 0.027 |
| 258 | Galntl1 | 8.10 | 0.54 | 0.001262 | 0.037 |
| 259 | Emcn | 9.53 | 0.53 | 0.000678 | 0.028 |
| 260 | Clip4 | 11.39 | 0.53 | 0.000339 | 0.021 |
| 261 | Rras | 16.00 | 0.53 | 8.92E-05 | 0.013 |
| 262 | 4933406E20Rik | 10.97 | 0.53 | 0.000392 | 0.022 |
| 263 | Mrpl4 | 9.70 | 0.53 | 0.000633 | 0.027 |
| 264 | AU040829 | 10.73 | 0.53 | 0.000428 | 0.023 |
| 265 | Hsd3b7 | 7.20 | 0.53 | 0.001974 | 0.045 |
| 266 | Jam3 | 8.80 | 0.52 | 0.00092 | 0.032 |
| 267 | Scrg1 | 8.00 | 0.52 | 0.001323 | 0.038 |
| 268 | Paqr7 | 11.18 | 0.52 | 0.000364 | 0.022 |
| 269 | Ltbp3 | 10.04 | 0.51 | 0.000554 | 0.026 |
| 270 | Ptprn2 | 8.15 | 0.51 | 0.001231 | 0.036 |
| 271 | 1110049F12Rik | 11.79 | 0.51 | 0.000296 | 0.019 |
| 272 | Rab3gap1 | 7.99 | 0.51 | 0.00133 | 0.038 |
| 273 | Clu | 12.04 | 0.51 | 0.000273 | 0.019 |
| 274 | Ankrd56 | 17.29 | 0.50 | 6.57E-05 | 0.012 |
| 275 | Idh3b | 8.32 | 0.50 | 0.001138 | 0.035 |
| 276 | Atp6v1g2 | 11.55 | 0.50 | 0.000321 | 0.020 |
| 277 | Neu1 | 8.77 | 0.49 | 0.000933 | 0.032 |
| 278 | Ndufs2 | 7.36 | 0.49 | 0.00182 | 0.044 |
| 279 | Tmed4 | 26.82 | 0.49 | 1.15E-05 | 0.009 |
| 280 | Tsr2 | 10.23 | 0.49 | 0.000515 | 0.025 |
| 281 | Idh3g | 13.60 | 0.49 | 0.000169 | 0.016 |
| 282 | Chrm2 | 9.34 | 0.49 | 0.000732 | 0.029 |
| 283 | Atp6v1d | 7.01 | 0.48 | 0.002178 | 0.047 |
| 284 | Kif21a | 7.88 | 0.48 | 0.001404 | 0.039 |
| 285 | Zdhhc24 | 13.13 | 0.48 | 0.000194 | 0.016 |
| 286 | Gpr108 | 7.46 | 0.48 | 0.001726 | 0.043 |
| 287 | Crhbp | 9.16 | 0.48 | 0.000787 | 0.029 |
| 288 | Plcxd1 | 7.08 | 0.48 | 0.002101 | 0.047 |
| 289 | Adc | 12.03 | 0.48 | 0.000274 | 0.019 |
| 290 | BC031353 | 8.86 | 0.48 | 0.000897 | 0.031 |
| 291 | Rnf122 | 10.30 | 0.48 | 0.000501 | 0.024 |
| 292 | 2310061C15Rik | 12.72 | 0.47 | 0.00022 | 0.017 |
| 293 | Socs5 | 15.44 | 0.47 | 0.000103 | 0.014 |
| 294 | Atp1b1 | 7.12 | 0.47 | 0.002057 | 0.046 |
| 295 | Abhd4 | 10.85 | 0.46 | 0.00041 | 0.023 |
| 296 | Atp6v1c1 | 6.90 | 0.46 | 0.002308 | 0.049 |
| 297 | Mrvi1 | 7.23 | 0.45 | 0.001944 | 0.045 |
| 298 | Tmem180 | 8.98 | 0.45 | 0.000851 | 0.030 |
| 299 | Ngb | 6.89 | 0.45 | 0.002327 | 0.049 |
| 300 | Inppl1 | 8.31 | 0.45 | 0.001147 | 0.035 |
| 301 | Slc2a6 | 7.38 | 0.45 | 0.001793 | 0.044 |
| 302 | Cpt2 | 10.17 | 0.45 | 0.000526 | 0.025 |
| 303 | Ndufb10 | 6.91 | 0.45 | 0.002296 | 0.049 |
| 304 | Cml1 | 11.47 | 0.45 | 0.00033 | 0.020 |
| 305 | Slc35b4 | 6.79 | 0.45 | 0.002454 | 0.050 |
| 306 | Map4k2 | 19.87 | 0.44 | 3.78E-05 | 0.010 |
| 307 | Tef | 13.15 | 0.44 | 0.000193 | 0.016 |
| 308 | Usp11 | 11.14 | 0.44 | 0.00037 | 0.022 |
| 309 | Slc6a8 | 21.37 | 0.43 | 2.84E-05 | 0.009 |
| 310 | Plcd4 | 35.91 | 0.43 | 3.59E-06 | 0.007 |
| 311 | Eif2ak3 | 9.91 | 0.43 | 0.000583 | 0.026 |
| 312 | Maob | 9.52 | 0.43 | 0.00068 | 0.028 |
| 313 | Grina | 7.70 | 0.43 | 0.001529 | 0.040 |
| 314 | Urod | 8.68 | 0.43 | 0.00097 | 0.033 |
| 315 | Rims2 | 10.46 | 0.42 | 0.000473 | 0.024 |
| 316 | Opn1mw | 12.27 | 0.42 | 0.000254 | 0.018 |
| 317 | Actr1b | 8.96 | 0.41 | 0.000858 | 0.030 |
| 318 | Ndufa13 | 7.23 | 0.41 | 0.001944 | 0.045 |
| 319 | Mapk4 | 10.34 | 0.41 | 0.000493 | 0.024 |
| 320 | Pdxdc1 | 10.81 | 0.40 | 0.000415 | 0.023 |
| 321 | Mtac2d1 | 6.90 | 0.40 | 0.002308 | 0.049 |
| 322 | St8sia5 | 7.10 | 0.40 | 0.002083 | 0.046 |
| 323 | 4732418C07Rik | 8.89 | 0.40 | 0.000884 | 0.031 |
| 324 | Cdc37l1 | 7.78 | 0.40 | 0.001473 | 0.040 |
| 325 | Slc25a3 | 8.67 | 0.40 | 0.000975 | 0.033 |
| 326 | Ndufa2 | 6.80 | 0.40 | 0.002439 | 0.050 |
| 327 | Zcchc17 | 13.2 | 0.40 | 0.000195 | 0.016 |
| 328 | Fbxo7 | 9.75 | 0.40 | 0.000619 | 0.027 |
| 329 | Prelp | 7.13 | 0.40 | 0.002047 | 0.046 |
| 330 | Pltp | 7.31 | 0.39 | 0.001859 | 0.044 |
| 331 | Wdr45 | 13.5 | 0.39 | 0.000162 | 0.016 |
| 332 | Iars2 | 7.25 | 0.38 | 0.00192 | 0.045 |
| 333 | Syt12 | 10.90 | 0.38 | 0.000403 | 0.023 |
| 334 | Sdf4 | 7.26 | 0.37 | 0.001907 | 0.045 |
| 335 | Mcee | 9.07 | 0.37 | 0.000818 | 0.030 |
| 336 | Pank4 | 7.53 | 0.37 | 0.001664 | 0.042 |
| 337 | Impa2 | 8.43 | 0.37 | 0.001084 | 0.035 |
| 338 | Esd | 8.42 | 0.37 | 0.00109 | 0.035 |
| 339 | Pla2g7 | 9.56 | 0.37 | 0.000668 | 0.028 |
| 340 | Amhr2 | 9.59 | 0.36 | 0.000661 | 0.028 |
| 341 | Nkrf | 7.37 | 0.36 | 0.001809 | 0.044 |
| 342 | Itpkb | 10.09 | 0.36 | 0.000543 | 0.026 |
| 343 | 1110020P15Rik | 15.91 | 0.36 | 9.13E-05 | 0.013 |
| 344 | Fgf9 | 7.75 | 0.36 | 0.001494 | 0.040 |
| 345 | Aip | 14.33 | 0.36 | 0.000138 | 0.015 |
| 346 | Colec11 | 6.83 | 0.35 | 0.002405 | 0.050 |
| 347 | C030018G13Rik | 8.68 | 0.35 | 0.00097 | 0.033 |
| 348 | Psmc5 | 8.01 | 0.35 | 0.001317 | 0.038 |
| 349 | Lmln | 9.26 | 0.34 | 0.000757 | 0.029 |
| 350 | Mppe1 | 7.82 | 0.34 | 0.001444 | 0.039 |
| 351 | Mis12 | 8.16 | 0.34 | 0.001229 | 0.036 |
| 352 | D10Jhu81e | 9.33 | 0.34 | 0.000734 | 0.029 |
| 353 | Uqcrc1 | 8.73 | 0.33 | 0.00095 | 0.032 |
| 354 | Gria1 | 13.13 | 0.33 | 0.000194 | 0.016 |
| 355 | Tmem100 | 7.01 | 0.33 | 0.002177 | 0.047 |
| 356 | Cd9 | 8.93 | 0.32 | 0.000868 | 0.031 |
| 357 | Preb | 8.58 | 0.32 | 0.001014 | 0.033 |
| 358 | Ankrd40 | 10.66 | 0.32 | 0.000439 | 0.023 |
| 359 | Serpina9 | 8.18 | 0.31 | 0.001215 | 0.036 |
| 360 | Pld1 | 7.59 | 0.31 | 0.001615 | 0.042 |
| 361 | Palmd | 7.41 | 0.30 | 0.001774 | 0.044 |
| 362 | A430005L14Rik | 7.31 | 0.30 | 0.001864 | 0.044 |
| 363 | Tatdn3 | 8.16 | 0.30 | 0.001229 | 0.036 |
| 364 | Strap | 7.51 | 0.30 | 0.00168 | 0.042 |

| Comp7 | Gene Symbol | t-value | Fold change | p-value | BH-FDR |
| --- | --- | --- | --- | --- | --- |
| 1 | St8sia2 | -14.08 | -2.87 | 0.000148 | 0.016 |
| 2 | Dpysl3 | -16.89 | -1.83 | 7.21E-05 | 0.012 |
| 3 | Sbk1 | -7.82 | -1.72 | 0.001445 | 0.039 |
| 4 | Akr1c19 | -23.69 | -1.66 | 1.88E-05 | 0.009 |
| 5 | 2410146L05Rik | -49.92 | -1.52 | 9.64E-07 | 0.007 |
| 6 | Sh3bp2 | -17.18 | -1.47 | 6.74E-05 | 0.012 |
| 7 | Smpd3 | -13.14 | -1.46 | 0.000194 | 0.016 |
| 8 | Dpysl5 | -23.06 | -1.46 | 2.09E-05 | 0.009 |
| 9 | Pafah1b3 | -12.73 | -1.41 | 0.00022 | 0.017 |
| 10 | Vash2 | -9.20 | -1.35 | 0.000774 | 0.029 |
| 11 | Ypel1 | -11.30 | -1.35 | 0.00035 | 0.021 |
| 12 | B3gnt5 | -16.12 | -1.31 | 8.66E-05 | 0.013 |
| 13 | Rab3d | -24.32 | -1.29 | 1.70E-05 | 0.009 |
| 14 | Ccdc120 | -19.54 | -1.28 | 4.04E-05 | 0.010 |
| 15 | Rac3 | -15.03 | -1.28 | 0.000114 | 0.014 |
| 16 | Cdkn1a | -7.73 | -1.28 | 0.001508 | 0.040 |
| 17 | 4930519N16Rik | -9.32 | -1.27 | 0.000737 | 0.029 |
| 18 | Crmp1 | -13.79 | -1.26 | 0.00016 | 0.016 |
| 19 | Zbtb5 | -10.66 | -1.24 | 0.000438 | 0.023 |
| 20 | Gpc2 | -11.58 | -1.21 | 0.000317 | 0.020 |
| 21 | Bzw2 | -33.65 | -1.15 | 4.65E-06 | 0.007 |
| 22 | Socs2 | -9.30 | -1.15 | 0.000745 | 0.029 |
| 23 | Tead2 | -9.28 | -1.13 | 0.00075 | 0.029 |
| 24 | Dkkl1 | -16.28 | -1.11 | 8.34E-05 | 0.013 |
| 25 | Lefty2 | -13.78 | -1.11 | 0.000161 | 0.016 |
| 26 | Klf7 | -14.52 | -1.11 | 0.000131 | 0.015 |
| 27 | 9030425E11Rik | -12.36 | -1.10 | 0.000246 | 0.018 |
| 28 | Hn1 | -13.88 | -1.07 | 0.000156 | 0.016 |
| 29 | 1110038B12Rik | -14.27 | -1.05 | 0.00014 | 0.015 |
| 30 | C1qtnf2 | -7.05 | -1.04 | 0.002136 | 0.047 |
| 31 | Nt5dc2 | -16.99 | -1.00 | 7.04E-05 | 0.012 |
| 32 | 2610110G12Rik | -7.55 | -1.00 | 0.001647 | 0.042 |
| 33 | Stag3 | -10.61 | -1.00 | 0.000446 | 0.023 |
| 34 | Tubb2b | -16.35 | -0.98 | 8.19E-05 | 0.013 |
| 35 | Nans | -9.49 | -0.98 | 0.000687 | 0.028 |
| 36 | Adh7 | -7.21 | -0.97 | 0.00196 | 0.045 |
| 37 | D330027H18Rik | -10.77 | -0.97 | 0.000422 | 0.023 |
| 38 | Lrrc23 | -13.86 | -0.94 | 0.000157 | 0.016 |
| 39 | Gpr21 | -8.05 | -0.94 | 0.001293 | 0.037 |
| 40 | Mcm5 | -13.87 | -0.93 | 0.000157 | 0.016 |
| 41 | Lck | -9.50 | -0.93 | 0.000684 | 0.028 |
| 42 | Igsf3 | -10.29 | -0.93 | 0.000503 | 0.024 |
| 43 | Aldh1b1 | -10.92 | -0.92 | 0.000399 | 0.023 |
| 44 | Arrdc4 | -7.38 | -0.92 | 0.001794 | 0.044 |
| 45 | Ccnu | -9.48 | -0.92 | 0.00069 | 0.028 |
| 46 | Ykt6 | -13.53 | -0.91 | 0.000173 | 0.016 |
| 47 | Zfp57 | -8.79 | -0.90 | 0.000923 | 0.032 |
| 48 | Dcx | -13.03 | -0.90 | 0.0002 | 0.017 |
| 49 | AW146242 | -12.36 | -0.89 | 0.000246 | 0.018 |
| 50 | 2810003C17Rik | -10.91 | -0.89 | 0.000401 | 0.023 |
| 51 | Lypd3 | -8.93 | -0.88 | 0.000868 | 0.031 |
| 52 | Lrfn4 | -14.01 | -0.87 | 0.000151 | 0.016 |
| 53 | Ibrdc3 | -6.87 | -0.86 | 0.002354 | 0.049 |
| 54 | Gipc2 | -15.67 | -0.85 | 9.69E-05 | 0.013 |
| 55 | Nuak2 | -22.46 | -0.85 | 2.33E-05 | 0.009 |
| 56 | AW551984 | -22.36 | -0.85 | 2.37E-05 | 0.009 |
| 57 | Ceecam1 | -22.23 | -0.85 | 2.42E-05 | 0.009 |
| 58 | Cdkn1c | -10.39 | -0.83 | 0.000484 | 0.024 |
| 59 | Aloxe3 | -15.63 | -0.83 | 9.79E-05 | 0.013 |
| 60 | Slc14a2 | -8.96 | -0.83 | 0.000859 | 0.030 |
| 61 | Smarce1 | -21.84 | -0.83 | 2.60E-05 | 0.009 |
| 62 | Drctnnb1a | -8.96 | -0.82 | 0.000857 | 0.030 |
| 63 | Dctd | -15.99 | -0.82 | 8.93E-05 | 0.013 |
| 64 | Zfp184 | -15.09 | -0.82 | 0.000112 | 0.014 |
| 65 | Sct | -8.26 | -0.82 | 0.001174 | 0.036 |
| 66 | D0H4S114 | -7.35 | -0.81 | 0.001828 | 0.044 |
| 67 | Gmip | -7.67 | -0.80 | 0.001556 | 0.041 |
| 68 | BC065085 | -8.63 | -0.80 | 0.000991 | 0.033 |
| 69 | Samd10 | -16.20 | -0.80 | 8.49E-05 | 0.013 |
| 70 | 2210412D01Rik | -13.61 | -0.80 | 0.000169 | 0.016 |
| 71 | Igf2bp3 | -8.78 | -0.80 | 0.000928 | 0.032 |
| 72 | Csrp2 | -8.25 | -0.79 | 0.001176 | 0.036 |
| 73 | 1700016K19Rik | -10.77 | -0.79 | 0.000422 | 0.023 |
| 74 | 2310007A19Rik | -9.98 | -0.79 | 0.000566 | 0.026 |
| 75 | 9630031F12Rik | -9.62 | -0.79 | 0.000653 | 0.028 |
| 76 | Psme2 | -6.85 | -0.79 | 0.002375 | 0.049 |
| 77 | Nme6 | -13.11 | -0.79 | 0.000196 | 0.016 |
| 78 | 2310057J16Rik | -9.66 | -0.79 | 0.000641 | 0.027 |
| 79 | Lgals3 | -7.83 | -0.78 | 0.001437 | 0.039 |
| 80 | Rpl22 | -10.84 | -0.78 | 0.000412 | 0.023 |
| 81 | Slc26a1 | -6.85 | -0.78 | 0.002375 | 0.049 |
| 82 | Mfap3 | -8.90 | -0.77 | 0.00088 | 0.031 |
| 83 | Zbtb8 | -10.94 | -0.77 | 0.000396 | 0.023 |
| 84 | Emid2 | -8.41 | -0.76 | 0.001094 | 0.035 |
| 85 | Gprin1 | -22.05 | -0.76 | 2.50E-05 | 0.009 |
| 86 | AI836003 | -9.56 | -0.76 | 0.000668 | 0.028 |
| 87 | Chrna3 | -17.26 | -0.75 | 6.61E-05 | 0.012 |
| 88 | 2810046M22Rik | -11.08 | -0.75 | 0.000378 | 0.022 |
| 89 | AI646023 | -8.39 | -0.75 | 0.001103 | 0.035 |
| 90 | Ccdc23 | -8.10 | -0.75 | 0.001264 | 0.037 |
| 91 | Ica1l | -8.19 | -0.74 | 0.001212 | 0.036 |
| 92 | Zfp41 | -10.80 | -0.74 | 0.000418 | 0.023 |
| 93 | C030003D03Rik | -12.79 | -0.74 | 0.000215 | 0.017 |
| 94 | Nkd2 | -7.30 | -0.73 | 0.00187 | 0.044 |
| 95 | Tceb1 | -6.84 | -0.73 | 0.002394 | 0.050 |
| 96 | Foxa1 | -9.65 | -0.73 | 0.000644 | 0.027 |
| 97 | Erc2 | -9.80 | -0.73 | 0.000608 | 0.027 |
| 98 | BC005764 | -9.13 | -0.72 | 0.000799 | 0.029 |
| 99 | Phtf2 | -10.67 | -0.72 | 0.000437 | 0.023 |
| 100 | BC068157 | -14.47 | -0.72 | 0.000133 | 0.015 |
| 101 | BC025575 | -7.73 | -0.72 | 0.001511 | 0.040 |
| 102 | Dlk1 | -10.67 | -0.72 | 0.000437 | 0.023 |
| 103 | 2010310D06Rik | -7.98 | -0.72 | 0.001336 | 0.038 |
| 104 | Elavl3 | -10.81 | -0.72 | 0.000415 | 0.023 |
| 105 | Klk8 | -9.66 | -0.71 | 0.000642 | 0.027 |
| 106 | Pih1d1 | -9.20 | -0.70 | 0.000775 | 0.029 |
| 107 | 1500015O10Rik | -8.25 | -0.70 | 0.001176 | 0.036 |
| 108 | 2410015N17Rik | -7.01 | -0.69 | 0.002186 | 0.047 |
| 109 | Vps37d | -11.82 | -0.69 | 0.000293 | 0.019 |
| 110 | Nup43 | -9.80 | -0.68 | 0.000607 | 0.027 |
| 111 | Ankfy1 | -8.23 | -0.68 | 0.001186 | 0.036 |
| 112 | 9-Mar | -11.82 | -0.68 | 0.000293 | 0.019 |
| 113 | Ubap2 | -17.54 | -0.68 | 6.21E-05 | 0.012 |
| 114 | Scml4 | -11.80 | -0.68 | 0.000295 | 0.019 |
| 115 | Nnat | -7.45 | -0.68 | 0.001735 | 0.043 |
| 116 | Tubb3 | -9.44 | -0.68 | 0.000702 | 0.028 |
| 117 | Crcp | -7.28 | -0.67 | 0.001896 | 0.045 |
| 118 | Rasgrp2 | -8.50 | -0.67 | 0.001051 | 0.034 |
| 119 | Zfp202 | -10.34 | -0.67 | 0.000494 | 0.024 |
| 120 | Bcl9l | -9.38 | -0.67 | 0.000718 | 0.028 |
| 121 | Prkra | -19.07 | -0.66 | 4.45E-05 | 0.010 |
| 122 | Hic2 | -20.97 | -0.66 | 3.05E-05 | 0.010 |
| 123 | 1700001L19Rik | -22.53 | -0.66 | 2.30E-05 | 0.009 |
| 124 | Ddx21 | -7.51 | -0.66 | 0.001681 | 0.042 |
| 125 | C230078M08Rik | -10.34 | -0.66 | 0.000495 | 0.024 |
| 126 | E030049G20Rik | -9.66 | -0.65 | 0.000643 | 0.027 |
| 127 | Zfp704 | -8.26 | -0.64 | 0.00117 | 0.036 |
| 128 | Bax | -15.15 | -0.64 | 0.000111 | 0.014 |
| 129 | Itpkc | -8.66 | -0.64 | 0.000978 | 0.033 |
| 130 | Igfbp4 | -8.84 | -0.64 | 0.000905 | 0.031 |
| 131 | Ppp1r1a | -9.39 | -0.64 | 0.000716 | 0.028 |
| 132 | Lgals1 | -7.49 | -0.64 | 0.001697 | 0.043 |
| 133 | Nasp | -10.27 | -0.63 | 0.000506 | 0.024 |
| 134 | Cd276 | -14.57 | -0.63 | 0.000129 | 0.015 |
| 135 | Cenpt | -7.47 | -0.63 | 0.00172 | 0.043 |
| 136 | 1810034K20Rik | -6.88 | -0.63 | 0.002334 | 0.049 |
| 137 | Tes | -8.03 | -0.63 | 0.001308 | 0.037 |
| 138 | Dnmt3b | -7.77 | -0.63 | 0.001477 | 0.040 |
| 139 | D11Ertd497e | -13.50 | -0.63 | 0.000174 | 0.016 |
| 140 | Efha1 | -12.08 | -0.62 | 0.000269 | 0.019 |
| 141 | Lrrc3 | -9.47 | -0.62 | 0.000692 | 0.028 |
| 142 | Dmrta2 | -9.89 | -0.61 | 0.000587 | 0.026 |
| 143 | Anapc5 | -10.72 | -0.61 | 0.000429 | 0.023 |
| 144 | 1200014J11Rik | -22.26 | -0.60 | 2.41E-05 | 0.009 |
| 145 | Zfp282 | -14.82 | -0.60 | 0.000121 | 0.014 |
| 146 | Fbp2 | -7.05 | -0.60 | 0.002139 | 0.047 |
| 147 | Cidea | -17.36 | -0.60 | 6.46E-05 | 0.012 |
| 148 | Armc10 | -7.65 | -0.60 | 0.001569 | 0.041 |
| 149 | Maged2 | -21.99 | -0.60 | 2.53E-05 | 0.009 |
| 150 | Fchsd1 | -17.60 | -0.59 | 6.12E-05 | 0.012 |
| 151 | Enc1 | -11.65 | -0.59 | 0.000311 | 0.020 |
| 152 | Eml4 | -10.07 | -0.59 | 0.000546 | 0.026 |
| 153 | Sfrs6 | -7.36 | -0.58 | 0.001817 | 0.044 |
| 154 | 6330514A18Rik | -8.04 | -0.58 | 0.001296 | 0.037 |
| 155 | Psmc3ip | -9.57 | -0.58 | 0.000667 | 0.028 |
| 156 | Rad9b | -13.42 | -0.58 | 0.000179 | 0.016 |
| 157 | Zkscan17 | -13.47 | -0.57 | 0.000176 | 0.016 |
| 158 | Chaf1b | -17.53 | -0.57 | 6.22E-05 | 0.012 |
| 159 | Phc1 | -12.28 | -0.57 | 0.000253 | 0.018 |
| 160 | 2310039H08Rik | -45.74 | -0.57 | 1.37E-06 | 0.007 |
| 161 | Rbm9 | -10.47 | -0.56 | 0.000471 | 0.024 |
| 162 | 9-Sep | -6.89 | -0.56 | 0.002328 | 0.049 |
| 163 | Arv1 | -10.90 | -0.56 | 0.000402 | 0.023 |
| 164 | Tspan6 | -6.80 | -0.56 | 0.002443 | 0.050 |
| 165 | Nln | -10.25 | -0.56 | 0.000511 | 0.025 |
| 166 | Sertad4 | -9.20 | -0.55 | 0.000775 | 0.029 |
| 167 | Stx7 | -11.74 | -0.55 | 0.000301 | 0.019 |
| 168 | Sh3md4 | -16.65 | -0.55 | 7.62E-05 | 0.012 |
| 169 | Txnrd3 | -8.30 | -0.55 | 0.00115 | 0.035 |
| 170 | Wdr48 | -19.27 | -0.55 | 4.27E-05 | 0.010 |
| 171 | Myo7a | -7.04 | -0.55 | 0.002147 | 0.047 |
| 172 | 2610018I03Rik | -7.89 | -0.54 | 0.001395 | 0.038 |
| 173 | Eif2s2 | -7.39 | -0.53 | 0.001792 | 0.044 |
| 174 | Rps19bp1 | -10.21 | -0.53 | 0.000518 | 0.025 |
| 175 | Ncan | -14.43 | -0.53 | 0.000134 | 0.015 |
| 176 | Ablim3 | -14.30 | -0.53 | 0.000139 | 0.015 |
| 177 | Nck2 | -7.30 | -0.53 | 0.001873 | 0.044 |
| 178 | Cldn16 | -7.35 | -0.53 | 0.001829 | 0.044 |
| 179 | Ankrd13b | -12.67 | -0.52 | 0.000223 | 0.017 |
| 180 | Ralgps2 | -6.94 | -0.52 | 0.002259 | 0.048 |
| 181 | Mta2 | -13.76 | -0.52 | 0.000162 | 0.016 |
| 182 | Vps33a | -15.80 | -0.52 | 9.38E-05 | 0.013 |
| 183 | Fsd1 | -7.97 | -0.52 | 0.001346 | 0.038 |
| 184 | Meig1 | -7.19 | -0.51 | 0.00198 | 0.045 |
| 185 | Gpx7 | -7.43 | -0.51 | 0.001755 | 0.043 |
| 186 | 2410137M14Rik | -9.57 | -0.51 | 0.000666 | 0.028 |
| 187 | EG331392 | -7.90 | -0.50 | 0.001389 | 0.038 |
| 188 | 3000004C01Rik | -7.79 | -0.50 | 0.001467 | 0.039 |
| 189 | Gpnmb | -6.92 | -0.50 | 0.002294 | 0.049 |
| 190 | 3110079O15Rik | -16.07 | -0.50 | 8.76E-05 | 0.013 |
| 191 | Pip5k2b | -8.56 | -0.50 | 0.001021 | 0.034 |
| 192 | Lzic | -7.26 | -0.49 | 0.001908 | 0.045 |
| 193 | Wnt7a | -12.90 | -0.49 | 0.000208 | 0.017 |
| 194 | Csda | -15.19 | -0.49 | 0.00011 | 0.014 |
| 195 | Zfp316 | -7.51 | -0.49 | 0.001681 | 0.042 |
| 196 | 1110017D15Rik | -8.28 | -0.48 | 0.001161 | 0.036 |
| 197 | BC089491 | -8.69 | -0.48 | 0.000965 | 0.032 |
| 198 | Phf5a | -25.89 | -0.48 | 1.32E-05 | 0.009 |
| 199 | Actl6b | -6.85 | -0.48 | 0.002376 | 0.049 |
| 200 | Btbd9 | -11.40 | -0.47 | 0.000338 | 0.021 |
| 201 | Epb4.1l4a | -7.13 | -0.47 | 0.002045 | 0.046 |
| 202 | Vit | -7.03 | -0.47 | 0.002161 | 0.047 |
| 203 | Cyb5b | -7.24 | -0.47 | 0.001933 | 0.045 |
| 204 | 1700012H17Rik | -9.93 | -0.47 | 0.000576 | 0.026 |
| 205 | Sez6 | -11.85 | -0.46 | 0.00029 | 0.019 |
| 206 | Nudt2 | -9.20 | -0.46 | 0.000776 | 0.029 |
| 207 | Rqcd1 | -7.64 | -0.46 | 0.001578 | 0.041 |
| 208 | Plekha8 | -14.38 | -0.46 | 0.000136 | 0.015 |
| 209 | Nfatc4 | -8.21 | -0.45 | 0.001199 | 0.036 |
| 210 | Ppp4r1 | -7.00 | -0.45 | 0.002193 | 0.047 |
| 211 | Mgst1 | -12.12 | -0.45 | 0.000266 | 0.019 |
| 212 | Cenpp | -21.76 | -0.45 | 2.64E-05 | 0.009 |
| 213 | Cdca7l | -10.69 | -0.45 | 0.000434 | 0.023 |
| 214 | Tmem39b | -7.85 | -0.45 | 0.001425 | 0.039 |
| 215 | Mfge8 | -6.85 | -0.45 | 0.002371 | 0.049 |
| 216 | BC057371 | -11.89 | -0.44 | 0.000287 | 0.019 |
| 217 | Tmem145 | -23.48 | -0.44 | 1.95E-05 | 0.009 |
| 218 | Morc2a | -9.78 | -0.44 | 0.000611 | 0.027 |
| 219 | Nipsnap3a | -7.00 | -0.44 | 0.002192 | 0.047 |
| 220 | Letmd1 | -12.06 | -0.43 | 0.000271 | 0.019 |
| 221 | Tkt | -11.01 | -0.43 | 0.000387 | 0.022 |
| 222 | Foxp1 | -8.18 | -0.43 | 0.001219 | 0.036 |
| 223 | Palld | -15.15 | -0.43 | 0.000111 | 0.014 |
| 224 | Pola2 | -7.27 | -0.43 | 0.001901 | 0.045 |
| 225 | Pigx | -7.55 | -0.43 | 0.001647 | 0.042 |
| 226 | Rims3 | -11.70 | -0.43 | 0.000305 | 0.019 |
| 227 | Parp6 | -7.28 | -0.43 | 0.001891 | 0.045 |
| 228 | Foxp2 | -7.15 | -0.43 | 0.002029 | 0.046 |
| 229 | Zfp420 | -7.11 | -0.42 | 0.002062 | 0.046 |
| 230 | Plk1 | -12.64 | -0.42 | 0.000225 | 0.017 |
| 231 | Lxn | -7.71 | -0.42 | 0.001527 | 0.040 |
| 232 | Ifngr2 | -8.44 | -0.42 | 0.001077 | 0.034 |
| 233 | Prr15 | -11.07 | -0.42 | 0.000379 | 0.022 |
| 234 | Aldh3a1 | -7.08 | -0.42 | 0.002106 | 0.047 |
| 235 | Fscn1 | -7.10 | -0.42 | 0.002081 | 0.046 |
| 236 | Tjp3 | -18.77 | -0.41 | 4.75E-05 | 0.010 |
| 237 | Pak7 | -7.56 | -0.41 | 0.001639 | 0.042 |
| 238 | Sec61a1 | -10.12 | -0.41 | 0.000537 | 0.026 |
| 239 | Cc2d1a | -27.50 | -0.41 | 1.04E-05 | 0.009 |
| 240 | Samd14 | -7.41 | -0.41 | 0.001766 | 0.043 |
| 241 | Armc3 | -8.97 | -0.41 | 0.000854 | 0.030 |
| 242 | Rg9mtd3 | -10.80 | -0.40 | 0.000418 | 0.023 |
| 243 | 1810006K21Rik | -12.00 | -0.40 | 0.000276 | 0.019 |
| 244 | Zfp324 | -7.46 | -0.40 | 0.001721 | 0.043 |
| 245 | Ltb4r1 | -7.25 | -0.40 | 0.00192 | 0.045 |
| 246 | Exoc2 | -11.13 | -0.39 | 0.000371 | 0.022 |
| 247 | Spc24 | -7.56 | -0.39 | 0.00164 | 0.042 |
| 248 | Mpp3 | -7.15 | -0.39 | 0.002022 | 0.046 |
| 249 | R74862 | -9.00 | -0.39 | 0.000842 | 0.030 |
| 250 | Faf1 | -13.63 | -0.39 | 0.000168 | 0.016 |
| 251 | Rfc4 | -10.46 | -0.39 | 0.000472 | 0.024 |
| 252 | Ckap2l | -8.32 | -0.39 | 0.001141 | 0.035 |
| 253 | Ttc22 | -9.22 | -0.39 | 0.00077 | 0.029 |
| 254 | 2310016C16Rik | -7.05 | -0.39 | 0.002129 | 0.047 |
| 255 | Kcnu1 | -8.74 | -0.38 | 0.000945 | 0.032 |
| 256 | Actr1a | -6.83 | -0.38 | 0.002409 | 0.050 |
| 257 | Aatf | -8.69 | -0.38 | 0.000964 | 0.032 |
| 258 | Kif26b | -6.82 | -0.38 | 0.002421 | 0.050 |
| 259 | Siah1a | -13.86 | -0.38 | 0.000157 | 0.016 |
| 260 | Plagl2 | -9.93 | -0.38 | 0.000577 | 0.026 |
| 261 | Stx11 | -10.07 | -0.37 | 0.000548 | 0.026 |
| 262 | Tnc | -6.85 | -0.37 | 0.002375 | 0.049 |
| 263 | Prss12 | -8.14 | -0.37 | 0.001239 | 0.036 |
| 264 | Plcl2 | -6.86 | -0.37 | 0.00237 | 0.049 |
| 265 | Runx1t1 | -18.67 | -0.37 | 4.85E-05 | 0.011 |
| 266 | Prkab1 | -7.57 | -0.37 | 0.001631 | 0.042 |
| 267 | Gldc | -7.82 | -0.37 | 0.001446 | 0.039 |
| 268 | Polr2i | -7.02 | -0.37 | 0.002164 | 0.047 |
| 269 | Tcp11 | -8.33 | -0.37 | 0.001133 | 0.035 |
| 270 | Thrap6 | -24.24 | -0.36 | 1.72E-05 | 0.009 |
| 271 | Mcpt6 | -7.80 | -0.36 | 0.001459 | 0.039 |
| 272 | Rbm8a | -10.88 | -0.36 | 0.000405 | 0.023 |
| 273 | Erlin2 | -6.88 | -0.36 | 0.002335 | 0.049 |
| 274 | Ube1x | -21.51 | -0.36 | 2.76E-05 | 0.009 |
| 275 | 4933428M03Rik | -7.80 | -0.36 | 0.00146 | 0.039 |
| 276 | Rcc1 | -9.70 | -0.36 | 0.000633 | 0.027 |
| 277 | Rad54l | -8.55 | -0.36 | 0.001025 | 0.034 |
| 278 | Csnk2a1 | -8.41 | -0.35 | 0.001096 | 0.035 |
| 279 | 2610002J02Rik | -9.85 | -0.35 | 0.000596 | 0.027 |
| 280 | Pias4 | -6.82 | -0.35 | 0.002417 | 0.050 |
| 281 | Ntsr1 | -7.45 | -0.35 | 0.001734 | 0.043 |
| 282 | Serf1 | -9.12 | -0.35 | 0.000801 | 0.029 |
| 283 | 1700045I19Rik | -7.02 | -0.35 | 0.002165 | 0.047 |
| 284 | 2500003M10Rik | -7.96 | -0.35 | 0.001352 | 0.038 |
| 285 | Zbtb39 | -17.60 | -0.34 | 6.12E-05 | 0.012 |
| 286 | Blm | -7.10 | -0.34 | 0.002082 | 0.046 |
| 287 | Rps24 | -7.38 | -0.34 | 0.001801 | 0.044 |
| 288 | Sncaip | -7.44 | -0.34 | 0.001745 | 0.043 |
| 289 | Mpped1 | -12.44 | -0.33 | 0.00024 | 0.018 |
| 290 | 5930416I19Rik | -7.34 | -0.33 | 0.001831 | 0.044 |
| 291 | Thoc3 | -8.18 | -0.33 | 0.001215 | 0.036 |
| 292 | Pitpna | -13.28 | -0.33 | 0.000186 | 0.016 |
| 293 | Nusap1 | -8.07 | -0.32 | 0.001283 | 0.037 |
| 294 | Prmt2 | -7.52 | -0.32 | 0.001676 | 0.042 |
| 295 | Trim47 | -8.34 | -0.32 | 0.00113 | 0.035 |
| 296 | Dhx37 | -8.95 | -0.32 | 0.000862 | 0 30 |
| 297 | V1rb2 | -7.70 | -0.32 | 0.001529 | 0.040 |
| 298 | Trim27 | -8.07 | -0.32 | 0.001281 | 0.037 |
| 299 | Elof1 | -9.94 | -0.32 | 0.000576 | 0.026 |
| 300 | Ppp4c | -7.12 | -0.32 | 0.002053 | 0.046 |
| 301 | Sval2 | -9.00 | -0.31 | 0.000843 | 0.030 |
| 302 | Grik1 | -7.11 | -0.31 | 0.002073 | 0.046 |
| 303 | Fusip1 | -6.94 | -0.31 | 0.002267 | 0.048 |
| 304 | A930005I04Rik | -7.47 | -0.31 | 0.001714 | 0.043 |
| 305 | Eid1 | -9.37 | -0.31 | 0.000722 | 0.028 |
| 306 | Olfr1361 | -7.65 | -0.30 | 0.001572 | 0.041 |
| 307 | Afg3l1 | -8.51 | -0.30 | 0.001047 | 0.034 |
| 308 | Mast4 | -10.89 | -0.30 | 0.000404 | 0.023 |
| 309 | Myst3 | -12.75 | -0.30 | 0.000218 | 0.017 |
