# Supplementary material for: Neonatal Maternal Deprivation Response and Developmental Changes in Gene Expression Revealed by Hypothalamic Gene Expression Profiling in Mice
Source: PLoS One. 2010 Feb 24;5(2):e9402. doi: 10.1371/journal.pone.0009402 (PMC2827556; doi:10.1371/journal.pone.0009402)
Supplement: Table S5 — Primers for real-time RT-PCR validation. (0.06 MB DOC) [file pone.0009402.s006.doc]

**Supplementary** **Table S5.** Primers for real-time RT-PCR validation

| Oligo Name | Sequence 5’ – 3’ |
| --- | --- |
| Actrt1-F | GAGATTGAACCTCGTCATGTCAT |
| Actrt1-R | GCTTCTTCTCCCACAAAGTACCT |
| Art5-F | AGCTCTACATGAGGCACTTCC |
| Art5-R | GCATTCCCAAACCTGCGAG |
| Brms1l-F | GAGCGGTTGAGTCAGGTGG |
| Brms1l-R | CCTTTGTGCGAATCTGCATGT |
| Chrm2-F | TGGTTTGGCTATTACCAGTCCT |
| Chrm2-R | CTGAAGGTGGCGGTTGACTT |
| Cryab-F | GTTCTTCGGAGAGCACCTGTT |
| Cryab-R | GAGAGTCCGGTGTCAATCCAG |
| Ctnnbl1-F | ATGGACGTGGGCGAACTTTTA |
| Ctnnbl1-R | CGCCATCCCTGTCAATAATCTG |
| Dhcr7-F | CTAAAGCTGCCCAACTGTATGC |
| Dhcr7-R | CGCCTCCCACATAACCTGG |
| Dlk1-F | GAAATAGACGTTCGGGCTTG |
| Dlk1-R | AGGGAGAACCATTGATCACG |
| FMO4-F | GATTGGAGCTGGCGTAAGTG |
| FMO4-R | TGTCAGCAAACTTCCACAGTC |
| Gfap-F | AGAAAACCGCATCACCATTC |
| Gfap-R | CCTTCTGACACGGATTTGGT |
| Gtl28d1-F | AAGAGAGCGTTTGTGACTGTC |
| Gtl28d1-R | GGAATGGTTTGGGCACCAC |
| Hnrpul1-F | CGCCTGAAGGTGAACGAACTT |
| Hnrpul1-R | GTAACCCCGGATCGTCGTC |
| Ing1l-F | CCCAAAGTGTAGGGGAGACA |
| Ing1l-R | TTAAAACCGATGGCCTTCAC |
| Kif21b-F | CTCACTGCTAAGTTTCACTTCGT |
| Kif21b-R | GCTTGGAGTCCCTGTAAGGC |
| Mett10d-F | GACAAACCACCTGACTTCGCA |
| Mett10d-R | TCTGACTGCTTCGGGGTCTT |
| Mospd3-F | CTGGTCTTTCCCCCGGATCTA |
| Mospd3-R | ACAGAACTCGGAAGCGAAGC |
| Mrgprb5-F | TATGTCTGCAACTTGGCTTGTG |
| Mrgprb5-R | CAGGGAACCTAAAATCTGAGTGC |
| Nanos2-F | AAGACTGAGCAGGACCTGGA |
| Nanos2-R | TCTTCAGCTGGTGTGAGGTG |
| Ndph-F | GCATCCATTTCTATGCTCTCCC |
| Ndph-R | GGTGTCTCATGCAGCGTTG |
| Nnat-F | CGGACTCCGAGACCAGTAGA |
| Nnat-R | AGCCGATGATGAGCAGTTCT |
| ORF9-F | CCAGTTGCTACAGGCATCTTC |
| ORF9-R | GGGGCACGTCAGGAATGAG |
| Pdk4-F | GGATGGAAGGAATCAAAGCA |
| Pdk4-R | GAAGGCACTGGCTTTTTGAG |
| Pgpep1-F | TCTGTCCCCATCCTTCTTTG |
| Pgpep1-R | CCCCAGGAGAAAGGAAAGAC |
| Pick1-F | CGCTAGGCGAGCCCTATAC |
| Pick1-R | CTTGGACATGGTGGATACGAAG |
| Pkd1-F | CTAGACCTGTCCCACAACCTA |
| Pkd1-R | GCAAACACGCCTTCTTCTAATGT |
| Prkag3-F | ACCAGCTCAGAAAGAACCTGT |
| Prkag3-R | GTGGCCTTCGGGAATGTGG |
| Prr8-F | TGCAGAACGGTCCTGGAGA |
| Prr8-R | CATCTGACAAATCCGACTGGTT |
| S100a1-F | AATGTGTTCCATGCCCATTCG |
| S100a1-R | ACCAGCACAACATACTCCTTG |
| Snx1-F | CACCGAGGGGGAGGATATTTT |
| Snx1-R | TCCAGGGATAGTTCCACTGTG |
| Suv420h1-F | CGGCTGCTTCCAACTCTACC |
| Suv420h1-R | AGTGATTCCGCAGTCTGATCT |
| Wscd2-F | CAGCGGTGTTTCCGGTATTTC |
| Wscd2-R | CAGGTGCAAATCACCCAGGA |
